# Comparative oral monotherapy of psilocybin, lysergic acid diethylamide, 3,4-methylenedioxymethamphetamine, ayahuasca, and escitalopram for depressive symptoms: systematic review and Bayesian network meta-analysis

**DOI:** 10.1136/bmj-2023-078607

**Published:** 2024-08-21

**Authors:** Tien-Wei Hsu, Chia-Kuang Tsai, Yu-Chen Kao, Trevor Thompson, Andre F Carvalho, Fu-Chi Yang, Ping-Tao Tseng, Chih-Wei Hsu, Chia-Ling Yu, Yu-Kang Tu, Chih-Sung Liang

**Affiliations:** 1 Department of Psychiatry, E-DA Dachang Hospital, I-Shou University, Kaohsiung, Taiwan; 2 Department of Psychiatry, E-DA Hospital, I-Shou University, Kaohsiung, Taiwan; 3 Graduate Institute of Clinical Medicine, College of Medicine, Kaohsiung Medical University, Kaohsiung, Taiwan; 4 Department of Neurology, Tri-Service General Hospital, National Defense Medical Centre, Taipei, Taiwan; 5 Department of Psychiatry, National Defense Medical Centre, Taipei, Taiwan; 6 Department of Psychiatry, Beitou Branch, Tri-Service General Hospital, Taipei, Taiwan; 7 Centre for Chronic Illness and Ageing, University of Greenwich, London, UK; 8 IMPACT (Innovation in Mental and Physical Health and Clinical Treatment) Strategic Research Centre, School of Medicine, Barwon Health, Deakin University, Geelong, VIC, Australia; 9 Institute of Biomedical Sciences, National Sun Yat-sen University, Kaohsiung, Taiwan; 10 Department of Psychology, College of Medical and Health Science, Asia University, Taichung, Taiwan; 11 Prospect Clinic for Otorhinolaryngology and Neurology, Kaohsiung, Taiwan; 12 Institute of Precision Medicine, National Sun Yat-sen University, Kaohsiung, Taiwan; 13 Department of Psychiatry, Kaohsiung Chang Gung Memorial Hospital and Chang Gung University College of Medicine, Kaohsiung, Taiwan; 14 Department of Pharmacy, Chang Gung Memorial Hospital Linkou, Taoyuan, Taiwan; 15 Institute of Health Data Analytics and Statistics, College of Public Health, National Taiwan University, Taipei, Taiwan; 16 Department of Dentistry, National Taiwan University Hospital, Taipei, Taiwan

## Abstract

**Objective:**

To evaluate the comparative effectiveness and acceptability of oral monotherapy using psychedelics and escitalopram in patients with depressive symptoms, considering the potential for overestimated effectiveness due to unsuccessful blinding.

**Design:**

Systematic review and Bayesian network meta-analysis.

**Data sources:**

Medline, Cochrane Central Register of Controlled Trials, Embase, PsycINFO, ClinicalTrial.gov, and World Health Organization’s International Clinical Trials Registry Platform from database inception to 12 October 2023.

**Eligibility criteria for selecting studies:**

Randomised controlled trials on psychedelics or escitalopram in adults with depressive symptoms. Eligible randomised controlled trials of psychedelics (3,4-methylenedioxymethamphetamine (known as MDMA), lysergic acid diethylamide (known as LSD), psilocybin, or ayahuasca) required oral monotherapy with no concomitant use of antidepressants.

**Data extraction and synthesis:**

The primary outcome was change in depression, measured by the 17-item Hamilton depression rating scale. The secondary outcomes were all cause discontinuation and severe adverse events. Severe adverse events were those resulting in any of a list of negative health outcomes including, death, admission to hospital, significant or persistent incapacity, congenital birth defect or abnormality, and suicide attempt. Data were pooled using a random effects model within a Bayesian framework. To avoid estimation bias, placebo responses were distinguished between psychedelic and antidepressant trials.

**Results:**

Placebo response in psychedelic trials was lower than that in antidepression trials of escitalopram (mean difference −3.90 (95% credible interval −7.10 to −0.96)). Although most psychedelics were better than placebo in psychedelic trials, only high dose psilocybin was better than placebo in antidepression trials of escitalopram (mean difference 6.45 (3.19 to 9.41)). However, the effect size (standardised mean difference) of high dose psilocybin decreased from large (0.88) to small (0.31) when the reference arm changed from placebo response in the psychedelic trials to antidepressant trials. The relative effect of high dose psilocybin was larger than escitalopram at 10 mg (4.66 (95% credible interval 1.36 to 7.74)) and 20 mg (4.69 (1.64 to 7.54)). None of the interventions was associated with higher all cause discontinuation or severe adverse events than the placebo.

**Conclusions:**

Of the available psychedelic treatments for depressive symptoms, patients treated with high dose psilocybin showed better responses than those treated with placebo in the antidepressant trials, but the effect size was small.

**Systematic review registration:**

PROSPERO, CRD42023469014.

## Introduction

Common psychedelics belong to two classes: classic psychedelics, such as psilocybin, lysergic acid diethylamide (known as LSD), and ayahuasca; and entactogens, such as 3,4-methylenedioxymethamphetamine (MDMA).^1^ Several randomised controlled trials have shown efficacy of psychedelics for people with clinical depression.^2^ ^3^ The proposed mechanism of its fast and persistent antidepressant effects is to promote structural and functional neuroplasticity through the activation of intracellular 5-HT_2A_ receptors in the cortical neurons.^4^ Additionally, the increased neuroplasticity was associated with psychedelic’s high affinity directly binding to brain derived neurotrophic factor receptor TrkB, indicating a dissociation between the hallucinogenic and plasticity promoting effects of psychedelics.^5^ A meta-analysis published in 2023 reported that the standardised mean difference of psychedelics for depression reduction ranged from 1.37 to 3.12,^2^ which are considered large effect sizes.^6^ Notably, the standardised mean difference of antidepressant trials is approximately 0.3 (a small effect size).^7^ ^8^

Although modern randomised controlled trials involving psychedelics usually use a double blinded design, the subjective effects of these substances can compromise blinding.^9^ Unsuccessful blinding may lead to differing placebo effects between the active and control groups, potentially introducing bias into the estimation of relative treatment effects.^10^ Concerns have arisen regarding the overestimated effect sizes of psychedelics due to the issues of blinding and response expectancy.^9^ Psychedelic treatment is usually administered with psychological support or psychotherapy, and thereby the isolated pharmacological effects of psychedelics remain to be determined.^2^ Surprisingly, on 1 July 2023, Australia approved psilocybin for the treatment of depression^11^; the first country to classify psychedelics as a medicine at a national level.

To date, only one double blind, head-to-head randomised controlled trial has directly compared a psychedelic drug (psilocybin) with an antidepressant drug (escitalopram) for patients with major depressive disorder.^12^ This randomised controlled trial reported that psilocybin showed a better efficacy than escitalopram on the 17 item Hamilton depression rating scale (HAMD-17).

We aimed to assess the comparative effectiveness and acceptability of oral monotherapy with psychedelics and escitalopram in patients experiencing depressive symptoms. Given that unsuccessful blinding can potentially lead to a reduced placebo response in psychedelic trials, we distinguished between the placebo responses in psychedelic and antidepressant trials. We also investigated the differences in patient responses between people who received extremely low dose psychedelics as a placebo and those who received a placebo in the form of a fake pill, such as niacin, in psilocybin trials.^13^ ^14^ Our study allowed for a relative effect assessment of psychedelics compared with placebo responses observed in antidepressant trials.

## Methods

The study protocol was registered with PROSPERO (CRD42023469014). We followed the Preferred Reporting Items for Systematic Reviews and Meta-Analyses (PRISMA) extension statement for reporting systematic reviews incorporating network meta-analysis (NMA) (appendix 1).^15^

### Data sources and searches

A comprehensive search of the Medline, Cochrane Central Register of Controlled Trials (CENTRAL), Embase, PsycINFO, ClinicalTrial.gov, and World Health Organization’s International Clinical Trials Registry Platform databases were performed without language restrictions from database inception to 12 October 2023. We also searched the grey literature and reviewed reference lists of the included studies and related systematic reviews.^2^ ^3^

### Study selection

Eligible studies were randomised controlled trials with parallel group or crossover designs. We included: (i) adults (≥18 years) with clinically diagnosed depression (eg, major depressive disorder, bipolar disorder, or other psychiatric disorders with comorbid clinical depression) or life threatening diagnoses and terminal illness with depressive symptoms; and (ii) adults with assessment of treatment response (preapplication/postapplication) using standard, validated, and internationally recognised instruments, such as HAMD-17. The outcome of interest was the change in depressive symptoms at the end of treatment compared with the controls, and we only extracted data from the first phase of crossover randomised controlled trials to avoid carry-over effects. Eligible psychedelic randomised controlled trials (including psilocybin, lysergic acid diethylamide, MDMA, and ayahuasca without dosage limit) required oral monotherapy without the concomitant use of antidepressants. For escitalopram, we included only fixed dose randomised controlled trials that compared at least two arms with different doses of oral form escitalopram (maximum dose of 20 mg/day) with placebo because psychedelic therapies usually use a fixed dose study design. We also included randomised controlled trials that evaluated psychedelic monotherapy compared with escitalopram monotherapy. We excluded follow-up studies and studies with healthy volunteers. We also excluded conference abstracts, editorials, reviews, meta-analyses, case reports, and case series, as well as publications reporting duplicate data. We did not consider ketamine because this drug is usually administered parenterally and is not a classic psychedelic.^16^ Screening and selection of the studies were performed independently by two authors. Discrepancies in study inclusion were resolved by deliberation among the reviewer pairs or with input from a third author. Appendix 2 shows the complete search strategies, and appendix 3 presents the reasons for exclusion.

### Definition of outcomes, data extraction, and risk of bias assessment

The primary outcome was change in depressive symptoms from baseline (continuous outcome), as measured by a validated rating scale, such as HAMD-17. When multiple measurement tools were used, they were selected in the following order: the HAMD-17, Montgomery-Åsberg depression rating scale, and Beck depression inventory (second edition). To improve interpretability, all extracted depression scores were converted to corresponding HAMD-17 scores using a validated method.^17^ We used a conservative correlation coefficient of 0.5 or other statistics (eg, t statistics) to calculate the standard deviation of change from baseline when unreported.^18^ The secondary outcomes were all cause discontinuation and severe adverse events (categorical outcomes). Severe adverse events were classified as those resulting in any of a list of negative health outcomes including, death, admission to hospital, significant or persistent incapacity, congenital birth defect or abnormality, and suicide attempt. Outcome data were extracted from original intention-to-treat or last observation carrying forward analysis, as well as from estimates of mixed-effect models for repeated measures.

Two authors independently extracted and reviewed the data, each being reviewed by another author. WebPlot Digitizer (https://automeris.io/WebPlotDigitizer/) was used to extract numerical data from the figures. Two authors independently used the Cochrane randomised trial risk of bias tool (version 2.0) to assess the risk of bias in the included trials, and discrepancies were resolved by consensus.^19^

### Data synthesis

To estimate the relative effect between two interventions, we computed mean difference on the basis of change values (with 95% credible interval) for continuous outcomes (change in depressive symptoms) and odds ratios for categorical outcomes (all cause discontinuation and severe adverse event). To assess the clinical significance of the relative effect, we evaluated whether the mean difference exceeded the minimal important difference, which is estimated to be 3 points for HAMD-17.^20^ We defined high, low, and extremely low doses of the included psychedelics as follows: (i) psilocybin: high dose (≥20 mg), extremely low dose (1-3 mg), low dose (other range); and (ii) MDMA: high dose (≥100 mg), extremely low dose (≤40 mg), low dose (other range). Escitalopram was divided into escitalopram 10 mg and escitalopram ≥20 mg. In previous clinical trials, a dose of 1 mg of psilocybin or a dose range of 1-3 mg/70 kg were used as an active control because these doses were believed not to produce significant psychedelic effects.^21^ ^22^ A dose of 5 mg/70 kg can produce noticeable psychedelic effects.^22^ In many two arm psilocybin trials, the psilocybin dose in the active group typically falls within the range of 20-30 mg.^12^ ^21^ ^23^ ^24^ In a three arm trial, 25 mg was defined as high dose, and 10 mg was considered a moderate dose.^21^ Another clinical trial also defined 0.215 mg/kg of psilocybin as a moderate dose for the active group.^25^ Therefore, we used 20 mg and 3 mg as the boundaries for grouping psilocybin doses; when the dosage was calculated per kilogram in the study, we converted it to per 70 kg. For MDMA, in two trials with three arms, 125 mg was defined as high dose, and 30-40 mg was defined as active control.^26^ ^27^ Thus, we used 100 mg and 40 mg as the boundaries for grouping MDMA doses.

We conducted random effects network meta-analysis and meta-analysis within a Bayesian framework.^28^ ^29^ Previous meta-analyses considered all control groups as a common comparator; however, concerns have been raised regarding the overestimated effect sizes of psychedelics because of unsuccessful blinding and poor placebo response.^9^ Therefore, we treated the three treatments as distinct interventions: the placebo response observed in psychedelic trials, the placebo response observed in antidepressant escitalopram trials, and extremely low dose psychedelics (ie, psilocybin and MDMA). We calculated the relative effects of all interventions compared with these three groups, indicating the following three conditions: (1) the treatment response of placebo response in the psychedelic trials is assumed to be lower than that of placebo response in antidepressant trials because of unsuccessful blinding.^9^ As such, the relative effects compared with placebo response in the psychedelic trials represented potential overestimated effect sizes. (2) the placebo response in antidepressant trials is assumed to be the placebo response in antidepressant trials with adequate blinding, therefore, the relative effects compared with placebo response in antidepressant trials represents effect sizes in trials with adequate blinding. (3) Psychedelic drugs are usually administered with psychotherapy^13^ or psychological support,^14^ the relative effects of psychedelics compared with extremely low dose psychedelics might eliminate the concomitant effects from psychotherapeutic support, approximating so-called pure pharmacological effects.

In network meta-analysis, the validity of indirect comparison relies on transitivity assumption.^30^ We assessed the transitivity assumption by comparing the distribution of potential effect modifies across treatment comparisons. In addition, we assessed whether the efficacy of escitalopram is similar in placebo controlled randomised controlled trials (escitalopram *v* placebo response in antidepressant trials) and in the head-to-head randomised controlled trial (psilocybin *v* escitalopram) using network meta-analysis.^12^ Furthermore, we assessed the efficacy of the different placebo responses (placebo response in the psychedelic trials *v* placebo response in antidepressant trials) as additional proof of transitivity. If the placebo response in antidepressant trials was better than that in the psychedelic trials, the transitivity assumption did not hold when grouping placebo response in antidepressant trials and placebo response in the psychedelic trials together. Finally, for the primary outcome (change in depressive symptoms), network meta-regression analyses were conducted to evaluate the impact of potential effect modifiers, including proportion of men and women in the study, mean age, baseline depression severity, disorder type, and follow-up assessment period. We assumed a common effect on all treatment comparisons for each of the effect modifiers. In other words, all interactions between the treatment comparisons and the effect modifier were constrained to be identical.

We also conducted the following sensitivity analyses: analysing studies of patients with major depressive disorder; excluding studies with a high risk of bias; adjusting for baseline depression severity; and using correlation coefficient of zero (most conservative) to calculate the standard deviation of change from baseline when unreported.

Publication bias was assessed by visual inspection of a comparison adjusted funnel plots. The first funnel plot used placebo response in the psychedelic trials as the comparator. The second funnel plot used placebo response in antidepressant trials as the comparator. The third funnel plot used both placebo response in the psychedelic trials and placebo response in antidepressant trials as comparators simultaneously. Additionally, we conducted the Egger test, Begg test, and Thompson test to examine the asymmetry of the third funnel plot. A previous meta-analysis reported that the standardised mean difference of psychedelics for depression reduction ranged from 1.37 to 3.12.^2^ Therefore, we also transformed the effect size of mean difference to standardised mean difference (Hedges’ g) for the primary outcome. The global inconsistency of the network meta-analysis was examined by fitting an unrelated main effects model. Local inconsistency of the network meta-analysis was examined using node splitting methods.^31^ Four Markov chains were implemented. 50 000 iterations occurred per chain and the first 20 000 iterations for each chain were discarded as a warm-up. Convergence was assessed by visual inspection of the trace plots of the key parameters for each analysis. The prior settings and convergence results are shown in appendix 4. All statistical analyses were done using R version 4.3.1. The network meta-analysis and pairwise meta-analysis within a Bayesian framework were fitted using the Bayesian statistical software called Stan within the R packages multinma^28^ and brms,^29^ respectively. The frequentist random effects network meta-analysis, funnel plots, and tests for funnel plot asymmetry were conducted using the R package netmeta. Reasons for protocol changes are in appendix 5.

### Assessment certainty of evidence for the primary outcome

The certainty of evidence produced by the network meta-analysis was evaluated using GRADE (Grading of recommendations, assessment, development and evaluation).^32^ ^33^ We used a minimally contextualised framework with the value of 3 (minimal important difference) as our decision threshold. The certainty of evidence refers to our certainty that the intervention had, relative to minimal intervention, any clinically minimal important difference. The optimal information size was calculated using a validated method.^32^ ^33^ ^34^

### Patient and public involvement

Both patients and the public are interested in research on novel depression treatments and their efficacy compared with existing antidepressants. However, due to a scarcity of available funding for recruitment and researcher training, patients and members of the public were not directly involved in the planning or writing of this manuscript. We did speak to patients about the study, and we asked a member of the public to read our manuscript after submission.

## Results

### Characteristics of included study

After searching the database and excluding duplicated records, we identified 3104 unique potential studies. We then screened the titles and abstracts of these studies for eligibility and excluded 3062 of them, in which 42 studies remained. Twenty six studies were excluded after an assessment of the full text for various reasons (appendix 3). We identified three additional studies through a manual search resulting in total 19 eligible studies (efigure 1). Details of the characteristics of the included studies are shown in etable 1. Protocols of psychological support or psychotherapy with psychedelic treatment are shown in etable 2. Overall, 811 people (mean age of 42.49 years, 54.2% (440/811) were women) were included in psychedelic trials (15 trials), and 1968 participants (mean age of 39.35 years, 62.5% (1230/1968) were women) were included in escitalopram trials (five trials).

### Risk of bias of the included studies

No psychedelic study (0/15) had a high overall risk of bias (efigure 2A and efigure 3A). The percentages of studies with high, some concerns, or low risk of bias in the 15 psychedelic trials were as follows: 0% (k=15), 33% (k=5), and 67% (k=10) for randomisation; 0% (k=0), 33% (k=5), and 67% (k=10) for deviations from intended interventions; 0% (k=0), 13% (k=2), and 87% (k=13) for missing outcome data; 0% (k=0), 33% (k=5), and 67% (k=10) for measurements of outcomes; 0% (k=0), 67% (k=1), and 93% (k=14) for selection of reported results. No non-psychedelic studies (0/5) were rated as high risk of bias (efigure 2B and efigure 3B). The percentages of studies with high, some concerns, and low risk of bias in the five non-psychedelic trials were as follows: 0% (k=0), 80% (k=4), and 20% (k=1) for randomisation; 0% (k=0), 100% (k=5), and 0% (k=0) for deviations from intended interventions; 0% (k=0), 80% (k=4), and 20% (k=1) for missing outcome data; 0% (k=0), 80% (k=4), and 20% (k=1) for measurements of outcomes; 0% (k=0), 20% (k=1), and 80% (k=4) for selection of reported results.

### Network meta-analysis

**Fig 1:**
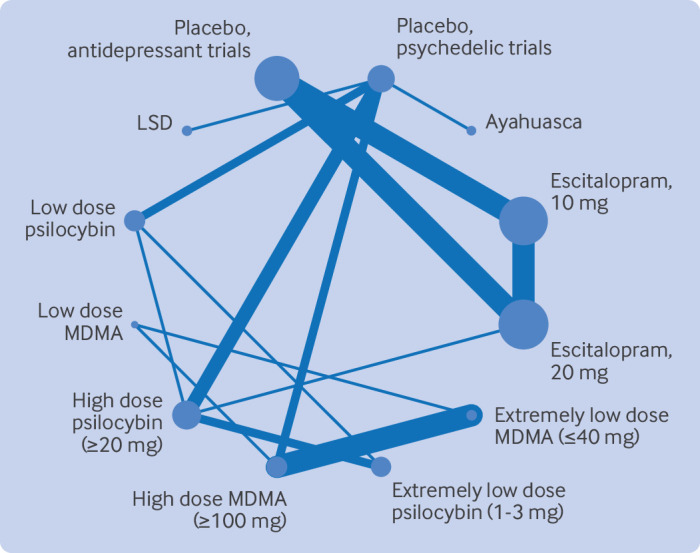
Network structure. LSD=lysergic acid diethylamide; MDMA=3,4-methylenedioxymethamphetamine

In the network structure, all interventions were connected, with two main structures (fig 1). All psychedelics were compared with placebo response in the psychedelic trials, and escitalopram was compared with placebo response in antidepressant trials. A head-to-head comparison of high dose psilocybin and 20 mg escitalopram connected the two main structures.^12^

**Fig 2:**
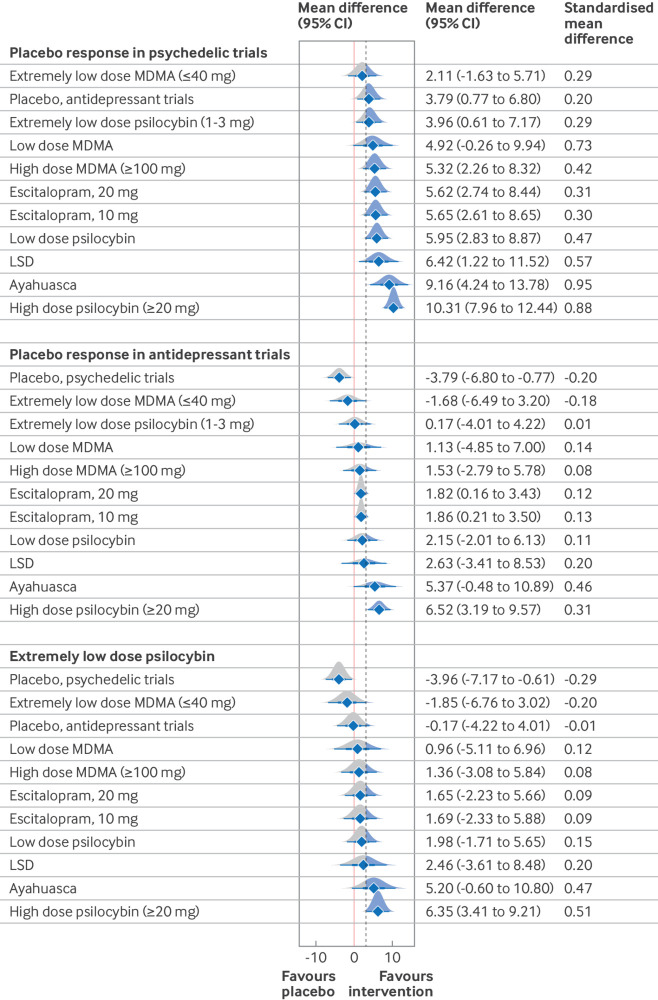
Forest plots of network meta-analytical estimates *v* different reference arms by observed placebo response. The dotted line represents the minimal important difference of 3 whereas the red line indicates 0. LSD=lysergic acid diethylamide; MDMA=3,4-methylenedioxymethamphetamine

In the main network meta-analysis, all interventions, except for extremely low dose and low dose MDMA, were associated with a larger mean difference exceeding the minimal important difference of 3 points on the HAMD-17 than with placebo response in the psychedelic trials (fig 2). Notably, placebo response in antidepressant trials (3.79 (95% credibile interval 0.77 to 6.80)) and extremely low dose psilocybin (3.96 (0.61 to 7.17)) were better than placebo response in the psychedelic trials, with mean differences exceeding 3 and 95% credibile intervals that did not cross zero. Additionally, in comparison with placebo response in antidepressant trials (fig 2), the relative effects of high dose psilocybin (6.52 (3.19 to 9.57)), escitalopram 10 mg (1.86 (0.21 to 3.50)), and escitalopram 20 mg (1.82 (0.16 to 3.43)) did not cross zero. Only high dose psilocybin resulted in a mean difference that was greater than 3. The standardised mean difference of high dose psilocybin decreased from large (0.88) to small (0.31) when the reference arm was changed from placebo response in the psychedelic trials to placebo response in antidepressant trials.

When compared with extremely low dose psilocybin (fig 2), only the relative effects of high dose psilocybin (6.35 (95% credibile interval 3.41 to 9.21)) and placebo response in the psychedelic trials (−3.96 (−7.17 to −0.61)) showed a larger mean difference exceeding 3, without crossing zero. All relative effects between interventions are showed in efigure 4. Importantly, the mean differences of high dose psilocybin compared with escitalopram 10 mg (4.66 (1.36 to 7.74); standardised mean difference 0.22), escitalopram 20 mg (4.69 (1.64 to 7.54); 0.24), high dose MDMA (4.98 (1.23 to 8.67); 0.32), and low dose psilocybin (4.36 (1.20 to 7.51); 0.32) all exceeded 3 and did not cross zero (efigure 4).

### Transitivity assumption

The assessment of transitivity assumption is showed in efigure 5 and efigure 6. We compared the efficacy of escitalopram in the placebo controlled antidepressant trials^8^ with that in the head-to-head trial (psilocybin *v* escitalopram)^12^ using network meta-analysis and pairwise meta-analysis. The results of the network meta-analysis showed that the relative effects between these two study designs (0.64 (95% credibile interval −4.41 to 5.40), efigure 6A; 1.94 (−2.66 to 6.14), efigure 6B) included zero, and the mean differences did not exceed 3. Placebo response in antidepressant trials was better than placebo response in the psychedelic trials with a small effect size (3.79 (0.77 to 6.80), standardised mean difference 0.2), and the mean difference exceed 3 (fig 2).

### Sensitivity analyses

**Fig 3:**
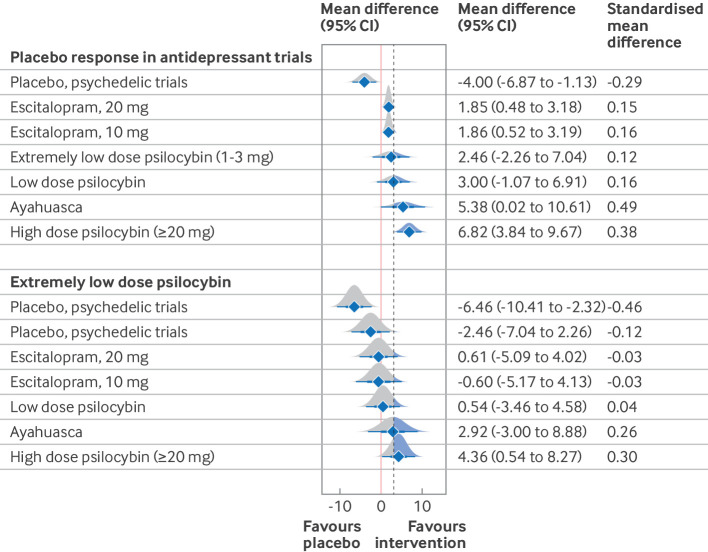
Forest plots of network meta-analytical estimates when considering a population with major depressive disorder

When including only patients with major depressive disorder, the relative effects of escitalopram 20 mg, escitalopram 10 mg, ayahuasca, and high dose psilocybin were better than placebo response in antidepressant trials, while placebo response in the psychedelic trials was worse than placebo response in antidepressant trials (fig 3). However, only the mean differences for high dose psilocybin (6.82 (95% credibile interval 3.84 to 9.67)), ayahuasca (5.38 (0.02 to 10.61)), and placebo response in the psychedelic trials (−4.00 (−6.87 to −1.13)) exceeded 3. When compared with extremely low dose psilocybin (excluding the effects from concomitant psychotherapeutic support), only the 95% credibile intervals of the relative effects of high dose psilocybin (4.36 (0.54 to 8.27); standardised mean difference 0.30) and placebo response in the psychedelic trials (−6.46 (−10.41 to −2.32), standardised mean difference −0.46) exceeded 3 and did not cross zero (fig 3). All of the relative effects between interventions are showed in efigure 7. Notably, the relative effects of high dose psilocybin compared with escitalopram 10 mg (4.96 (1.97 to 7.82)), escitalopram 20 mg (4.97 (2.19 to 7.64)), and low dose psilocybin (3.82 (0.61 to 7.04)) all exceeded 3 and did not cross zero (efigure 7).

The other three sensitivity analyses showed similar findings with the main analyses: exclusion of studies with high risk of bias (efigure 8); adjustment of baseline depression severity (efigure 9); and use of most conservative correlation coefficient of zero (efigure 10).

### All cause discontinuation and severe adverse event

When referencing placebo in psychedelic trials, no interventions were associated with higher risks of all cause discontinuation rate nor severe adverse event rate (efigure 11).

### Network meta-regression and publication bias

In network meta-regression analyses, the 95% credibile intervals of the relative effects of the baseline depressive severity, mean age, and percentage of women, crossed zero (etable 3). The results of the statistical tests (Egger, Begg, and Thompson-Sharp tests) for funnel plot asymmetry and visual inspection of funnel plots did not show publication bias (efigure 12). The results of GRADE assessment are provided in the efigure 13. Most of the certainty of evidence for treatment comparisons was moderate or low.

### Consistency assumptions

The back calculation methods for all the models (appendix 6) did not show any inconsistencies. The node splitting methods also did not show any inconsistencies (appendix 7).

## Discussion

### Principal findings

This network meta-analysis investigated the comparative effectiveness between psychedelics and escitalopram for depressive symptoms. Firstly, we found that the placebo response observed in antidepressant trials was associated with greater effectiveness than that observed in psychedelic trials. Secondly, when compared with placebo responses in antidepressant trials, only escitalopram and high dose psilocybin were associated with greater effectiveness, and only high dose psilocybin exceeded minimal important difference of 3. Notably, the effect size of high dose psilocybin decreased from large to small. Thirdly, among the included psychedelics, only high dose psilocybin was more likely to be better than escitalopram 10 mg or 20 mg, exceeding the minimally important difference of 3. Fourthly, in patients with major depressive disorder, escitalopram, ayahuasca, and high dose psilocybin were associated with greater effectiveness than placebo responses in antidepressant trials; however, only high dose psilocybin was better than extremely low dose psilocybin, exceeding minimal important difference of 3. Taken together, our study findings suggest that among psychedelic treatments, high dose psilocybin is more likely to reach the minimal important difference for depressive symptoms in studies with adequate blinding design, while the effect size of psilocybin was similar to that of current antidepressant drugs, showing a mean standardised mean difference of 0.3.^7^

### Comparison with other studies

In a randomised controlled trial, treatment response was defined as the response observed in the active arm; placebo response was defined as the response observed in the control (placebo) arm.^10^ Treatment response consists of non-specific effects, placebo effect, and true treatment effect; placebo response consisted of non-specific effects and placebo effect. Therefore, when the placebo effect is not the same for the active and control arms within an randomised controlled trial, the estimation of the true treatment effect is biased. For example, in a psychedelic trial, unsuccessful blinding may occur due to the profound subjective effects of psychedelics. This unblinding may lead to high placebo effect in the active arm and low placebo effect in the control arms, and the true treatment effect is overestimated.^10^ Without addressing unequal placebo effects within studies, the estimation of meta-analysis and network meta-analysis are biased.^10^ However, in most psychedelic trials, blinding was either reported as unsuccessful or not assessed at all. For example, two trials of lysergic acid diethylamide reported unsuccessful blinding,^35^ ^36^ whereas the trial of ayahuasca only reported that five of 10 participants misclassified the placebo as ayahuasca.^37^ In trials of MDMA, participants' accuracy in guessing which treatment arm they were in ranged from approximately 60-90%.^26^ ^27^ ^38^ ^39^ ^40^ In the case of most psilocybin trials, blinding was not assessed, with the exception of the study by Ross and colleagues in 2016.^13^ In that study, participants were asked to guess whether the psilocybin or an active control was received, and the correct guessing rate was 97%. In our study, we established several network meta-analysis models addressing this issue, and we found that placebo response in the psychedelic trials was associated with less effectiveness than that in antidepressant trials. Therefore, the effect sizes of psychedelics compared with placebo response observed in psychedelic trials may be overestimated. All of the psychedelics’ 95% credibile intervals of the relative effects crossed zero when compared with the placebo response in antidepressant trials, except for high dose psilocybin.

The comparisons between psychedelics and escitalopram showed that high dose psilocybin was more likely to be better than escitalopram. Psilocybin was usually administered with psychotherapy or psychological support.^13^ ^14^ Therefore, the greater effectiveness of psilocybin may be from not only pharmacological effects but also psychotherapeutic support. However, we also found that high doses of psilocybin was associated greater effectiveness than extremely low doses of psilocybin. This effect also indicates that the effectiveness of psilocybin cannot be attributed only to concomitant psychotherapy or psychological support.

In patients with major depressive disorder, ayahuasca, low dose psilocybin, high dose psilocybin, escitalopram 10 mg, and escitalopram 20 mg were associated with greater effectiveness than the placebo response in antidepressant trials . However, when compared with extremely low dose psilocybin, only high dose psilocybin was associated with better effectiveness; the standardised mean difference decreased from 0.38 (compared with placebo response in antidepressant trials) to 0.30 (compared with extremely low dose psilocybin). As such, the effectiveness of psilocybin should be considered with concomitant psychotherapeutic support in people with major depressive disorder. The effect size of high dose psilocybin was similar with antidepressant trials of patients with major depressive disorder showing a mean standardised mean difference of 0.3.^7^ ^8^

### Strengths and limitations of this study

This study has several strengths. We conducted separate analyses for placebo response in antidepressant trials, placebo response in psychedelic trials, and an extremely low active dose of psychedelics, thereby mitigating the effect of placebo response variations across different studies. This approach allowed us to assess the efficacy of psychedelics more impartially and make relatively unbiased comparisons than if these groups were not separated. This study supported the transitivity assumption of the efficacy of escitalopram in placebo controlled antidepressant trials with that in psilocybin versus escitalopram head-to-head trial, thereby bridging the escitalopram trials and psychedelic trials. We also performed various sensitivity analyses to ensure the validation of our statistical results.

Nevertheless, our study has several limitations. Firstly, we extracted only the acute effects of the interventions. A comparison of the long term effects of psychedelics and escitalopram remains unclear. Secondly, participants in the randomised controlled trials on MDMA were predominantly diagnosed with post-traumatic stress disorder, whereas participants in the randomised controlled trials on escitalopram were patients with major depressive disorder. However, depressive symptoms in post-traumatic stress disorder could be relatively treatment resistant, requiring high doses of psychotropic drugs.^41^ Moreover, our study focused not only on major depressive disorder but also on the generalisability of psychedelic treatment for depressive symptoms. Thirdly, although all available studies were included, the sample size of the psychedelic randomised controlled trials was small (k=15). Fourthly, when using extremely low dose psychedelics as a reference group, the relative effect may also eliminate some pharmacological effects because our study found that extremely low dose psychedelics could not be considered a placebo. Fifthly, in network meta-analysis, direct evidence for one treatment comparison may serve as indirect evidence for other treatment comparisons,^42^ and biases in the direct evidence might affect estimates of other treatment comparisons. Because the absolute effect of escitalopram in the head-to-head trial (high dose psilocybin *v* escitalopram 20 mg)^12^ was lower than those of placebo controlled trials, the relative effects of high dose psilocybin might be slightly overestimated when compared with other treatments in the current study. We addressed this issue by use of a Bayesian network meta-analysis, distinguishing between placebo response in psychedelic trials and placebo response in antidepressant trials. Specifically, we only considered that the 95% credibile interval of the relative effect between two comparisons did not cross zero. Indeed, the relative effect of escitalopram 20 mg between these two study designs included zero. Finally, our network meta-analysis may not have sufficient statistical power to detect potential publication bias due to the scarcity of trials and participants.

### Implications and conclusions

Serotonergic psychedelics, especially high dose psilocybin, appeared to have the potential to treat depressive symptoms. However, study designs may have overestimated the efficacy of psychedelics. Our analysis suggested that the standardised mean difference of high dose psilocybin was similar to that of current antidepressant drugs, showing a small effect size. Improved blinding methods and standardised psychotherapies can help researchers to better estimate the efficacy of psychedelics for depressive symptoms and other psychiatric conditions.

**What is already known on this topic**

Psychedelic treatment resulted in significant efficacy in treating depressive symptoms and alleviating distress related to life threatening diagnoses and terminal illness
Meta-analyses have reported standardised mean difference of psychedelics for depression reduction ranging from 1.37 to 3.12, while antidepressant trials were approximately 0.3
No network meta-analysis has examined comparative efficacy between psychedelics and antidepressants for depressive symptoms, and effect sizes of psychedelics might be overestimated because of unsuccessful blinding and response expectancies

**What this study adds**

To avoid estimation bias, placebo responses in psychedelic and antidepressant trials were separated; placebo response in psychedelic trials was lower than that in antidepressant trials
Among all psychedelics studied, only high dose psilocybin was associated with greater effectiveness than placebo response in antidepressant trials (standardised mean difference 0.31)
Among all psychedelics, only high dose psilocybin was associated with greater effectiveness than escitalopram

## Data Availability

The data that support the findings of this study are available from the corresponding author (C-SL) upon reasonable request.

## Web extra

Extra material supplied by authors

Web appendix: Extra material supplied by authors

## Notes

Contributors: T-WH and C-KT contributed equally to this work and are joint first authors. Y-KT and C-SL contributed equally to this work and are joint last/corresponding authors. C-SL, T-WH, and Y-KT conceived and designed the study. T-WH, C-KT, C-WH, and P-TT selected the articles, extracted the data, and assess the risk of bias. C-LY did the systemic search. T-WH and C-SL wrote the first draught of the manuscript. TT, AFC, Y-CK, F-CY, and Y-KT interpreted the data and contributed to the writing of the final version of the manuscript. C-KT and T-WH have accessed and verified the data. C-SL and Y-KT were responsible for the decision to submit the manuscript. All authors confirmed that they had full access to all the data in the study and accept responsibility to submit for publication. The corresponding author attests that all listed authors meet authorship criteria and that no others meeting the criteria have been omitted.

Transparency: The lead author (C-SL) affirms that the manuscript is an honest, accurate, and transparent account of the study being reported; that no important aspects of the study have been omitted; and that any discrepancies from the study as planned

Dissemination to participants and related patient and public communities: Dissemination of the work to the public and clinical community through social media and lectures is planned.

Provenance and peer review: Not commissioned; externally peer reviewed.
