# Supplementary material for: Comparative oral monotherapy of psilocybin, lysergic acid diethylamide, 3,4-methylenedioxymethamphetamine, ayahuasca, and escitalopram for depressive symptoms: systematic review and Bayesian network meta-analysis
Source: BMJ. 2024 Aug 21;386:e078607. doi: 10.1136/bmj-2023-078607 (PMC11337322; doi:10.1136/bmj-2023-078607)
Supplement: Supplementary file 1 — Web appendix: Extra material supplied by authors [file hsut078607.ww.pdf]

**Comparative oral monotherapy of psilocybin, LSD, MDMA, ayahuasca, and escitalopram for depressive symptoms: a systematic review and Bayesian network meta-analysis**

eTable 1. Demographics and clinical characteristics of the included studies
eTable 2. Protocols of Psychological Support or Psychotherapy with Psychedelic Treatment
eTable 3. Network meta-regression
eFigure 1. Study flow chart
eFigure 2. Risk of bias plot, A) psychedelic studies; B) non-psychedelic studies
eFigure 3. Summary of risk of bias, A) psychedelic studies; B) non-psychedelic studies
eFigure 4. All comparative comparisons of all included interventions
eFigure 5. Transitivity assumption of study characteristics by direct treatment comparisons
eFigure 6. Transitivity assumption of escitalopram effectiveness between two study designs
eFigure 7. All comparative comparisons of all included interventions in patients with major depressive disorder
eFigure 8. Sensitivity analysis 2: excluding studies with high risk of bias
eFigure 9. Sensitivity analysis 3: adjusting for baseline depression severity
eFigure 10. Sensitivity analysis 4: using most conservative correlation coefficient of zero
eFigure 11. Forest plots of NMA estimates of all-cause discontinuation and severe adverse event
eFigure 12. Funnel plots (order by placebo used in psychedelic trials)
eFigure 13. GRADE assessment for the primary outcome
Appendix 1. The PRISMA
Appendix 2. The complete search strategies
Appendix 3. Reasons for exclusion
Appendix 4. Prior settings and results of convergence
Appendix 5. Reasons for protocol changes
Appendix 6. The back-calculation methods for all the models
Appendix 7. Not-splitting methods

**eTable 1. Demographics and clinical characteristics of the included studies**

| Author, year, country | Study design | Subjects characteristics | Intervention, doses | N, N of female | Age (years) | Sessions | Post-treatment assessment time | Baseline depression severity |
|---|---|---|---|---|---|---|---|---|
| Grob 2011, USA | DBRCT | patients with advanced cancer | Psilocybin, 0.2mg/kg | 6, (all) 11 | 36-58 | 1 | 2 weeks | (BDI) 16.1 |
| | | | Niacin 250mg | 6, (all) 11 | | | | 14.3 |
| Griffiths 2016, USA | DBRCT-CO | Patients with life-threatening cancer | Psilocybin 22 or 30mg / 70kg | 26, 13 | 56.5 | 2 (1 for each arm) | 5 weeks | (HAMD-17) 22.8 |
| | | | Psilocybin 1 or 3mg/ 70kg | 25, 12 | 56.1 | | | 22.3 |
| Ross 2016, USA | DBRCT-CO | Patients with life-threatening cancer | Psilocybin, 0.3mg/kg | 14, 7 | 52.0 | 2 (1 for each arm) | 7 weeks | (BDI) 15.0 |
| | | | Niacin 250mg | 15, 11 | 60.3 | | | 16.7 |
| Carhart-Harris 2021, UK | DBRCT | MDD | Psilocybin 25mg+ placebo | 30, 11 | 42.3 | 2 for psilocybin; 6 weeks escitalopram/ placebo | 6 weeks | (HAMD-17) 19.2 |
| | | | Psilocybin 1mg+ escitalopram 10mg (first 3 weeks)/ 20mg (later 3 weeks) | 29, 9 | 39.1 | | | 18.4 |
| Davis 2021, USA | DBRCT | MDD | Psilocybin 20mg/70kg; 30mg/70mg | 13, 9 | 43.6 | 2 | 5 weeks | (HAMD-17) 22.9 |
| | | | Delayed control | 11, 7 | 35.2 | | | 22.5 |
| Goodwin 2022, USA | DBRCT | TRD | Psilocybin 25mg | 79, 44 | 40.2 | 1 | 12 weeks | (MADRS) 31.9 |
| | | | Psilocybin 10mg | 75, 41 | 40.6 | | | 33.0 |
| | | | Psilocybin 1mg | 79, 36 | 38.7 | | | 32.7 |
| von Rotz 2023, Switzerland | DBRCT | MDD | Psilocybin 0.215mg/kg | 26, 16 | 37.6 | 1 | 2 weeks | (MADRS) 24.3 |
| | | | Placebo | 26, 17 | 35.9 | | | 24.1 |
| Raison 2023, | DBRCT | MDD | Psilocybin 25mg | 51, 24 | 40.4 | 1 | 6 weeks | (MADRS) 35.5 |

| | | | | | | | | |
|---|---|---|---|---|---|---|---|---|
| USA | | | Niacin 250mg | 53, 28 | 41.8 | | | 35.0 |
| Holze 2023, Switzerland | DBRCT-CO | anxiety with cancer | LSD 200 mcg | 20, 9 | 45.0 | 4 | 10 weeks | (HAMD-21) 18.0 |
| | | | Placebo | 22, 11 | 46.0 | | | 20.0 |
| Palhano-Fontes 2019, Brazil | DBRCT | TRD | Ayahuasca, 0.36mg/kg N,N-DMT | 17, 11 | 39.7 | 1 | 1 week | (HAMD-17) 24.1 |
| | | | Placebo | 18, 10 | 44.2 | | | 19.7 |
| Mithoefer 2018, USA | DBRCT | PTSD | MDMA 125mg | 12, 4 | 40.7 | 2 | 8 weeks | (BDI-II) 36.6 |
| | | | MDMA 75mg | 7, 1 | 29.1 | | | 24.7 |
| | | | MDMA 30mg | 7, 2 | 39.2 | | | 30.4 |
| Ot'alora 2018, USA | DBRCT | PTSD | MDMA 125mg | 13, 8 | 44.6 | 2 | 8 weeks | (BDI-II) 29.3 |
| | | | MDMA 100mg | 9, 6 | 39.6 | | | 28.2 |
| | | | MDMA 40mg | 6, 5 | 40.0 | | | 23.8 |
| Mitchell 2021, Canada, Israel, USA | DBRCT | PTSD | MDMA 80-180mg | 46, 27 | 43.5 | 3 | 18 weeks, | (BDI-II) 30.5 |
| | | | Placebo | 44, 32 | 38.2 | | | 334.9 |
| Wolfson 2020, USA | DBRCT | life-threatening disease | MDMA 125mg | 13, 10 | 55.5 | 2 | 8 weeks | (MADRS) 19.5 |
| | | | Placebo | 5, 4 | 53.2 | | | 19.2 |
| NCT01689740, Israel | DBRCT | PTSD | MDMA 125mg | 5, 3 | 18-65 | 2 | 8 weeks | (BDI-II) Not reported |
| | | | MDMA 25mg | 3, 0 | | | | Nor reported |
| Burke 2002, USA | DBRCT | MDD | Escitalopram 20mg | 123, 84 | 39.6 | 8 weeks, once dose daily | 8 weeks | (HAMD-17) 25.8 |
| | | | Escitalopram 10mg | 118, 83 | 40.7 | | | 24.3 |
| | | | Placebo | 119, 71 | 40.1 | | | 25.8 |
| Hirayasu 2011a, Japan | DBRCT | MDD | Escitalopram 10mg | 105, (all) 177 | 43.6 | 8 weeks, once dose daily | 8 weeks | (HAMD-17) 22.2 |

| | | | | | | | | |
|---|---|---|---|---|---|---|---|---|
| | | | Escitalopram 20mg | 100, (all) 177 | | | | 22.3 |
| | | | Placebo | 105, (all) 177 | | | | 22.5 |
| Hirayasu 2011b, Japan | DBRCT | MDD | Escitalopram 20mg | 120,(all) 207 | 43.6 | 8 weeks, once dose daily | 8 weeks | (MADRS) 29.8 |
| | | | Escitalopram 10mg | 119,(all) 207 | | | | 29.4 |
| | | | Placebo | 124,(all) 207 | | | | 29.0 |
| NCT00668525, USA | DBRCT | MDD | Escitalopram 20mg | 319, 222 | 40.4 | 8 weeks, once dose daily | 8 weeks, none | (HAMD-24) 29.3 |
| | | | Escitalopram 10mg | 318, 205 | 41.4 | | | 29.3 |
| | | | Placebo | 215, 137 | 42.3 | | | 28.9 |

N, number; DBRCT, double-blind randomised controlled trials; DBRCT-CO, double-blind randomised controlled, cross-over trial; SBRCT-CO, single-blind randomised controlled, cross-over trial; MDD, major depressive disorder; TRD, treatment-resistant depression; PTSD, Post-traumatic stress disorder; LSD, Lysergic Acid Diethylamide; N,N-DMT, N,N-dimethyltryptamine; BDI, Beck Depression Inventory; HAMD: Hamilton Depression Rating Scale; MADRS, Montgomery and Asberg Depression Rating Scale; HADS-D, Hospital Anxiety and Depression Scale-Depression part

* When the exact number of females in each arm was unknown, we indicated the total number of females in the studies using (all).

**Included studies**


Burke WJ, Gergel I, Bose A. Fixed-dose trial of the single isomer SSRI Escitalopram in depressed outpatients. *The Journal of Clinical Psychiatry* 2002;63(4):331-36. doi: 10.4088/JCP.v63n0410

Carhart-Harris R, Giribaldi B, Watts R, et al. Trial of Psilocybin versus Escitalopram for Depression. *New England journal of medicine* 2021;384(15):1402-11. doi: 10.1056/NEJMoa2032994

Davis AK, Barrett FS, May DG, et al. Effects of Psilocybin-Assisted Therapy on Major Depressive Disorder: a Randomized Clinical Trial. *JAMA psychiatry* 2021;78(5):481-89. doi: 10.1001/jamapsychiatry.2020.3285

Goodwin GM, Aaronson ST, Alvarez O, et al. Single-Dose Psilocybin for a Treatment-Resistant Episode of Major Depression. *The New England journal of medicine* 2022;387(18):1637-48. doi: https://dx.doi.org/10.1056/NEJMoa2206443

Griffiths RR, Johnson MW, Carducci MA, et al. Psilocybin produces substantial and sustained decreases in depression and anxiety in patients with life-threatening cancer: a randomized double-blind trial. *Journal of psychopharmacology (Oxford, England)* 2016;30(12):1181-97. doi: 10.1177/0269881116675513

Grob CS, Danforth AL, Chopra GS, et al. Pilot study of psilocybin treatment for anxiety in patients with advanced-stage cancer. *Archives of General Psychiatry* 2011;68(1):71-78. doi: 10.1001/archgenpsychiatry.2010.116

Holze F, Gasser P, Muller F, et al. Lysergic Acid Diethylamide-Assisted Therapy in Patients With Anxiety With and Without a Life-Threatening Illness: a Randomized, Double-Blind, Placebo-Controlled Phase II Study. *Biological psychiatry* 2023;93(3):215-23. doi: 10.1016/j.biopsych.2022.08.025

Hirayasu, Y. A dose-response study of escitalopram in patients with major depressive disorder: a placebo-controlled, double-blind study. *Jpn J Clin Psychopharmacol* 2011; 14: 871.

Hirayasu Y. A dose-response and non-inferiority study evaluating the efficacy and safety of escitalopram in patients with major depressive disorder: a placebo- and paroxetine-controlled, double-blind, comparative study. *Jpn J Clin Psychopharmacol* 2011;14:883-99.

Mitchell JM, Bogenschutz M, Lilienstein A, et al. MDMA-assisted therapy for severe PTSD: a randomized, double-blind, placebo-controlled phase 3 study. *Nature medicine* 2021;27(6):1025-33. doi: 10.1038/s41591-021-01336-3

Mithoefer MC, Mithoefer AT, Feduccia AA, et al. 3,4-methylenedioxymethamphetamine (MDMA)-assisted psychotherapy for post-traumatic stress disorder in military veterans, firefighters, and police officers: A randomised, double-blind, dose-response, phase 2 clinical trial. *The Lancet Psychiatry* 2018;5(6):486-97. doi: 10.1016/S2215-0366(18)30135-4

Ot'alora G M, Grigsby J, Poulter B, et al. 3,4-Methylenedioxymethamphetamine-assisted psychotherapy for treatment of chronic posttraumatic stress disorder: A randomized phase 2 controlled trial. *Journal of Psychopharmacology* 2018;32(12):1295-307. doi: 10.1177/0269881118806297

Palhano-Fontes F, Barreto D, Onias H, et al. Rapid antidepressant effects of the psychedelic ayahuasca in treatment-resistant depression: A randomized placebo-controlled trial. *Psychological Medicine* 2019;49(4):655-63. doi: 10.1017/S0033291718001356

Raison CL, Sanacora G, Woolley J, et al. Single-Dose Psilocybin Treatment for Major Depressive Disorder: A Randomized Clinical Trial. *JAMA* 2023 doi: 10.1001/jama.2023.14530

Ross S, Bossis A, Guss J, et al. Rapid and sustained symptom reduction following psilocybin treatment for anxiety and depression in patients with life-threatening cancer: a randomized controlled trial. *Journal of psychopharmacology (Oxford, England)* 2016;30(12):1165-80. doi: 10.1177/0269881116675512

von Rotz R, Schindowski EM, Jungwirth J, et al. Single-dose psilocybin-assisted therapy in major depressive disorder: a placebo-controlled, double-blind, randomised clinical trial. *EClinicalMedicine* 2023;56:101809. doi: 10.1016/j.eclinm.2022.101809

Wolfson PE, Andries J, Feduccia AA, et al. MDMA-assisted psychotherapy for treatment of anxiety and other psychological distress related to life-threatening illnesses: a randomized pilot study. *Scientific reports* 2020;10(1):20442. doi: 10.1038/s41598-020-75706-1

https://clinicaltrials.gov/study/NCT00668525

https://clinicaltrials.gov/study/NCT01689740

## eTable 2. Protocols of Psychological Support or Psychotherapy with Psychedelic Treatment

| Author | Model | Therapist | Preparation | Integration |
|---|---|---|---|---|
| Grob (2011) | Nondirective psychological support with no manual specified | No mentioned | No mentioned | Discussion of the subjective aesthetic, cognitive, affective, and psychospiritual experiences they had during the session |
| Carhart-Harris (2016) | Nondirective psychological support with no manual specified | 2 psychiatrists | Preparation: trust, relationship, expectance<br>Support (Acute and peri-acute): empathetic listening and reassurance | Non-judgmental listening to the patient's testimony after his/her experience and may occasionally feature some interpretation regarding the content of the experience and its potential meaning, as well as advice regarding maintaining and cultivating positive changes in outlook and lifestyle.<br>By telephone the day after low-dose administration; in person the day after the high-dose administration; another visit 1 week after the high-dose administration |
| Griffiths (2016) | Nondirective psychological support | Trained by an experienced psychologist | Discussion of<br>meaningful aspects of the participant's life, served to establish rapport | During sessions, monitors were nondirective and supportive, and they encouraged participants to "trust, let go and be open" to the experience. Meetings after sessions generally focused on novel thoughts and feelings that arose during sessions |
| Ross (2016) | Preparatory psychotherapy, medication dosing sessions, and post-dosing integrative psychotherapy with specified manual (component: supportive, cognitive-behavioral, existentially oriented, and psycho-dynamic/ psychoanalytic) | 6 psychiatrists, 2 psychologists, 4 social workers, 1 nurse, 2 master-level counselors | Reviewing the purpose and intention, establishing rapport and trust, and reviewing the life histories (three psychotherapeutic preparation sessions) | Therapists were available for psychological and medical support during the sessions. Participants were encouraged to bring in items of personal significance and meaning and to direct attention to internal experience after psilocybin administration. Towards the end of the dosing session, participants were encouraged to discuss the entirety of their subjective experience with the treatment team to consolidate the memory of the experience (especially given that these experiences can often be ephemeral and difficult to recall in detail, not dissimilar from dream states) and to begin the process of post-integrative psychotherapy. This was akin to doing psychoanalytic psychotherapy with a patient in a type of waking dream-like state. Following each treatment session, three 2-hour psychotherapy sessions were conducted to further consolidate the memory of the experience and to continue the process of psychological integration. |
| Carhart- | Nondirective psychological | Egalitarian guides | Toward a sense of greater understanding, meaning, and purpose | Debriefs: both guides were present for the in-person integration sessions, which |

| Author | Model | Therapist | Preparation | Integration |
|---|---|---|---|---|
| Harris (2021) | support (emotional support and trust; music listening; therapeutic intention; psychological flexibility; accepted, connect, embody model) | | (i.e., painful content) | entailed open, attentive listening to the participant's account of his/her session content. Participants then had the option of engaging in a visualisation exercise to facilitate access to emotions they had experienced during the psilocybin session. Further Integration calls took place with the lead guide, totalling six integration calls in total during the trial. |
| Davis (2021) | Supportive psychotherapy | Facilitators with varying educational level (i.e., bachelor's, master's, doctorate, and medical degrees) and professional disciplines (e.g., social work, psychology, and psychiatry) | Developing rapport and trust; reviewing the life histories and current situation; discussing intentions and expectations | The participant will meet with the session monitor(s) one day and one week after each session and four weeks after the second session to support integration of session-day experiences.<br>As with any acute, intense positive or negative emotional experience, participants often feel the need for, and seem to benefit from, additional time for reflecting on the novel thoughts and feelings that may have arisen in the session. Given the potentially intense and unusual psychological nature of hallucinogen effects, the volunteer may have difficulty discussing the experience with others in her or his life. Because the monitors were present during the session when the hallucinogen effects were experienced and have knowledge of a broad range of reported phenomena during drug action, the volunteer may feel more comfortable discussing her or his experiences with the monitors than with others. This follow up contact also allows the assessment of any potentially persisting adverse effects, including perceptual abnormalities. |
| Goodwin (2022) | Nondirective psychological support; manualized | Two therapists (master's-level practitioners, nurses, diploma-level cognitive behavioral therapists, or | Two sessions for building trust, receiving psychoeducation, and preparing for the psychedelic experience | To support participants in deriving their own insights and solutions from the experience with psilocybin. Therapists were advised to remain open and supportive, without active guiding |

| Author | Model | Therapist | Preparation | Integration |
|---|---|---|---|---|
| | | doctorate-level mental health Specialists) | | |
| von Rotz (2023) | Nondirective psychological support with no manual specified (psychological counselling) | Train therapists | Psychological safety and well-being, education, setting an intention, building trust, fostering openness and acceptance | Guidance on the integration of the experience including working through challenging emotions as well as facilitating the creation of a meaningful narrative thereof and support for adequate behavioural adaptations in everyday life |
| Raison (2023) | Nondirective psychological support with manual specified; Set and Setting [SaS] protocol; Usona Facilitator Training Manual and in the study manual of procedures | Clinical facilitators | To build rapport and therapeutic alliance for navigating the dosing session. | Three integration sessions with details provided in the Usona Facilitator Training Manual and in the study manual of procedures (MoP) |
| Holze (2023) | Nondirective psychological support, manualized | Not mentioned | Discussion about participant's life situation, beliefs, burdens, and resources, and the patient's wishes, hopes, and fears about the upcoming LSD session and how he or she deals with the illness for which they come to therapy | Helps to order, understand, deepen, and help answer "Why?," "What for?," and "What do I do with it?" questions. |
| Palhano-Fontes (2019) | Nondirective psychological support with no manual specified (asked to remain quiet, with their eyes closed, while focusing on their body, thoughts, and emotions) | Not mentioned | None | none |

| Author | Model | Therapist | Preparation | Integration |
|---|---|---|---|---|
| Mithoefer (2018) Ot'alora (2018) Wolfson (2020) Mitchell (2021) NCT01689740 | Nondirective psychological support, with MAPS (Manual for MDMA-Assisted Psychotherapy) manual | MAPStraining program | To gather participant history and to begin establishing an effective therapeutic alliance. To address the participant's questions and concerns, and gather information. | After each MDMA-assisted therapy session, participants engage in integrative sessions to discuss their experiences, address any emotions or thoughts that arise, and handle difficult reactions like anxiety or self-judgment. Multiple integrative sessions are scheduled before the next MDMA session or long-term follow-up. Additionally, there's a week of daily phone contact to assess well-being and determine if more therapist support is needed before the next scheduled visit. |

eTable 3. Network meta-regression of female proportion, mean age, baseline depression severity, disorder type, and follow-up assessment period

| Variable | Mean | 95% Credible interval | Rhat |
|---|---|---|---|
| Female proportion | 1.09 | -6.52 to 8.70 | 1 |
| Mean age | 0.01 | -0.40 to 0.43 | 1 |
| Baseline severity | 0.25 | -0.26 to 0.77 | 1 |
| Follow-up assessment period | 0.20 | -0.45 to 0.84 | 1 |
| Disorder type (vs MDD) | | | |
| Life-threatening illness | 1.82 | -2.93 to 6.55 | 1 |
| Posttraumatic distress disorder | 3.44 | -1.63 to 8.55 | 1 |

Abbreviation: MDD=major depressive disorder

*The Gelman and Rubin convergence diagnostic, or Rhat, approaching 1 indicates that multiple independent Markov Chain Monte Carlo (MCMC) chains have converged to similar distributions, suggesting that the simulation has reached a stable and reliable result.

eFigure 1. Study flow chart

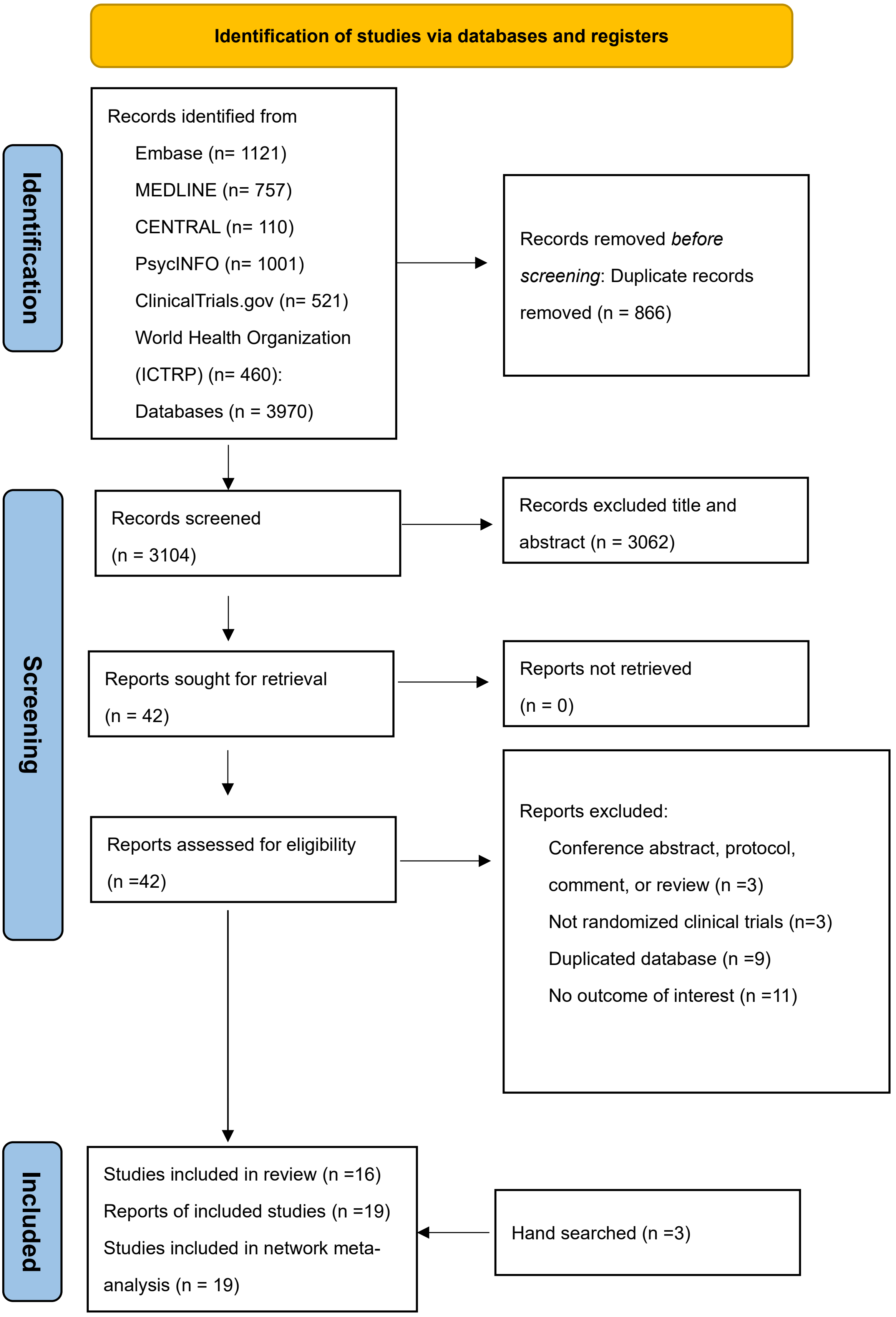


*From:* Page MJ, McKenzie JE, Bossuyt PM, Boutron I, Hoffmann TC, Mulrow CD, et al. The PRISMA 2020 statement: an updated guideline for reporting systematic reviews. BMJ 2021;372:n71. doi: 10.1136/bmj.n71

eFigure 2. Risk of bias plot of the included 19 studies A) psychedelic studies; B) non-psychedelic studies

**(A)**

Risk of bias domains

| Study | D1 | D2 | D3 | D4 | D5 | Overall |
|---|---|---|---|---|---|---|
| Carhart-Harris 2021 | + | - | + | + | + | + |
| Davis 2021 | - | + | + | + | + | + |
| Goodwin 2022 | + | + | + | + | + | + |
| Griffiths 2016 | + | + | - | - | + | - |
| Grob 2011 | + | - | + | - | + | - |
| Holze 2023 | - | - | + | + | + | - |
| Ross 2016 | + | + | + | - | + | + |
| Mitchell 2021 | + | + | + | + | + | + |
| Mithoefer 2018 | - | + | + | + | + | + |
| Ot'alora 2018 | + | + | + | + | + | + |
| Palhano-Fontes 2019 | - | + | + | - | + | - |
| Raison 2023 | + | + | + | + | + | + |
| von Rotz 2023 | + | + | + | + | + | + |
| Wolfson 2020 | + | - | + | + | + | + |
| NCT01689740 | - | - | - | - | - | - |

Domains:
D1: Bias arising from the randomization process.
D2: Bias due to deviations from intended intervention.
D3: Bias due to missing outcome data.
D4: Bias in measurement of the outcome.
D5: Bias in selection of the reported result.

Judgement
- Some concerns
+ Low

**(B)**

Risk of bias domains

| Study | D1 | D2 | D3 | D4 | D5 | Overall |
|---|---|---|---|---|---|---|
| Carhart-Harris 2021 | + | - | + | + | + | + |
| Burk 2002 | - | - | - | - | + | - |
| Hirayasu 2011a | - | - | - | - | + | - |
| Hirayasu 2011b | - | - | - | - | + | - |
| NCT00668525 | - | - | - | - | - | - |

Domains:
D1: Bias arising from the randomization process.
D2: Bias due to deviations from intended intervention.
D3: Bias due to missing outcome data.
D4: Bias in measurement of the outcome.
D5: Bias in selection of the reported result.

Judgement
- Some concerns
+ Low

eFigure 3. Summary of risk of bias on improvement of depressive symptoms across the 19 included studies. A) psychedelic studies; B) non-psychedelic studies

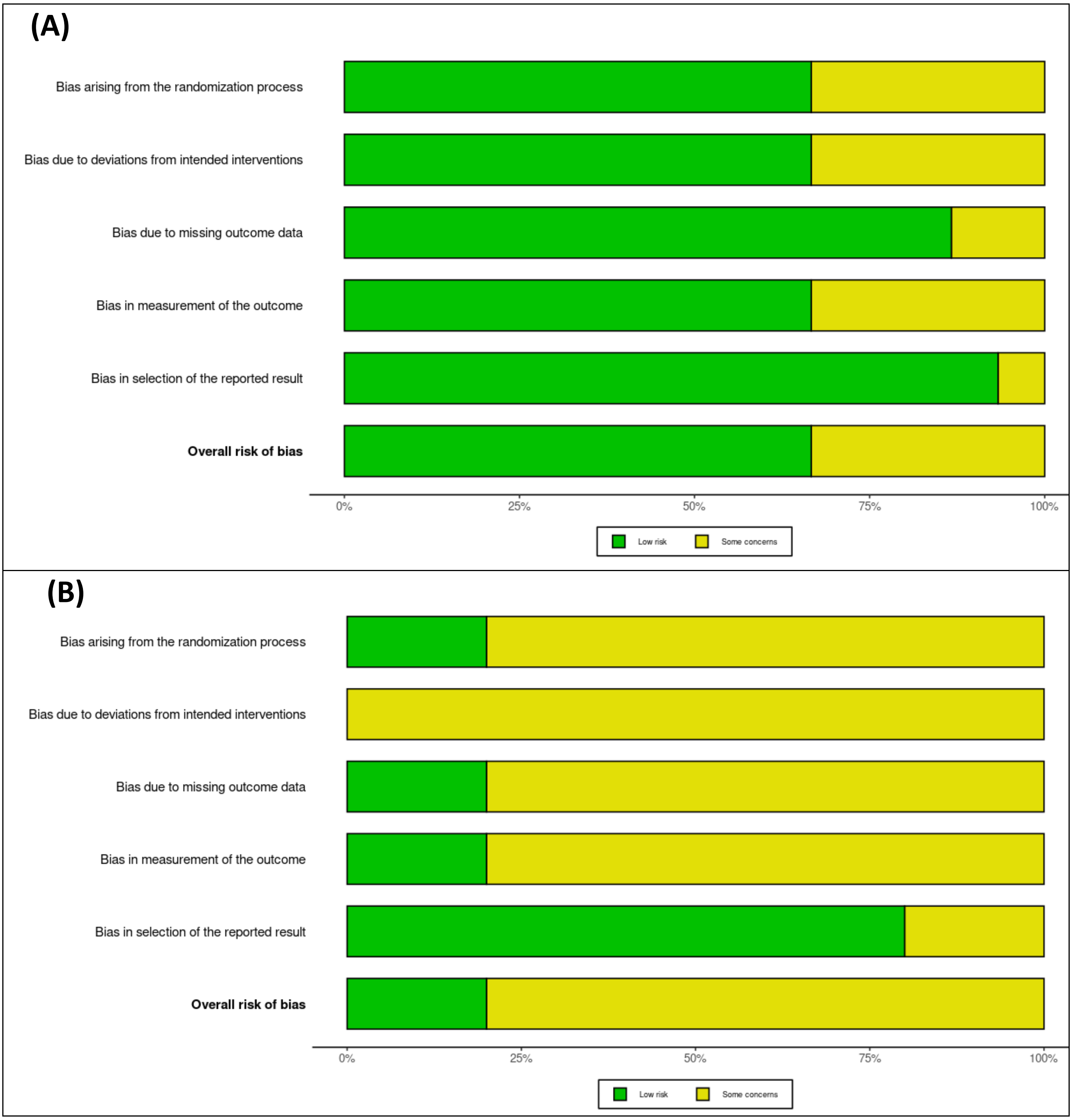

## eFigure 4. All comparative comparisons of all included interventions

| All comparions | | | | | | | | | | | | | | |
|---|---|---|---|---|---|---|---|---|---|---|---|---|---|---|
| .trtb | .trta | parameter | mean | sd | 2.5% | 97.5% | Bulk_ESS | Tail_ESS | Rhat | Hedges'g | Hse | LL | UL |
| Ayahuasca | PlaceboP | d[Ayahuasca vs. PlaceboP] | 9.16 | 2.42 | 4.24 | 13.78 | 89879.25 | 77915.73 | 1.00 | | | | |
| Escitalopram_10mg | PlaceboP | d[Escitalopram_10mg vs. PlaceboP] | 5.65 | 1.53 | 2.61 | 8.65 | 31045.59 | 53423.95 | 1.00 | | | | |
| Escitalopram_20mg | PlaceboP | d[Escitalopram_20mg vs. PlaceboP] | 5.62 | 1.45 | 2.74 | 8.44 | 30804.74 | 52813.25 | 1.00 | | | | |
| Extremely_low_MDMA | PlaceboP | d[Extremely_low_MDMA vs. PlaceboP] | 2.11 | 1.87 | -1.63 | 5.71 | 50673.59 | 71094.55 | 1.00 | | | | |
| Extremely_low_Psilocybin | PlaceboP | d[Extremely_low_Psilocybin vs. PlaceboP] | 3.96 | 1.67 | 0.61 | 7.17 | 63925.95 | 72402.53 | 1.00 | | | | |
| High_MDMA | PlaceboP | d[High_MDMA vs. PlaceboP] | 5.32 | 1.54 | 2.26 | 8.32 | 53655.97 | 74561.44 | 1.00 | | | | |
| High_Psilocybin | PlaceboP | d[High_Psilocybin vs. PlaceboP] | 10.31 | 1.13 | 7.96 | 12.44 | 43121.72 | 60515.63 | 1.00 | | | | |
| Low_MDMA | PlaceboP | d[Low_MDMA vs. PlaceboP] | 4.92 | 2.60 | -0.26 | 9.94 | 82922.69 | 81275.68 | 1.00 | | | | |
| Low_Psilocybin | PlaceboP | d[Low_Psilocybin vs. PlaceboP] | 5.95 | 1.53 | 2.83 | 8.87 | 71031.10 | 75728.80 | 1.00 | | | | |
| LSD | PlaceboP | d[LSD vs. PlaceboP] | 6.42 | 2.62 | 1.22 | 11.52 | 148639.24 | 82008.93 | 1.00 | | | | |
| PlaceboA | PlaceboP | d[PlaceboA vs. PlaceboP] | 3.79 | 1.54 | 0.77 | 6.80 | 31271.51 | 53913.20 | 1.00 | | | | |
| Escitalopram_10mg | Ayahuasca | d[Escitalopram_10mg vs. Ayahuasca] | -3.51 | 2.87 | -9.01 | 2.29 | 58129.58 | 70657.55 | 1.00 | | | | |
| Escitalopram_20mg | Ayahuasca | d[Escitalopram_20mg vs. Ayahuasca] | -3.55 | 2.82 | -8.96 | 2.14 | 60890.54 | 75352.68 | 1.00 | | | | |
| Extremely_low_MDMA | Ayahuasca | d[Extremely_low_MDMA vs. Ayahuasca] | -7.05 | 3.01 | -12.91 | -1.04 | 82174.42 | 83939.72 | 1.00 | | | | |
| Extremely_low_Psilocybin | Ayahuasca | d[Extremely_low_Psilocybin vs. Ayahuasca] | -5.20 | 2.90 | -10.80 | 0.60 | 94790.84 | 83278.35 | 1.00 | | | | |
| High_MDMA | Ayahuasca | d[High_MDMA vs. Ayahuasca] | -3.84 | 2.85 | -9.32 | 1.84 | 84748.59 | 87369.41 | 1.00 | | | | |
| High_Psilocybin | Ayahuasca | d[High_Psilocybin vs. Ayahuasca] | 1.15 | 2.61 | -3.92 | 6.35 | 93602.15 | 85750.23 | 1.00 | | | | |
| Low_MDMA | Ayahuasca | d[Low_MDMA vs. Ayahuasca] | -4.24 | 3.50 | -11.09 | 2.65 | 105884.33 | 93770.46 | 1.00 | | | | |
| Low_Psilocybin | Ayahuasca | d[Low_Psilocybin vs. Ayahuasca] | -3.22 | 2.81 | -8.67 | 2.39 | 103849.30 | 86172.54 | 1.00 | | | | |
| LSD | Ayahuasca | d[LSD vs. Ayahuasca] | -2.74 | 3.52 | -9.57 | 4.21 | 142635.42 | 95409.87 | 1.00 | | | | |
| PlaceboA | Ayahuasca | d[PlaceboA vs. Ayahuasca] | -5.37 | 2.87 | -10.89 | 0.48 | 58299.75 | 73069.74 | 1.00 | | | | |
| Escitalopram_20mg | Escitalopram_10mg | d[Escitalopram_20mg vs. Escitalopram_10mg] | -0.03 | 0.80 | -1.70 | 1.56 | 114911.92 | 74862.65 | 1.00 | | | | |
| Extremely_low_MDMA | Escitalopram_10mg | d[Extremely_low_MDMA vs. Escitalopram_10mg] | -3.53 | 2.42 | -8.34 | 1.17 | 39830.26 | 64725.44 | 1.00 | | | | |
| Extremely_low_Psilocybin | Escitalopram_10mg | d[Extremely_low_Psilocybin vs. Escitalopram_10mg] | -1.69 | 2.08 | -5.88 | 2.33 | 58160.14 | 70341.19 | 1.00 | | | | |
| High_MDMA | Escitalopram_10mg | d[High_MDMA vs. Escitalopram_10mg] | -0.33 | 2.18 | -4.62 | 3.93 | 38943.02 | 64742.96 | 1.00 | | | | |
| High_Psilocybin | Escitalopram_10mg | d[High_Psilocybin vs. Escitalopram_10mg] | 4.66 | 1.61 | 1.36 | 7.74 | 47913.82 | 62451.46 | 1.00 | 0.22 | 0.08 | 0.07 | 0.37 |
| Low_MDMA | Escitalopram_10mg | d[Low_MDMA vs. Escitalopram_10mg] | -0.73 | 3.02 | -6.72 | 5.16 | 56912.92 | 76227.91 | 1.00 | | | | |
| Low_Psilocybin | Escitalopram_10mg | d[Low_Psilocybin vs. Escitalopram_10mg] | 0.30 | 2.07 | -3.85 | 4.27 | 51280.40 | 69564.19 | 1.00 | | | | |
| LSD | Escitalopram_10mg | d[LSD vs. Escitalopram_10mg] | 0.78 | 3.04 | -5.26 | 6.69 | 76039.89 | 75425.60 | 1.00 | | | | |
| PlaceboA | Escitalopram_10mg | d[PlaceboA vs. Escitalopram_10mg] | -1.86 | 0.81 | -3.50 | -0.21 | 120540.24 | 79214.44 | 1.00 | | | | |
| Extremely_low_MDMA | Escitalopram_20mg | d[Extremely_low_MDMA vs. Escitalopram_20mg] | -3.50 | 2.36 | -8.16 | 1.09 | 40567.81 | 65594.00 | 1.00 | | | | |
| Extremely_low_Psilocybin | Escitalopram_20mg | d[Extremely_low_Psilocybin vs. Escitalopram_20mg] | -1.65 | 2.00 | -5.66 | 2.23 | 61889.07 | 75587.34 | 1.00 | | | | |
| High_MDMA | Escitalopram_20mg | d[High_MDMA vs. Escitalopram_20mg] | -0.29 | 2.12 | -4.47 | 3.84 | 39748.68 | 63530.80 | 1.00 | | | | |
| High_Psilocybin | Escitalopram_20mg | d[High_Psilocybin vs. Escitalopram_20mg] | 4.69 | 1.49 | 1.64 | 7.54 | 49506.94 | 66107.14 | 1.00 | 0.24 | 0.08 | 0.09 | 0.38 |
| Low_MDMA | Escitalopram_20mg | d[Low_MDMA vs. Escitalopram_20mg] | -0.70 | 2.97 | -6.57 | 5.11 | 59305.60 | 78549.35 | 1.00 | | | | |
| Low_Psilocybin | Escitalopram_20mg | d[Low_Psilocybin vs. Escitalopram_20mg] | 0.33 | 1.99 | -3.66 | 4.16 | 53533.10 | 70856.96 | 1.00 | | | | |
| LSD | Escitalopram_20mg | d[LSD vs. Escitalopram_20mg] | 0.81 | 2.99 | -5.12 | 6.62 | 81240.17 | 82628.87 | 1.00 | | | | |
| PlaceboA | Escitalopram_20mg | d[PlaceboA vs. Escitalopram_20mg] | -1.82 | 0.81 | -3.43 | -0.16 | 109298.60 | 78662.58 | 1.00 | | | | |
| Extremely_low_Psilocybin | Extremely_low_MDMA | d[Extremely_low_Psilocybin vs. Extremely_low_MDMA] | 1.85 | 2.48 | -3.02 | 6.76 | 60972.33 | 80237.98 | 1.00 | | | | |
| High_MDMA | Extremely_low_MDMA | d[High_MDMA vs. Extremely_low_MDMA] | 3.21 | 1.49 | 0.34 | 6.22 | 121036.61 | 90332.00 | 1.00 | | | | |
| High_Psilocybin | Extremely_low_MDMA | d[High_Psilocybin vs. Extremely_low_MDMA] | 8.19 | 2.14 | 3.99 | 12.41 | 54764.63 | 76394.55 | 1.00 | | | | |
| Low_MDMA | Extremely_low_MDMA | d[Low_MDMA vs. Extremely_low_MDMA] | 2.81 | 2.45 | -2.01 | 7.63 | 187042.02 | 97734.78 | 1.00 | | | | |
| Low_Psilocybin | Extremely_low_MDMA | d[Low_Psilocybin vs. Extremely_low_MDMA] | 3.83 | 2.38 | -0.84 | 8.51 | 64478.18 | 81602.78 | 1.00 | | | | |
| LSD | Extremely_low_MDMA | d[LSD vs. Extremely_low_MDMA] | 4.31 | 3.20 | -1.99 | 10.58 | 101320.89 | 90416.76 | 1.00 | | | | |
| PlaceboA | Extremely_low_MDMA | d[PlaceboA vs. Extremely_low_MDMA] | 1.68 | 2.42 | -3.02 | 6.49 | 39830.22 | 62256.17 | 1.00 | | | | |
| High_MDMA | Extremely_low_Psilocybin | d[High_MDMA vs. Extremely_low_Psilocybin] | 1.36 | 2.26 | -3.08 | 5.84 | 62595.42 | 78106.66 | 1.00 | | | | |
| High_Psilocybin | Extremely_low_Psilocybin | d[High_Psilocybin vs. Extremely_low_Psilocybin] | 6.35 | 1.48 | 3.41 | 9.21 | 129499.02 | 95103.58 | 1.00 | 0.51 | 0.12 | 0.27 | 0.74 |
| Low_MDMA | Extremely_low_Psilocybin | d[Low_MDMA vs. Extremely_low_Psilocybin] | 0.96 | 3.07 | -5.11 | 6.96 | 84665.54 | 89753.42 | 1.00 | | | | |
| Low_Psilocybin | Extremely_low_Psilocybin | d[Low_Psilocybin vs. Extremely_low_Psilocybin] | 1.98 | 1.87 | -1.71 | 5.65 | 127889.25 | 94340.41 | 1.00 | | | | |
| LSD | Extremely_low_Psilocybin | d[LSD vs. Extremely_low_Psilocybin] | 2.46 | 3.08 | -3.61 | 8.48 | 119287.04 | 83356.77 | 1.00 | | | | |
| PlaceboA | Extremely_low_Psilocybin | d[PlaceboA vs. Extremely_low_Psilocybin] | -0.17 | 2.08 | -4.22 | 4.01 | 58562.58 | 69681.08 | 1.00 | | | | |
| High_Psilocybin | High_MDMA | d[High_Psilocybin vs. High_MDMA] | 4.98 | 1.89 | 1.23 | 8.67 | 54326.91 | 73886.86 | 1.00 | 0.32 | 0.12 | 0.08 | 0.55 |
| Low_MDMA | High_MDMA | d[Low_MDMA vs. High_MDMA] | -0.40 | 2.43 | -5.21 | 4.31 | 174401.14 | 93180.05 | 1.00 | | | | |
| Low_Psilocybin | High_MDMA | d[Low_Psilocybin vs. High_MDMA] | 0.62 | 2.16 | -3.65 | 4.83 | 67036.20 | 85009.30 | 1.00 | | | | |
| LSD | High_MDMA | d[LSD vs. High_MDMA] | 1.10 | 3.03 | -4.88 | 7.06 | 111616.16 | 90852.28 | 1.00 | | | | |
| PlaceboA | High_MDMA | d[PlaceboA vs. High_MDMA] | -1.53 | 2.18 | -5.78 | 2.79 | 39020.20 | 64608.34 | 1.00 | | | | |
| Low_MDMA | High_Psilocybin | d[Low_MDMA vs. High_Psilocybin] | -5.39 | 2.79 | -10.89 | 0.08 | 84304.65 | 87789.36 | 1.00 | | | | |
| Low_Psilocybin | High_Psilocybin | d[Low_Psilocybin vs. High_Psilocybin] | -4.36 | 1.60 | -7.51 | -1.20 | 114251.88 | 95184.55 | 1.00 | 0.32 | 0.12 | 0.09 | 0.56 |
| LSD | High_Psilocybin | d[LSD vs. High_Psilocybin] | -3.88 | 2.82 | -9.44 | 1.66 | 128877.01 | 85614.86 | 1.00 | | | | |
| PlaceboA | High_Psilocybin | d[PlaceboA vs. High_Psilocybin] | -6.52 | 1.61 | -9.57 | -3.19 | 48087.26 | 60956.80 | 1.00 | | | | |
| Low_Psilocybin | Low_MDMA | d[Low_Psilocybin vs. Low_MDMA] | 1.03 | 2.99 | -4.80 | 6.93 | 92491.73 | 88635.17 | 1.00 | | | | |
| LSD | Low_MDMA | d[LSD vs. Low_MDMA] | 1.50 | 3.66 | -5.64 | 8.73 | 123012.23 | 93296.22 | 1.00 | | | | |
| PlaceboA | Low_MDMA | d[PlaceboA vs. Low_MDMA] | -1.13 | 3.02 | -7.00 | 4.85 | 57070.67 | 77415.79 | 1.00 | | | | |
| LSD | Low_Psilocybin | d[LSD vs. Low_Psilocybin] | 0.48 | 3.02 | -5.51 | 6.37 | 139937.95 | 86454.75 | 1.00 | | | | |
| PlaceboA | Low_Psilocybin | d[PlaceboA vs. Low_Psilocybin] | -2.15 | 2.07 | -6.13 | 2.01 | 51848.08 | 68535.26 | 1.00 | | | | |
| PlaceboA | LSD | d[PlaceboA vs. LSD] | -2.63 | 3.04 | -8.53 | 3.41 | 76974.18 | 79212.71 | 1.00 | | | | |

PlaceboP indicates the placebo response observed in psychedelic trials, and PlaceboA indicates the placebo response observed in antidepressant (escitalopram) trials. We defined high, low, and extremely low doses of the included psychedelics as follows: (i) psilocybin: high-dose, ≥20 mg; extremely-low-dose, 1-3 mg; low-dose, other range; and (ii) MDMA: high-dose, ≥100 mg; extremely-low-dose, ≤40 mg; low-dose, other range. Escitalopram was divided into escitalopram_10mg and escitalopram_20mg (≥20 mg).

Abbreviations: Hse: standard error of Hedges’ g; LL: lower limit of 95% credible interval; LSD, lysergic acid diethylamide; MDMA; 3,4-methylenedioxymethamphetamine; UL: upper limit of 95% credible interval.

eFigure 5. Transitivity assumption of study characteristics by direct treatment comparisons

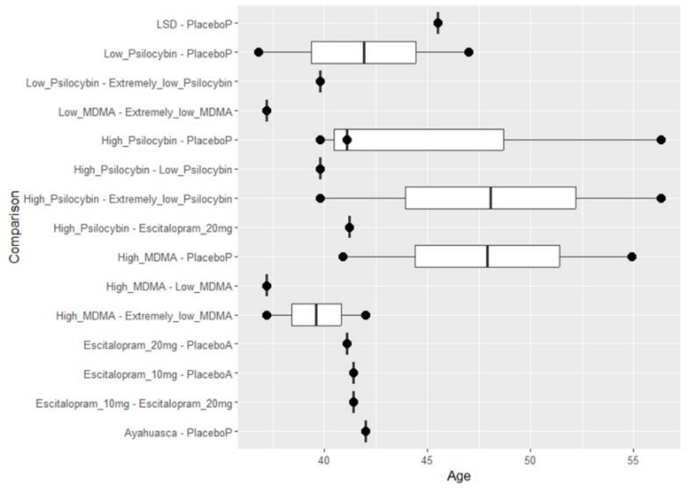


We defined high, low, and extremely low doses of the included psychedelics as follows: (i) psilocybin: high-dose, ≥20 mg; extremely-low-dose, 1-3 mg; low-dose, other range; and (ii) MDMA: high-dose, ≥100 mg; extremely-low-dose, ≤40 mg; low-dose, other range. Escitalopram was divided into escitalopram_10mg and escitalopram_20mg (≥20 mg).

PlaceboP indicates the placebo response observed in psychedelic trials, and PlaceboA indicates the placebo response observed in antidepressant (escitalopram) trials.

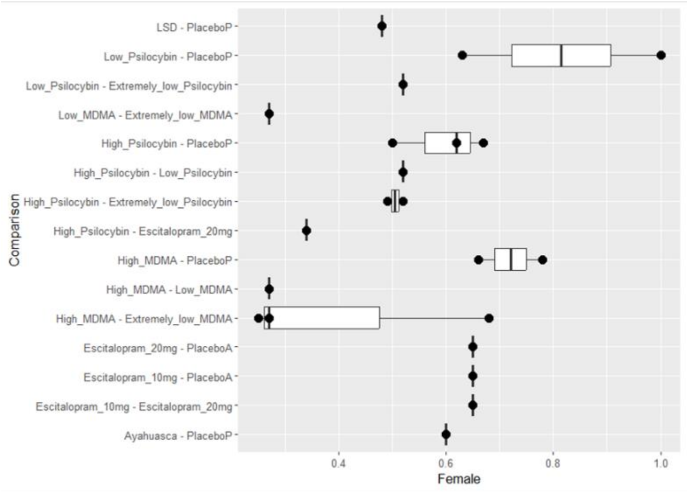


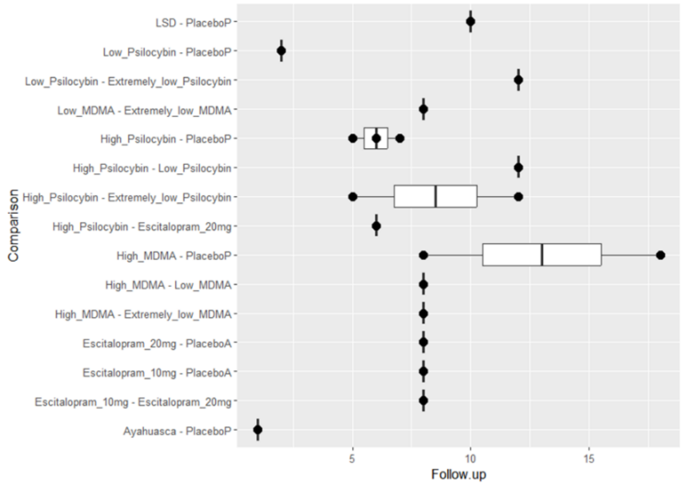


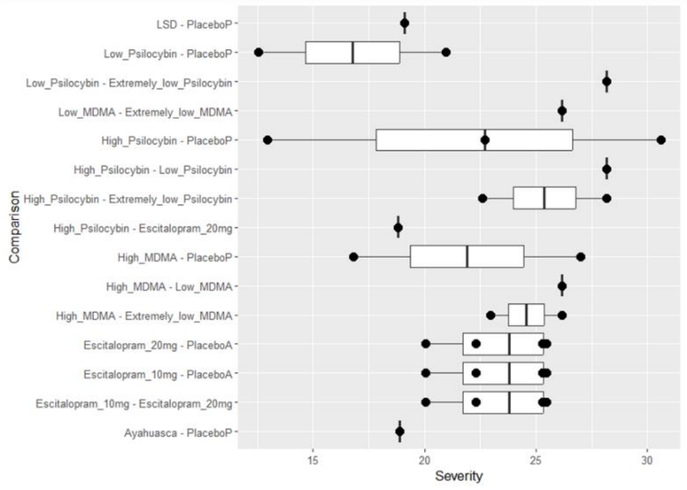


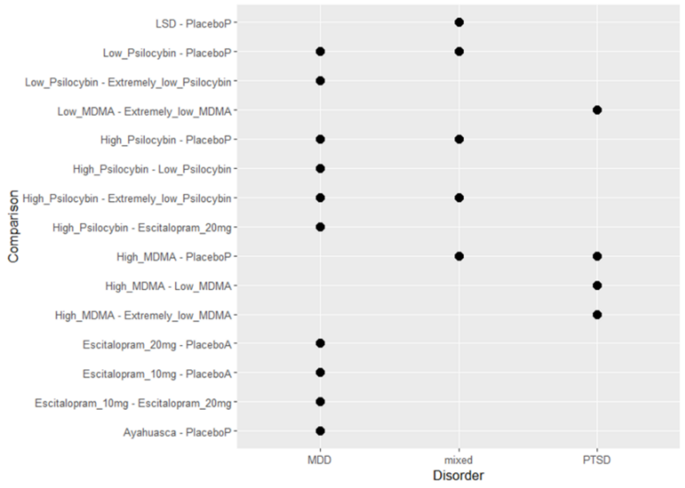


Most of the comparisons had similar mean age, female proportion, and baseline severity but there were a few comparisons which had relatively low or high female proportions. Network meta-regression of these three variables, however, did not show that they impacted on the network estimates, although results from such analyses might suffer from potential ecological bias.

eFigure 6. Transitivity assumption of escitalopram effectiveness between two study designs.

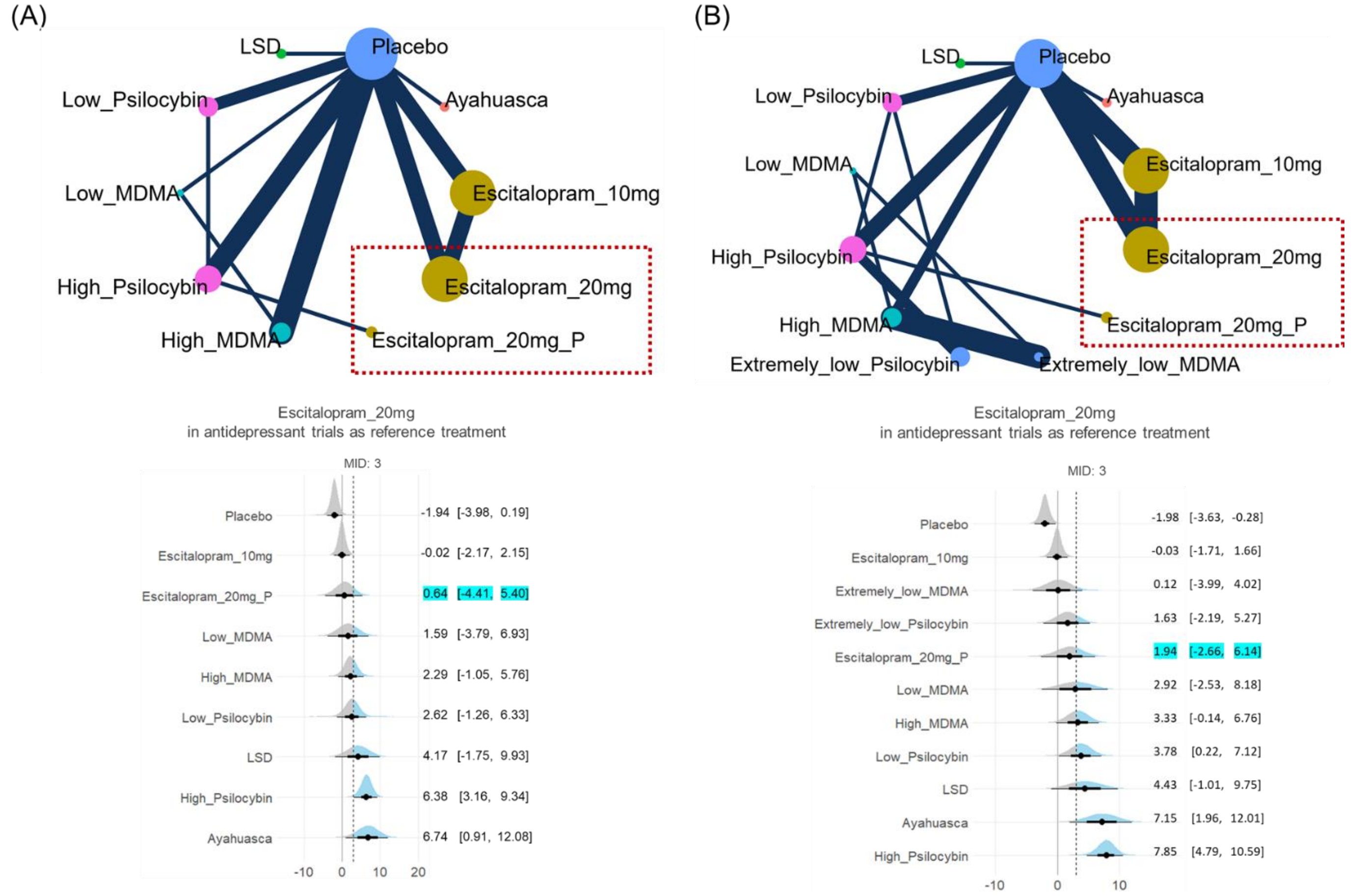


[a] In left network structure, all controlled arms were grouped as a common comparator. In the right network structure, extremely-low-dose psychedelics were considered active interventions. The effectiveness of escitalopram 20mg used in the head-to-head trial of psilocybin vs escitalopram (denoted as Escitalopram_20mg_P) did not differ significantly from that used in the antidepressant trials.

Abbreviations: LSD, lysergic acid diethylamide; MDMA; 3,4-methylenedioxymethamphetamine; NMA, network meta-analysis.

eFigure 7. All comparative comparisons of all included interventions in patients with major depressive disorder

| .trtb | .trta | mean | 2.5% | 97.5% | Bulk_ESS | Tail_ESS | Rhat | sd |
|---|---|---|---|---|---|---|---|---|
| Ayahuasca | PlaceboP | 9.38 | 4.85 | 13.79 | 120724.31 | 84069.69 | 1.00 | 2.28 |
| Escitalopram_10mg | PlaceboP | 5.86 | 2.99 | 8.73 | 29624.04 | 52062.63 | 1.00 | 1.46 |
| Escitalopram_20mg | PlaceboP | 5.85 | 3.07 | 8.59 | 29200.95 | 50776.04 | 1.00 | 1.41 |
| Extremely_low_Psilocybin | PlaceboP | 6.46 | 2.32 | 10.41 | 76984.69 | 76509.87 | 1.00 | 2.06 |
| High_Psilocybin | PlaceboP | 10.82 | 8.46 | 13.01 | 47808.98 | 64156.06 | 1.00 | 1.16 |
| Low_Psilocybin | PlaceboP | 7.00 | 3.78 | 10.05 | 71535.17 | 71931.04 | 1.00 | 1.59 |
| PlaceboA | PlaceboP | 4.00 | 1.13 | 6.87 | 29691.91 | 50873.22 | 1.00 | 1.46 |
| Escitalopram_10mg | Ayahuasca | -3.53 | -8.77 | 1.84 | 65394.22 | 78104.06 | 1.00 | 2.70 |
| Escitalopram_20mg | Ayahuasca | -3.53 | -8.69 | 1.74 | 67784.30 | 80211.41 | 1.00 | 2.66 |
| Extremely_low_Psilocybin | Ayahuasca | -2.92 | -8.88 | 3.00 | 112096.79 | 92023.06 | 1.00 | 3.02 |
| High_Psilocybin | Ayahuasca | 1.44 | -3.52 | 6.40 | 105231.73 | 92800.60 | 1.00 | 2.52 |
| Low_Psilocybin | Ayahuasca | -2.39 | -7.73 | 3.01 | 113735.98 | 95567.86 | 1.00 | 2.74 |
| PlaceboA | Ayahuasca | -5.38 | -10.61 | -0.02 | 65654.81 | 78369.10 | 1.00 | 2.70 |
| Escitalopram_20mg | Escitalopram_10mg | 0.00 | -1.37 | 1.31 | 126090.51 | 86265.88 | 1.00 | 0.67 |
| Extremely_low_Psilocybin | Escitalopram_10mg | 0.60 | -4.13 | 5.17 | 70798.26 | 73387.87 | 1.00 | 2.36 |
| High_Psilocybin | Escitalopram_10mg | 4.96 | 1.97 | 7.82 | 57796.90 | 67676.36 | 1.00 | 1.48 |
| Low_Psilocybin | Escitalopram_10mg | 1.14 | -2.93 | 5.03 | 56073.30 | 70384.19 | 1.00 | 2.04 |
| PlaceboA | Escitalopram_10mg | -1.86 | -3.19 | -0.52 | 130347.70 | 93662.03 | 1.00 | 0.66 |
| Extremely_low_Psilocybin | Escitalopram_20mg | 0.61 | -4.02 | 5.09 | 74418.21 | 76933.96 | 1.00 | 2.31 |
| High_Psilocybin | Escitalopram_20mg | 4.97 | 2.19 | 7.64 | 59842.18 | 71558.73 | 1.00 | 1.39 |
| Low_Psilocybin | Escitalopram_20mg | 1.14 | -2.78 | 4.99 | 58594.55 | 75415.72 | 1.00 | 1.98 |
| PlaceboA | Escitalopram_20mg | -1.85 | -3.18 | -0.48 | 123310.43 | 89767.24 | 1.00 | 0.67 |
| High_Psilocybin | Extremely_low_Psilocybin | 4.36 | 0.54 | 8.27 | 139941.67 | 93822.43 | 1.00 | 1.97 |
| Low_Psilocybin | Extremely_low_Psilocybin | 0.54 | -3.46 | 4.58 | 175388.21 | 95044.63 | 1.00 | 2.05 |
| PlaceboA | Extremely_low_Psilocybin | -2.46 | -7.04 | 2.26 | 71069.15 | 74039.39 | 1.00 | 2.36 |
| Low_Psilocybin | High_Psilocybin | -3.82 | -7.04 | -0.61 | 118437.13 | 93456.70 | 1.00 | 1.64 |
| PlaceboA | High_Psilocybin | -6.82 | -9.67 | -3.84 | 58069.47 | 69120.49 | 1.00 | 1.48 |
| PlaceboA | Low_Psilocybin | -3.00 | -6.91 | 1.07 | 56516.85 | 71287.19 | 1.00 | 2.04 |

PlaceboP indicates the placebo response observed in psychedelic trials, and PlaceboA indicates the placebo response observed in antidepressant (escitalopram) trials. We defined high, low, and extremely low doses of the included psychedelics as follows: (i) psilocybin: high-dose, ≥20 mg; extremely-low-dose, 1-3 mg; low-dose, other range; and (ii) MDMA: high-dose, ≥100 mg; extremely-low-dose, ≤40 mg; low-dose, other range. Escitalopram was divided into escitalopram_10mg and escitalopram_20mg (≥20 mg).

eFigure 8. Sensitivity analysis 2: excluding studies with high risk of bias

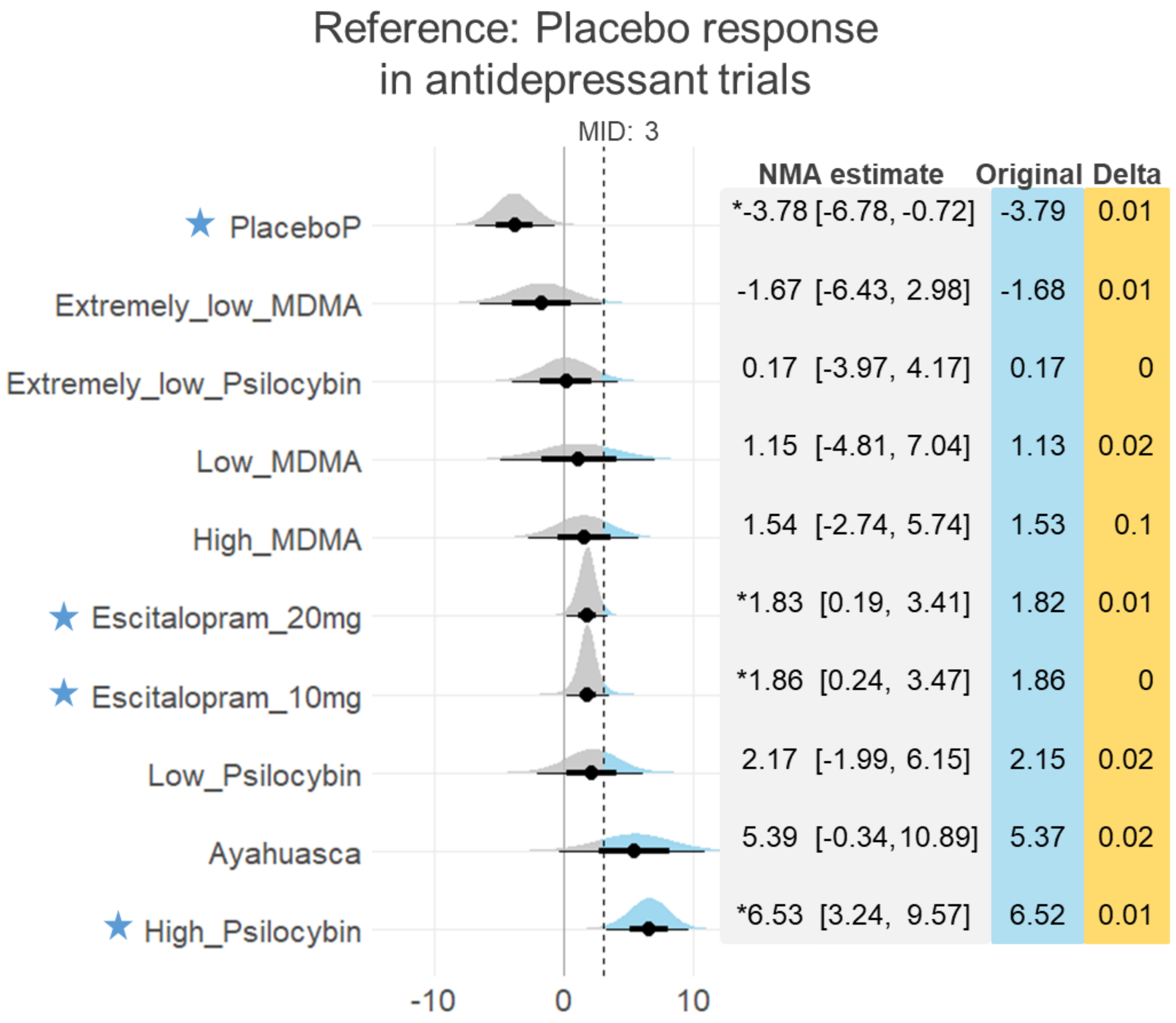


PlaceboP indicates the placebo response observed in psychedelic trials, and PlaceboA indicates the placebo response observed in antidepressant (escitalopram) trials. We defined high, low, and extremely low doses of the included psychedelics as follows: (i) psilocybin: high-dose, ≥20 mg; extremely-low-dose, 1-3 mg; low-dose, other range; and (ii) MDMA: high-dose, ≥100 mg; extremely-low-dose, ≤40 mg; low-dose, other range. Escitalopram was divided into escitalopram_10mg and escitalopram_20mg (≥20 mg).

Abbreviations: MDMA; 3,4-methylenedioxymethamphetamine; MID: minimal important difference of 17-item Hamilton Depression Rating Scale; NMA, network meta-analysis.

eFigure 9. Sensitivity analysis 3: adjusting for baseline depression severity

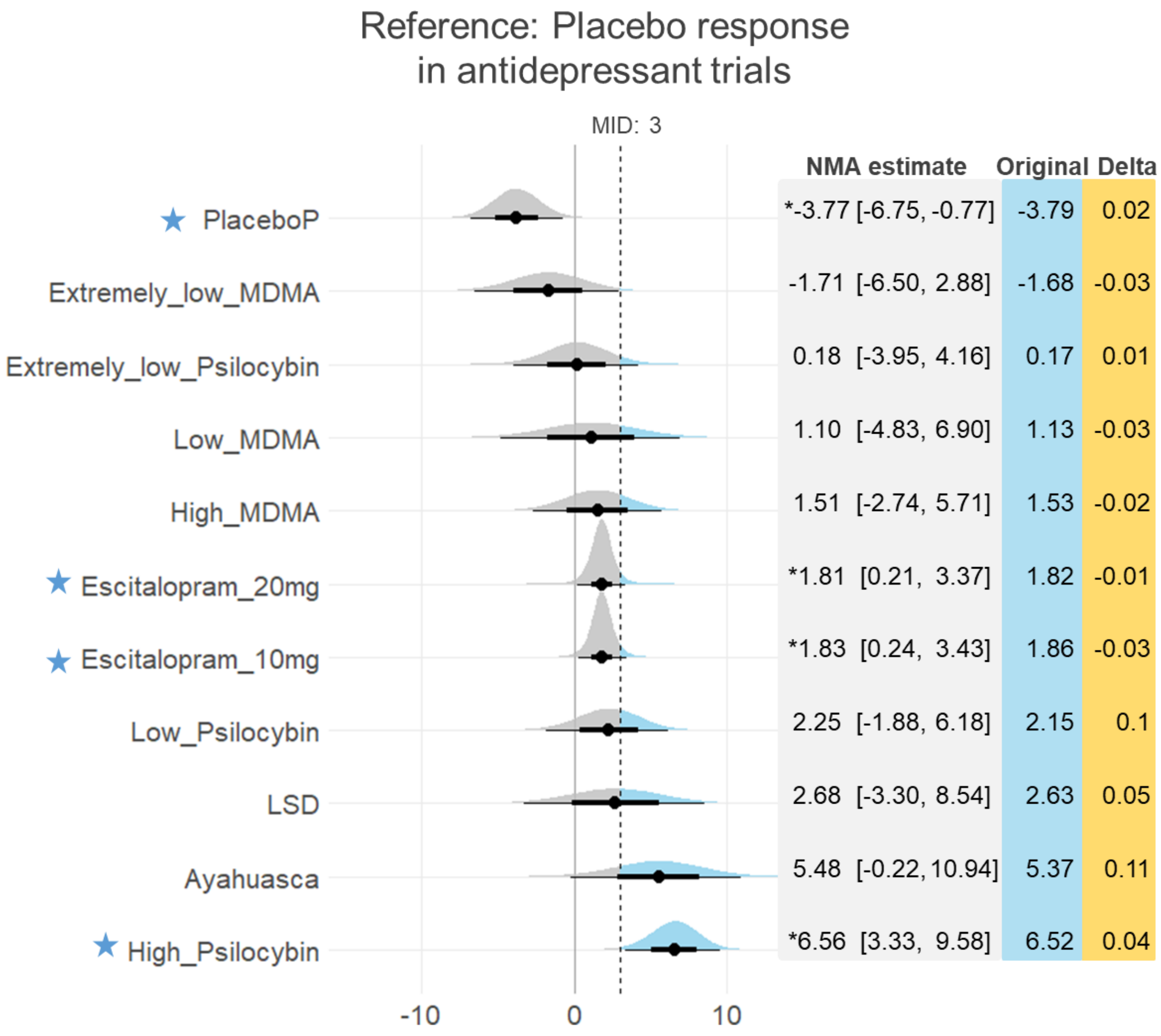


PlaceboP indicates the placebo response observed in psychedelic trials, and PlaceboA indicates the placebo response observed in antidepressant (escitalopram) trials. We defined high, low, and extremely low doses of the included psychedelics as follows: (i) psilocybin: high-dose, ≥20 mg; extremely-low-dose, 1-3 mg; low-dose, other range; and (ii) MDMA: high-dose, ≥100 mg; extremely-low-dose, ≤40 mg; low-dose, other range. Escitalopram was divided into escitalopram_10mg and escitalopram_20mg (≥20 mg).

Abbreviations: LSD, lysergic acid diethylamide; MDMA; 3,4-methylenedioxymethamphetamine; MID: minimal important difference of 17-item Hamilton Depression Rating Scale; NMA, network meta-analysis.

eFigure 10. Sensitivity analysis 4: using most conservative correlation coefficient of zero

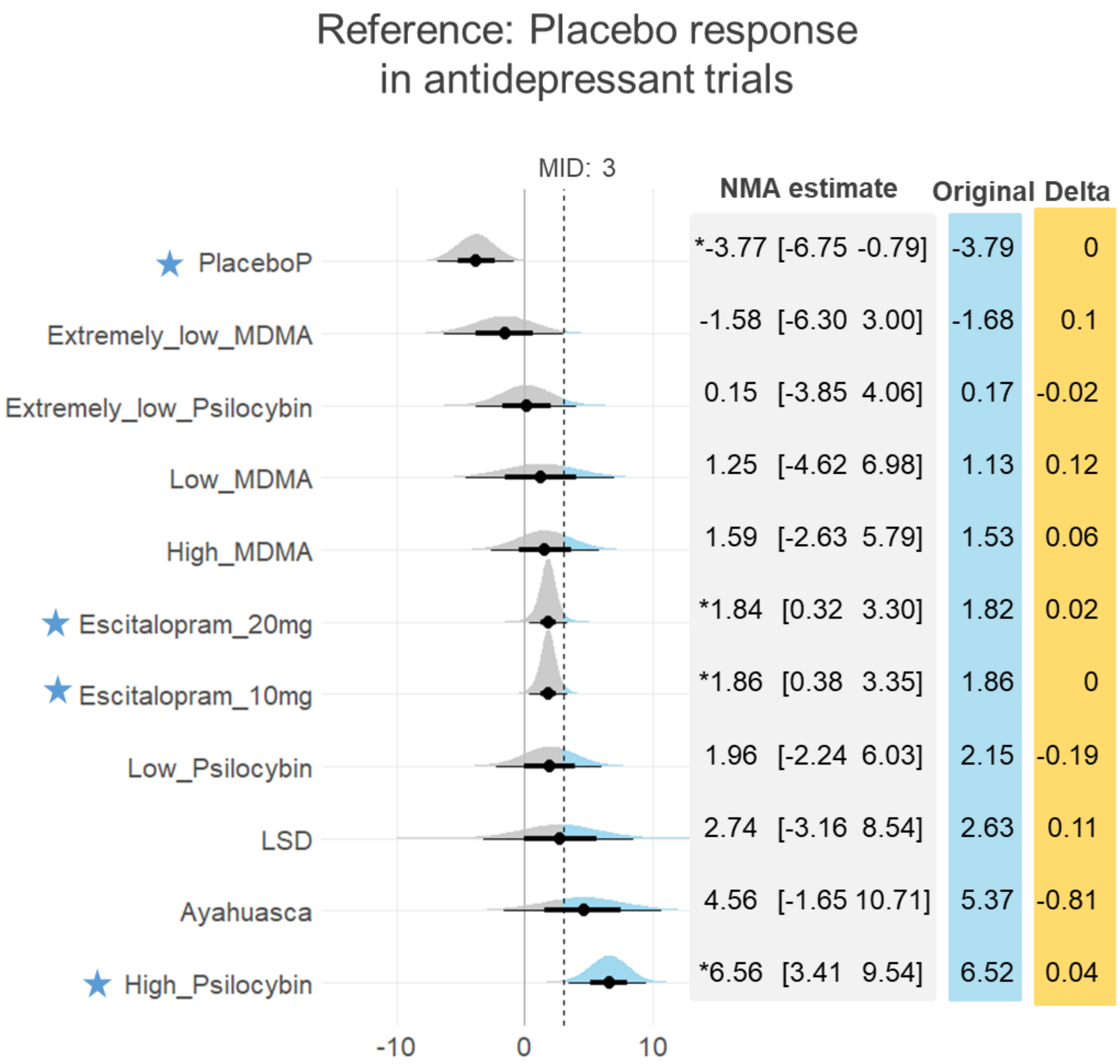


PlaceboP indicates the placebo response observed in psychedelic trials, and PlaceboA indicates the placebo response observed in antidepressant (escitalopram) trials. We defined high, low, and extremely low doses of the included psychedelics as follows: (i) psilocybin: high-dose, ≥20 mg; extremely-low-dose, 1-3 mg; low-dose, other range; and (ii) MDMA: high-dose, ≥100 mg; extremely-low-dose, ≤40 mg; low-dose, other range. Escitalopram was divided into escitalopram_10mg and escitalopram_20mg (≥20 mg).

Abbreviations: LSD, lysergic acid diethylamide; MDMA; 3,4-methylenedioxymethamphetamine; MID: minimal important difference of 17-item Hamilton Depression Rating Scale; NMA, network meta-analysis.

eFigure 11. Forest plots of NMA estimates of all-cause discontinuation and severe adverse event

(A) All-cause discontinuation (B) Severe adverse event

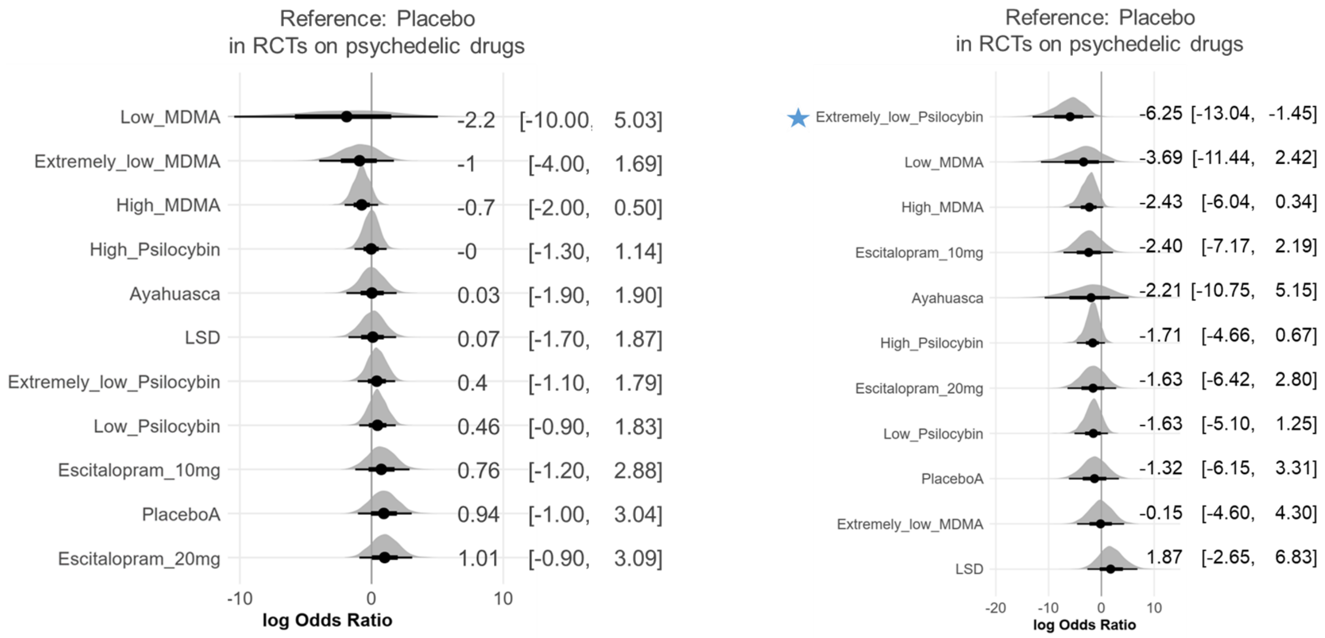


PlaceboP indicates the placebo response observed in psychedelic trials, and PlaceboA indicates the placebo response observed in antidepressant (escitalopram) trials. We defined high, low, and extremely low doses of the included psychedelics as follows: (i) psilocybin: high-dose, ≥20 mg; extremely-low-dose, 1-3 mg; low-dose, other range; and (ii) MDMA: high-dose, ≥100 mg; extremely-low-dose, ≤40 mg; low-dose, other range. Escitalopram was divided into escitalopram_10mg and escitalopram_20mg (≥20 mg).

Abbreviations: LSD, lysergic acid diethylamide; MDMA; 3,4-methylenedioxymethamphetamine.

eFigure 12. Funnel plot (order by placebo used in psychedelic trials)

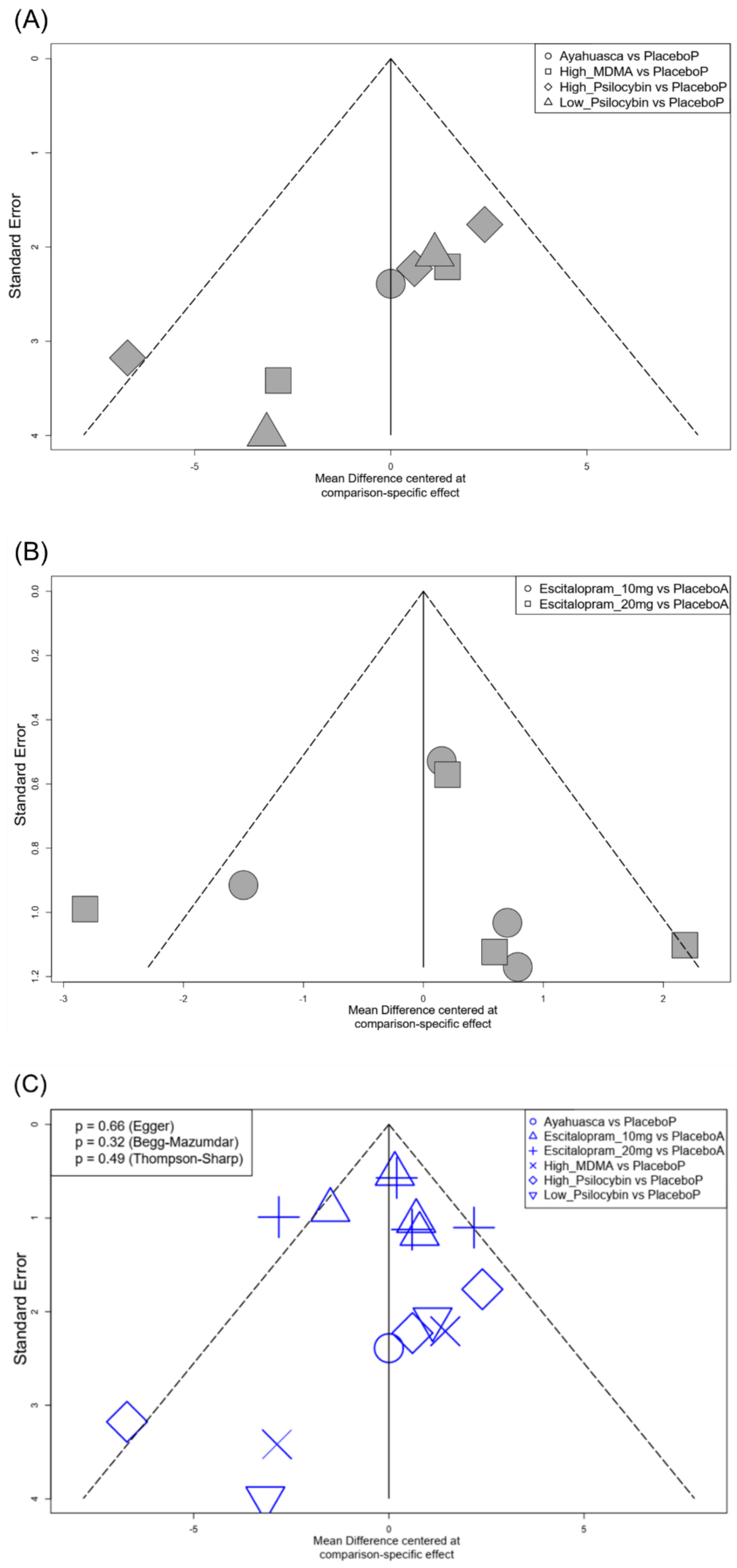


eFigure 12A: PlaceboP as the comparator; eFigure 12B: PlaceboA as the comparator; eFigure 12C: both PlaceboP and PlaceboA as the comparators

# eFigure 13. GRADE assessment for the primary outcome

| Treatment 1 | Treatment 2 | k | Direct estimate | RoB | Inc | Ind | Pub | Rating | Indirect estimate | Initial certainty | Intransitivity | Rating | NMA estimate | Initial certainty | Incoherence | Imprecision | Final Rating |
|---|---|---|---|---|---|---|---|---|---|---|---|---|---|---|---|---|---|
| High dose psilocybin | PlaceboP | 3 | 10.45 (7.50, 13.22) | 0 | 0 | 0 | 0 | High | 10.06 (6.78, 13.10) | High | 0 | High | 10.3 (7.94, 12.44) | High | 0 | -1 | Moderate |
| Low dose psilocybin | PlaceboP | 2 | 7.05 (3.13, 10.79) | 0 | 0 | 0 | 0 | High | 4.69 (0.60, 8.63) | High | 0 | High | 5.94 (2.82, 8.87) | High | 0 | -1 | Moderate |
| Extremely low dose Psilocybin | PlaceboP | 0 | NA | NA | NA | NA | NA | NA | 3.96 (0.61, 7.17) | High | 0 | High | 3.96 (0.61, 7.17) | High | 0 | -1 | Moderate |
| LSD | PlaceboP | 1 | 6.42 (1.22, 11.25) | 0 | 0 | 0 | 0 | High | NA | NA | NA | NA | 6.42 (1.22, 11.25) | High | 0 | -1 | Moderate |
| Ayahuasca | PlaceboP | 1 | 9.16 (4.24, 13.78) | 0 | 0 | 0 | 0 | High | NA | NA | NA | NA | 9.16 (4.24, 13.78) | High | 0 | -1 | Moderate |
| High dose MDMA | PlaceboP | 2 | 5.32 (2.26, 8.32) | 0 | 0 | 0 | 0 | High | NA | NA | NA | NA | 5.32 (2.26, 8.32) | High | 0 | -1 | Moderate |
| Low dose MDMA | PlaceboP | 0 | NA | NA | NA | NA | NA | NA | 4.92 (-0.26, 9.94) | High | 0 | High | 4.92 (-0.26, 9.94) | High | 0 | -2 | Low |
| Extremely low dose MDMA | PlaceboP | 0 | NA | NA | NA | NA | NA | NA | 2.11 (-1.63, 5.71) | High | 0 | High | 2.11 (-1.63, 5.71) | High | 0 | -2 | Low |
| Escitalopram 20mg | PlaceboP | 0 | NA | NA | NA | NA | NA | NA | 5.62 (2.74, 8.44) | High | 0 | High | 5.62 (2.74, 8.44) | High | 0 | -1 | Moderate |
| Escitalopram 10mg | PlaceboP | 0 | NA | NA | NA | NA | NA | NA | 5.65 (2.61, 8.65) | Moderate | 0 | Moderate | 5.65 (2.61, 8.65) | Moderate | 0 | -1 | Low |
| PlaceboA | PlaceboP | 0 | NA | NA | NA | NA | NA | NA | 3.79 (0.77, 6.80) | High | 0 | High | 3.79 (0.77, 6.80) | High | 0 | -1 | Moderate |
| High dose psilocybin | PlaceboA | 0 | NA | NA | NA | NA | NA | NA | 6.52 (3.19, 9.57) | High | 0 | High | 6.52 (3.19, 9.57) | High | 0 | 0 | High |
| Low dose psilocybin | PlaceboA | 0 | NA | NA | NA | NA | NA | NA | 2.15 (-2.01, 6.13) | High | 0 | High | 2.15 (-2.01, 6.13) | High | 0 | -2 | Low |
| Extremely low dose Psilocybin | PlaceboA | 0 | NA | NA | NA | NA | NA | NA | 0.17 (-4.01, 4.22) | High | 0 | High | 0.17 (-4.01, 4.22) | High | 0 | -2 | Low |
| LSD | PlaceboA | 0 | NA | NA | NA | NA | NA | NA | 2.63 (-3.41, 8.53) | High | 0 | High | 2.63 (-3.41, 8.53) | High | 0 | -2 | Low |
| Ayahuasca | PlaceboA | 0 | NA | NA | NA | NA | NA | NA | 5.37 (-0.48, 10.89) | High | 0 | High | 5.37 (-0.48, 10.89) | High | 0 | -2 | Low |
| High dose MDMA | PlaceboA | 0 | NA | NA | NA | NA | NA | NA | 1.53 (-2.79, 5.78) | High | 0 | High | 1.53 (-2.79, 5.78) | High | 0 | -2 | Low |
| Low dose MDMA | PlaceboA | 0 | NA | NA | NA | NA | NA | NA | 1.13 (-4.85, 7.00) | High | 0 | High | 1.13 (-4.85, 7.00) | High | 0 | -2 | Low |
| Extremely low dose MDMA | PlaceboA | 0 | NA | NA | NA | NA | NA | NA | -1.68 (-6.49, 3.02) | High | 0 | High | -1.68 (-6.49, 3.02) | High | 0 | -2 | Low |
| Escitalopram 20mg | PlaceboA | 4 | 1.82 (0.16, 3.43) | 0 | 0 | 0 | 0 | High | NA | NA | NA | NA | 1.82 (0.16, 3.43) | High | 0 | -1 | Moderate |
| Escitalopram 10mg | PlaceboA | 4 | 1.86 (0.21, 3.50) | 0 | 0 | 0 | 0 | High | NA | NA | NA | NA | 1.86 (0.21, 3.50) | High | 0 | -1 | Moderate |
| High dose psilocybin | Escitalopram 10mg | 0 | NA | NA | NA | NA | NA | NA | 4.66 (1.36, 7.74) | Moderate | 0 | Moderate | 4.66 (1.36, 7.74) | Moderate | 0 | -1 | Low |
| Low dose psilocybin | Escitalopram 10mg | 0 | NA | NA | NA | NA | NA | NA | 0.30 (-3.85, 4.27) | Moderate | 0 | Moderate | 0.30 (-3.85, 4.27) | Moderate | 0 | -2 | Very Low |
| Extremely low dose Psilocybin | Escitalopram 10mg | 0 | NA | NA | NA | NA | NA | NA | -1.69 (-5.88, 2.33) | Moderate | 0 | Moderate | -1.69 (-5.88, 2.33) | Moderate | 0 | -2 | Very Low |
| LSD | Escitalopram 10mg | 0 | NA | NA | NA | NA | NA | NA | 0.78 (-5.26, 6.69) | Moderate | 0 | Moderate | 0.78 (-5.26, 6.69) | Moderate | 0 | -2 | Very Low |
| Ayahuasca | Escitalopram 10mg | 0 | NA | NA | NA | NA | NA | NA | 3.51 (-2.29, 9.01) | Moderate | 0 | Moderate | 3.51 (-2.29, 9.01) | Moderate | 0 | -2 | Very Low |
| High dose MDMA | Escitalopram 10mg | 0 | NA | NA | NA | NA | NA | NA | -0.33 (-4.62, 3.93) | Moderate | 0 | Moderate | -0.33 (-4.62, 3.93) | Moderate | 0 | -2 | Very Low |
| Low dose MDMA | Escitalopram 10mg | 0 | NA | NA | NA | NA | NA | NA | -0.73 (-6.72, 5.16) | Moderate | 0 | Moderate | -0.73 (-6.72, 5.16) | Moderate | 0 | -2 | Very Low |
| Extremely low dose MDMA | Escitalopram 10mg | 0 | NA | NA | NA | NA | NA | NA | -3.53 (-8.34, 1.17) | Moderate | 0 | Moderate | -3.53 (-8.34, 1.17) | Moderate | 0 | -2 | Very Low |
| Escitalopram 20mg | Escitalopram 10mg | 4 | -0.03 (-1.70, 1.56) | 0 | -1 | 0 | 0 | Moderate | NA | NA | NA | NA | -0.03 (-1.70, 1.56) | Moderate | 0 | -2 | Very Low |
| High dose psilocybin | Escitalopram 20mg | 1 | 4.69 (1.64, 7.54) | 0 | 0 | 0 | 0 | High | NA | NA | NA | NA | 4.69 (1.64, 7.54) | High | 0 | -1 | Moderate |
| Low dose psilocybin | Escitalopram 20mg | 0 | NA | NA | NA | NA | NA | NA | 0.33 (-3.66, 4.16) | High | 0 | High | 0.33 (-3.66, 4.16) | High | 0 | -2 | Low |
| Extremely low dose Psilocybin | Escitalopram 20mg | 0 | NA | NA | NA | NA | NA | NA | -1.65 (-5.66, 2.23) | High | 0 | High | -1.65 (-5.66, 2.23) | High | 0 | -2 | Low |
| LSD | Escitalopram 20mg | 0 | NA | NA | NA | NA | NA | NA | 0.81 (-5.12, 6.62) | High | 0 | High | 0.81 (-5.12, 6.62) | High | 0 | -2 | Low |
| Ayahuasca | Escitalopram 20mg | 0 | NA | NA | NA | NA | NA | NA | 3.55 (-2.14, 8.96) | High | 0 | High | 3.55 (-2.14, 8.96) | High | 0 | -2 | Low |
| High dose MDMA | Escitalopram 20mg | 0 | NA | NA | NA | NA | NA | NA | -0.29 (-4.47, 3.84) | High | 0 | High | -0.29 (-4.47, 3.84) | High | 0 | -2 | Low |
| Low dose MDMA | Escitalopram 20mg | 0 | NA | NA | NA | NA | NA | NA | -0.70 (-6.57, 5.11) | High | 0 | High | -0.70 (-6.57, 5.11) | High | 0 | -2 | Low |
| Extremely low dose MDMA | Escitalopram 20mg | 0 | NA | NA | NA | NA | NA | NA | -1.65 (-5.66, 2.23) | High | 0 | High | -1.65 (-5.66, 2.23) | High | 0 | -2 | Low |
| High dose psilocybin | Extremely low dose MDMA | 0 | NA | NA | NA | NA | NA | NA | 8.19 (3.99, 12.41) | High | 0 | High | 8.19 (3.99, 12.41) | High | 0 | 0 | High |
| Low dose psilocybin | Extremely low dose MDMA | 0 | NA | NA | NA | NA | NA | NA | 3.83 (-0.84, 8.51) | High | 0 | High | 3.83 (-0.84, 8.51) | High | 0 | -2 | Low |
| Extremely low dose Psilocybin | Extremely low dose MDMA | 0 | NA | NA | NA | NA | NA | NA | 1.85 (-3.02, 6.76) | High | 0 | High | 1.85 (-3.02, 6.76) | High | 0 | -2 | Low |
| LSD | Extremely low dose MDMA | 0 | NA | NA | NA | NA | NA | NA | 4.31 (-1.99, 10.58) | High | 0 | High | 4.31 (-1.99, 10.58) | High | 0 | -2 | Low |
| Ayahuasca | Extremely low dose MDMA | 0 | NA | NA | NA | NA | NA | NA | 7.05 (1.04, 12.91) | High | 0 | High | 7.05 (1.04, 12.91) | High | 0 | -1 | Moderate |
| High dose MDMA | Extremely low dose MDMA | 3 | 3.21 (0.34, 6.22) | 0 | 0 | 0 | 0 | High | NA | NA | NA | NA | 3.21 (0.34, 6.22) | High | 0 | -1 | Moderate |
| Low dose MDMA | Extremely low dose MDMA | 1 | 2.81 (-2.01, 7.63) | 0 | 0 | 0 | 0 | High | NA | NA | NA | NA | 2.81 (-2.01, 7.63) | High | 0 | -2 | Low |
| High dose psilocybin | Low dose MDMA | 0 | NA | NA | NA | NA | NA | NA | 5.39 (-0.08, 10.89) | High | 0 | High | 5.39 (-0.08, 10.89) | High | 0 | -2 | Low |
| Low dose psilocybin | Low dose MDMA | 0 | NA | NA | NA | NA | NA | NA | 1.03 (-4.80, 6.93) | High | 0 | High | 1.03 (-4.80, 6.93) | High | 0 | -2 | Low |
| Extremely low dose Psilocybin | Low dose MDMA | 0 | NA | NA | NA | NA | NA | NA | -0.96 (-6.96, 5.11) | High | 0 | High | -0.96 (-6.96, 5.11) | High | 0 | -2 | Low |
| LSD | Low dose MDMA | 0 | NA | NA | NA | NA | NA | NA | 1.50 (-5.64, 8.73) | High | 0 | High | 1.50 (-5.64, 8.73) | High | 0 | -2 | Low |
| Ayahuasca | Low dose MDMA | 0 | NA | NA | NA | NA | NA | NA | 4.24 (-2.65, 11.09) | High | 0 | High | 4.24 (-2.65, 11.09) | High | 0 | -2 | Low |
| High dose MDMA | Low dose MDMA | 1 | 0.40 (-4.31, 5.21) | 0 | 0 | 0 | 0 | High | NA | NA | NA | NA | 0.40 (-4.31, 5.21) | High | 0 | -2 | Low |
| High dose psilocybin | High dose MDMA | 0 | NA | NA | NA | NA | NA | NA | 4.98 (1.23, 8.67) | High | 0 | Low | 4.98 (1.23, 8.67) | High | 0 | -1 | Moderate |
| Low dose psilocybin | High dose MDMA | 0 | NA | NA | NA | NA | NA | NA | 0.62 (-3.65, 4.83) | High | 0 | Low | 0.62 (-3.65, 4.83) | High | 0 | -2 | Low |
| Extremely low dose Psilocybin | High dose MDMA | 0 | NA | NA | NA | NA | NA | NA | -1.36 (-5.84, 3.08) | High | 0 | Low | -1.36 (-5.84, 3.08) | High | 0 | -2 | Low |
| LSD | High dose MDMA | 0 | NA | NA | NA | NA | NA | NA | 1.10 (-4.88, 7.06) | High | 0 | Low | 1.10 (-4.88, 7.06) | High | 0 | -2 | Low |
| Ayahuasca | High dose MDMA | 0 | NA | NA | NA | NA | NA | NA | 3.84 (-1.84, 9.32) | High | 0 | Low | 3.84 (-1.84, 9.32) | High | 0 | -2 | Low |
| High dose psilocybin | Ayahuasca | 0 | NA | NA | NA | NA | NA | NA | 1.15 (-3.92, 6.35) | High | 0 | Low | 1.15 (-3.92, 6.35) | High | 0 | -2 | Low |
| Low dose psilocybin | Ayahuasca | 0 | NA | NA | NA | NA | NA | NA | -3.22 (-8.67, 2.39) | High | 0 | Low | -3.22 (-8.67, 2.39) | High | 0 | -2 | Low |
| Extremely low dose Psilocybin | Ayahuasca | 0 | NA | NA | NA | NA | NA | NA | -5.20 (-10.80, 0.60) | High | 0 | Low | -5.20 (-10.80, 0.60) | High | 0 | -2 | Low |
| LSD | Ayahuasca | 0 | NA | NA | NA | NA | NA | NA | -2.74 (-9.57, 4.21) | High | 0 | Low | -2.74 (-9.57, 4.21) | High | 0 | -2 | Moderate |
| High dose psilocybin | LSD | 0 | NA | NA | NA | NA | NA | NA | 3.88 (-1.66, 9.44) | High | 0 | Low | 3.88 (-1.66, 9.44) | High | 0 | -2 | Low |
| Low dose psilocybin | LSD | 0 | NA | NA | NA | NA | NA | NA | -0.48 (-6.37, 5.51) | High | 0 | Low | -0.48 (-6.37, 5.51) | High | 0 | -2 | Low |
| Extremely low dose Psilocybin | LSD | 0 | NA | NA | NA | NA | NA | NA | -2.46 (-8.48, 3.61) | High | 0 | Low | -2.46 (-8.48, 3.61) | High | 0 | -2 | Low |
| High dose psilocybin | Extremely low dose Psilocybin | 2 | 6.35 (3.41, 9.21) | 0 | 0 | 0 | 0 | High | NA | NA | NA | NA | 6.35 (3.41, 9.21) | High | 0 | -1 | Moderate |
| Low dose psilocybin | Extremely low dose Psilocybin | 1 | 1.99 (-1.71, 5.65) | 0 | 0 | 0 | 0 | High | 2.47 (-2.35, 7.19) | High | 0 | High | 1.99 (-1.71, 5.65) | High | 0 | -2 | Low |
| High dose psilocybin | Low dose psilocybin | 1 | 4.77 (0.64, 8.80) | 0 | 0 | 0 | 0 | High | 3.94 (-0.12, 8.06) | High | 0 | High | 4.36 (1.18, 7.52) | High | 0 | -1 | Moderate |

Appendix 1. The Preferred Reporting Items for Systematic Reviews and Meta-analyses (PRISMA) extension statement for reporting systematic reviews incorporating network meta-analysis

**Appendix 1**. Checklist of PRISMA guideline

| Section and Topic | Item # | Checklist item | Location where item is reported |
|---|---|---|---|
| **TITLE** | | | |
| Title | 1 | Identify the report as a systematic review incorporating a network meta-analysis | Title page |
| **ABSTRACT** | | | |
| Abstract | 2 | Provide a structured summary including, as applicable:<br>Background: main objectives<br>Methods: data sources; study eligibility criteria, participants, and interventions; study appraisal;<br>and synthesis methods, such as network meta-analysis.<br>Results: number of studies and participants identified; summary estimates with corresponding<br>confidence/credible intervals; treatment rankings may also be discussed. Authors may choose<br>to summarize pairwise comparisons against a chosen treatment included in their analyses for<br>brevity.<br>Discussion/Conclusions: limitations; conclusions and implications of findings.<br>Other: primary source of funding; systematic review registration number with registry name | Abstract section |
| **INTRODUCTION** | | | |
| Rationale | 3 | Describe the rationale for the review in the context of what is already known, including mention of<br>why a network meta-analysis has been conducted.. | 1st – 3rd paragraph of Introduction |
| Objectives | 4 | Provide an explicit statement of questions being addressed, with reference to participants,<br>interventions, comparisons, outcomes, and study design (PICOS). | 4th paragraph of Introduction |
| **METHODS** | | | |
| Protocol and | 5 | Indicate whether a review protocol exists and if and where it | PROSPERO |

| Section and Topic | Item # | Checklist item | Location where item is reported |
|---|---|---|---|
| registration | | can be accessed (e.g., Web address); and, if available, provide registration information, including registration number | (CRD42023469014) |
| Eligibility criteria | 6 | Specify study characteristics (e.g., PICOS, length of follow-up) and report characteristics (e.g., years considered, language, publication status) used as criteria for eligibility, giving rationale. Clearly describe eligible treatments included in the treatment network, and note whether any have been clustered or merged into the same node (with justification). | paragraph of “Study selection” of Method section |
| Information sources | 7 | Describe all information sources (e.g., databases with dates of coverage, contact with study authors to identify additional studies) in the search and date last searched | paragraph of Data sources and searches of Method section |
| Search | 8 | Present full electronic search strategy for at least one database, including any limits used, such that it could be repeated | paragraph of Data sources and searches of Method section |
| Selection process | 9 | State the process for selecting studies (i.e., screening, eligibility, included in systematic review, and, if applicable, included in the meta-analysis). | paragraph of Study selection of Method section |
| Data collection process | 10 | Describe method of data extraction from reports (e.g., piloted forms, independently, in duplicate) and any processes for obtaining and confirming data from investigators | paragraph of Study selection of Method section |
| Data items | 11 | List and define all variables for which data were sought (e.g., PICOS, funding sources) and any assumptions and simplifications made | paragraph of Study selection of Method section |
| Geometry of the network | S1 | Describe methods used to explore the geometry of the treatment network under study and potential biases related to it. This should include how the evidence base has been graphically summarized for presentation, and what characteristics were compiled and used to describe | paragraph of Definition of outcomes, data extraction, and risk of bias assessment of Method section |

| Section and Topic | Item # | Checklist item | Location where item is reported |
|---|---|---|---|
| | | the evidence base to readers | |
| Study risk of bias within individual studies | 12 | Describe methods used for assessing risk of bias of individual studies (including specification of whether this was done at the study or outcome level), and how this information is to be used in any data synthesis. | paragraph of Definition of outcomes, data extraction, and risk of bias assessment of Method section |
| Summary measures | 13 | State the principal summary measures (e.g., risk ratio, difference in means). Also describe the use of additional summary measures assessed, such as treatment rankings and surface under the cumulative ranking curve (SUCRA) values, as well as modified approaches used to present summary findings from meta-analyses. | paragraph of Data synthesis of Method section |
| Planned methods of analysis | 14 | Describe the processes used to decide which studies were eligible for each synthesis (e.g. tabulating the study intervention characteristics and comparing against the planned groups for each synthesis (item #5)). | paragraph of Data synthesis of Method section |
| Assessment of inconsistency | S2 | Describe the statistical methods used to evaluate the agreement of direct and indirect evidence in the treatment network(s) studied. Describe efforts taken to address its presence when found | paragraph of Assessment certainty of evidence for the primary outcome of Method section |
| Risk of bias across studies | 15 | Specify any assessment of risk of bias that may affect the cumulative evidence (e.g., publication bias, selective reporting within studies). | paragraph of Definition of outcomes, data extraction, and risk of bias assessment of Method section |
| Additional analyses | 16 | Describe methods of additional analyses if done, indicating which were prespecified. This may include, but not be limited to, the following: Sensitivity or subgroup analyses; Meta-regression analyses; Alternative formulations of the treatment network; and Use of alternative | paragraph of Data synthesis of Method section |

| Section and Topic | Item # | Checklist item | Location where item is reported |
|---|---|---|---|
| | | prior distributions for Bayesian analyses (if applicable). | |
| **RESULTS** | | | |
| Study selection | 17 | Give numbers of studies screened, assessed for eligibility, and included in the review, with reasons for exclusions at each stage, ideally with a flow diagram. | e-Figure 1 |
| Presentation of network structure | S3 | Provide a network graph of the included studies to enable visualization of the geometry of the treatment network. | Figure 1 |
| Summary of network geometry | S4 | Provide a brief overview of characteristics of the treatment network. This may include commentary on the abundance of trials and randomized patients for the different interventions and pairwise comparisons in the network, gaps of evidence in the treatment network, and potential biases reflected by the network structure. | paragraph of Network meta-analysis of Result section |
| Study characteristics | 18 | For each study, present characteristics for which data were extracted (e.g., study size, PICOS, follow-up period) and provide the citations. | e-Table 1 |
| Risk of bias within studies | 19 | Present data on risk of bias of each study and, if available, any outcome level assessment. | e-Figure 2, e-Figure 3 |
| Results of individual studies | 20 | For all outcomes considered (benefits or harms), present, for each study: 1) simple summary data for each intervention group, and 2) effect estimates and confidence intervals. Modified approaches may be needed to deal with information from larger networks. | e-Figure 4 |
| Syntheses of results | 21 | Present results of each meta-analysis done, including confidence/credible intervals. In larger networks, authors may focus on comparisons versus a particular comparator (e.g., placebo or standard care), with full findings presented in an appendix. League tables and forest plots may be considered to summarize pairwise comparisons. If additional summary measures were explored (such as treatment rankings), these should also be presented | Figure 2, paragraph of Network meta-analysis of Result section |

| Section and Topic | Item # | Checklist item | Location where item is reported |
|---|---|---|---|
| Reporting biases | 21 | Present assessments of risk of bias due to missing results (arising from reporting biases) for each synthesis assessed. | e-Figure 2, e-Figure 3, paragraph of Quality of the included studies of Result section |
| Exploration for inconsistency | S5 | Describe results from investigations of inconsistency. This may include such information as measures of model fit to compare consistency and inconsistency models, P values from statistical tests, or summary of inconsistency estimates from different parts of the treatment network. | paragraph of Transitivity assumption of Result section, eFigure 5-6 |
| Risk of bias across studies | 22 | Present results of any assessment of risk of bias across studies for the evidence base being studied. | e-Figure 2, e-Figure 3 |
| Results of additional analyses | 23 | Give results of additional analyses, if done (e.g., sensitivity or subgroup analyses, meta-regression analyses, alternative network geometries studied, alternative choice of prior distributions for Bayesian analyses, and so forth). | Figure 3, Figure 4, paragraph of Sensitivity analyses of Result section, Figure 3, eFigure 8-10 |
| **DISCUSSION** | | | |
| Summary of evidence | 24 | Summarize the main findings, including the strength of evidence for each main outcome; consider their relevance to key groups (e.g., health care providers, researchers, and policymakers). | 1st paragraph of Discussion |
| Limitations | 25 | Discuss limitations at study and outcome level (e.g., risk of bias), and at review level (e.g., incomplete retrieval of identified research, reporting bias). Comment on the validity of the assumptions, such as transitivity and consistency. Comment on any concerns regarding network geometry (e.g., avoidance of certain comparisons). | The paragraph of Strengths and limitations of this study of Discussion |
| Conclusions | 26 | Provide a general interpretation of the results in the context of | The paragraph of |

| Section and Topic | Item # | Checklist item | Location where item is reported |
|---|---|---|---|
| | | other evidence, and implications for future research. | Implications and conclusions Discussion |
| **OTHER INFORMATION** | | | |
| Funding | 27 | Describe sources of funding for the systematic review and other support (e.g., supply of data); role of funders for the systematic review. This should also include information regarding whether funding has been received from manufacturers of treatments in the network and/or whether some of the authors are content experts with professional conflicts of interest that could affect use of treatments in the network. | Bottom of the manuscript |

Appendix 2. The complete search strategies

| Database |
| --- |
| **MEDLINE search strategy** |
| 1 exp Psilocybin/ or Psilocybin.mp. or magic mushrooms.mp. (1,720)<br>2 ayahuasca.mp. or exp Banisteriopsis/ (501)<br>3 exp Lysergic Acid Diethylamide/ or Lysergic Acid Diethylamide.mp. or LSD.mp. (9,508)<br>4 exp Lysergic Acid/ or Lysergic Acid.mp. (5,920)<br>5 exp N-Methyl-3,4-methylenedioxyamphetamine/ or N-Methyl-3,4-methylenedioxyamphetamine.mp. or MDMA.mp. (5,833)<br>6 exp escitalopram/ (189)<br>7 escitalopram.mp. (3,226)<br>8 N,N-Dimethyltryptamine/ (439)<br>9 (DMT or 5-MeO-DMT).mp. (3,179)<br>10 mescaline.mp. or exp Mescaline/ (1,320)<br>11 exp Depression/ or Depression.mp. (492,128)<br>12 depress*.mp. (647,538)<br>13 11 or 12 (647,538)<br>14 1 or 2 or 3 or 4 or 5 or 6 or 7 or 8 or 9 or 10 (23,730)<br>15 13 and 14 (3,488)<br>16 clinical trial.mp. or exp Clinical Trial/ (1,094,828)<br>17 15 and 16 (757) |
| **Cochrane Central Register of Controlled Trials (CENTRAL) search strategy** |
| #1 MeSH descriptor: [Psilocybin] explode all trees (153)<br>#2 (Psilocybin):ti,ab,kw (307)<br>#3 (magic mushrooms):ti,ab,kw (11)<br>#4 (MDMA):ti,ab,kw (409)<br>#5 MeSH descriptor: [N-Methyl-3,4-methylenedioxyamphetamine] explode all trees (242)<br>#6 MeSH descriptor: [Lysergic Acid Diethylamide] explode all trees (132)<br>#7 (Lysergic Acid Diethylamide):ti,ab,kw (205)<br>#8 MeSH descriptor: [Escitalopram] explode all trees (51)<br>#9 (escitalopram):ti,ab,kw (2,014)<br>#10 (ayahuasca):ti,ab,kw (49)<br>#11 MeSH descriptor: [Banisteriopsis] 1 tree(s) exploded (0)<br>#12 (DMT):ti,ab,kw (485)<br>#13 MeSH descriptor: [Mescaline] explode all trees (9)<br>#14 (mescaline):ti,ab,kw (20) |

| | |
|---|---|
| #15 | #1 or #2 or #3 or #4 or #5 or #6 or #7 or #8 or #9 #10 or #11 or #12 or #13 or #14 (1417) |
| #16 | MeSH descriptor: [Depression] 1 tree(s) exploded (18679) |
| #17 | (Depression):ti,ab,kw (98,664) |
| #18 | #16 or #17 (98,664) |
| #19 | #15 and #18 (267) |
| #20 | ("randomized controlled trial" or RCT):ti,ab,kw (669,993) |
| #21 | #19 and #20 (110) |
| **Embase search strategy** | |
| #1. | 'lysergic acid diethylamide'/exp OR 'lysergic acid diethylamide' OR lsd (16,219) |
| #2. | 'escitalopram'/exp (16,026) |
| #3. | escitalopram (16,439) |
| #4. | 'psilocybin'/exp OR psilocybin (3,067) |
| #5. | 'ayahuasca'/exp OR ayahuasca (667) |
| #6. | 'dmt' (7,338) |
| #7. | 'n,n dimethyltryptamine'/exp OR 'n,n dimethyltryptamine' (1,812) |
| #8. | 'mescaline'/exp OR mescaline (2,523) |
| #9. | 'banisteriopsis'/exp OR banisteriopsis (284) |
| #10. | 'magic mushrooms' (163) |
| #11. | 'n,n dimethyltryptamine'/exp OR 'n,n dimethyltryptamine' (1,812) |
| #12. | 'n methyl 3,4 methylenedioxyamphetamine' (47) |
| #13. | 'n methyl 3,4 methylenedioxyamphetamine'/de (10,457) |
| #14. | 'depression'/exp OR depression (929,175) |
| #15. | #1 OR #2 OR #3 OR #4 OR #5 OR #6 OR #7 OR #8 OR #9 OR #10 OR #11 OR #12 OR #13 (52,713) |
| #16. | #14 AND #15 (13,979) |
| #17. | #16 AND 'randomized controlled trial'/de (1,121) |
| **PsycINFO search strategy** | |
| S1 | psilocybin OR magic mushrooms (906) |
| S2 | ayahuasca OR Banisteriopsis (407) |
| S3 | lysergic acid diethylamide OR LSD (3,239) |
| S4 | N-Methyl-3,4-methylenedioxyamphetamine OR MDMA (2,854) |
| S5 | escitalopram (2,910) |
| S6 | N,N-Dimethyltryptamine (249) |
| S7 | DMT (2,259) |
| S8 | mescaline (625) |
| S9 | S1 OR S2 OR S3 OR S4 OR S5 OR S6 OR S7 OR S8 (11,902) |
| S10 | depression or depressive disorder or depressive symptoms or major depressive disorder (418,881) |
| S11 | (S9 AND S10) (3,040) |

| | |
|---|---|
| S12 (clinical trial AND S11) | (1,001) |
| **ClinicalTrials.gov (https://clinicaltrials.gov/)** | |
| Psilocybin \| Depression (52) | |
| Ayahuasca \| Depression (2) | |
| lysergic acid diethylamide \| Depression (2) | |
| N-Methyl-3,4-methylenedioxyamphetamine \| Depression (5) | |
| Escitalopram \| Depression (460) | |
| Mescaline \| Depression (0) | |
| **World Health Organization (ICTRP) (https://trialsearch.who.int)** | |
| Psilocybin and Depression (53) | |
| Ayahuasca and Depression (1) | |
| lysergic acid diethylamide and Depression (2) | |
| N-Methyl-3,4-methylenedioxyamphetamine and Depression (0) | |
| Escitalopram and Depression (404) | |
| Mescaline and Depression (0) | |

CENTRAL: Cochrane Central Register of Controlled Trials

Appendix 3. Reasons for exclusion

**Duplicated (n=9)**

Barba T, Buehler S, Kettner H, et al. Effects of psilocybin versus escitalopram on rumination and thought suppression in depression. *BJPsych open* 2022;8(5) doi: 10.1192/bjo.2022.565

Goodwin GM, Aaronson ST, Alvarez O, et al. Single-dose psilocybin for a treatment-resistant episode of major depression: impact on patient-reported depression severity, anxiety, function, and quality of life. *Journal of affective disorders* 2023;327:120-27. doi: 10.1016/j.jad.2023.01.108

Goodwin GM, Croal M, Feifel D, et al. Psilocybin for treatment resistant depression in patients taking a concomitant SSRI medication. *Neuropsychopharmacology* 2023;48(10):1492-99. doi: 10.1038/s41386-023-01648-7

Goodwin GM, Marwood L, Mistry S, et al. Improvement in Depression Symptoms Measured by Montgomery-Åsberg Depression Rating Scale and Quick Inventory of Depressive Symptomatology-Self Rated Items after Randomised Double-blind COMP360 Psilocybin Therapy for Treatment-resistant Depression. *European Psychiatry* 2023;66:S91-S92. doi: 10.1192/j.eurpsy.2023.273

Mithoefer MC, Feduccia AA, Jerome L, et al. MDMA-assisted psychotherapy for treatment of PTSD: study design and rationale for phase 3 trials based on pooled analysis of six phase 2 randomized controlled trials. *Psychopharmacology* 2019;236(9):2735-45. doi: 10.1007/s00213-019-05249-5

Murphy R, Kettner H, Zeifman R, et al. Therapeutic Alliance and Rapport Modulate Responses to Psilocybin Assisted Therapy for Depression. *Frontiers in pharmacology* 2022;12 doi: 10.3389/fphar.2021.788155

Weiss B, Ginige I, Shannon L, et al. Personality change in a trial of psilocybin therapy v. escitalopram treatment for depression. *Psychological medicine* 2023:1-15. doi: 10.1017/S0033291723001514

Zeifman RJ, Palhano-Fontes F, Hallak J, et al. The impact of ayahuasca on suicidality: Results from a randomized controlled trial. *Frontiers in Pharmacology* 2019;10 doi: 10.3389/fphar.2019.01325

Zeifman RJ, Wagner AC, Monson CM, et al. How does psilocybin therapy work? An exploration of experiential avoidance as a putative mechanism of change. *Journal of affective disorders* 2023;334:100-12. doi: 10.1016/j.jad.2023.04.105

**Outcome not interest (n=11)**

Asakura S, Hayano T, Hagino A, et al. A randomized, double-blind, placebo-controlled study of escitalopram in patients with social anxiety disorder in Japan. *Current Medical Research and Opinion* 2016;32(4):749-57. doi: 10.1185/03007995.2016.1146663

Feduccia AA, Jerome L, Mithoefer MC, et al. Discontinuation of medications classified as reuptake inhibitors affects treatment response of MDMA-assisted psychotherapy. *Psychopharmacology* 2021;238(2):581-88. doi: 10.1007/s00213-020-05710-w

Gukasyan N, Davis AK, Barrett FS, et al. Efficacy and safety of psilocybin-assisted treatment for major depressive disorder: prospective 12-month follow-up. *Journal of psychopharmacology (Oxford, England)* 2022;36(2):151-58. doi: 10.1177/02698811211073759

Lader M, Stender K, Bürger V, et al. Efficacy and tolerability of escitalopram in 12- and 24-week treatment

of social anxiety disorder: Randomised, double-blind, placebo-controlled, fixed-dose study. *Depression and Anxiety* 2004;19(4):241-48. doi: 10.1002/da.20014

Lee EJ, Kim JS, Chang DI, et al. Post-Stroke Depressive Symptoms: varying Responses to Escitalopram by Individual Symptoms and Lesion Location. *Journal of geriatric psychiatry and neurology* 2021;34(6):565-73. doi: 10.1177/0891988720957108

Marschall J, Fejer G, Lempe P, et al. Psilocybin microdosing does not affect emotion-related symptoms and processing: A preregistered field and lab-based study. *Journal of psychopharmacology (Oxford, England)* 2022;36(1):97-113. doi: https://dx.doi.org/10.1177/02698811211050556

Nakagome K, Yokoi Y, Nakagawa A, et al. Acceptability of escitalopram versus duloxetine in outpatients with depression who did not respond to initial second-generation antidepressants: A randomized, parallel-group, non-inferiority trial. *Journal of Affective Disorders* 2021;282:1011-20. doi: 10.1016/j.jad.2020.12.148

Shen Y, Zhao Q, Yu Y, et al. Efficacy and safety of bupropion hydrochloride extended-release versus escitalopram oxalate in Chinese patients with major depressive disorder: Results from a randomized, double-blind, non-inferiority trial. *Journal of Affective Disorders* 2019;257:143-49. doi: 10.1016/j.jad.2019.07.023

Shin C, Jeon SW, Lee S-H, et al. Efficacy and safety of escitalopram, desvenlafaxine, and vortioxetine in the acute treatment of anxious depression: A randomized rater-blinded 6-week clinical trial. *Clinical Psychopharmacology and Neuroscience* 2023;21(1):135-46. doi: 10.9758/cpn.2023.21.1.135

Yao XW, Li YL, Yu ZJ, et al. The efficacy and safety of agomelatine, sertraline, and escitalopram for senile post-stroke depression: A randomized double-blind placebo-controlled trial. *Clinical Neurology and Neurosurgery* 2021;205 doi: 10.1016/j.clineuro.2021.106651

Zuilhof Z, Norris S, Blondeau C, et al. Optimized regimens of combined medications for the treatment of major depressive disorder: A double-blind, randomized-controlled trial. *Neuropsychiatric Disease and Treatment* 2018;14 doi: 10.2147/NDT.S175203

**Comment, Conference abstract, protocol, or review (n=3)**

Griffiths R, Barrett F, Darrick M, et al. Psilocybin-assisted treatment of major depressive disorder: results from a randomized trial. *Neuropsychopharmacology* 2019;44:439. doi: 10.1038/s41386-019-0547-9

Husain MI, Blumberger DM, Castle DJ, et al. Psilocybin for treatment-resistant depression without psychedelic effects: study protocol for a 4-week, double-blind, proof-of-concept randomised controlled trial. *BJPsych open* 2023;9(4):e134. doi: 10.1192/bjo.2023.535

Rosenblat J, McIntyre R. Psilocybin Assisted Therapy for Treatment-Resistant Depression: a Phase II, Randomized, Feasibility Study. *Neuropsychopharmacology* 2022;47:203. doi: 10.1038/s41386-022-01484-1

**Not Randomized controlled trials (n=3)**

Osório FdL, Sanches RF, Macedo LR, et al. Antidepressant effects of a single dose of ayahuasca in patients with recurrent depression: A preliminary report. *Revista Brasileira de Psiquiatria* 2015;37(1):13-20.

doi: 10.1590/1516-4446-2014-1496
Sanches RF, de Lima Osório F, dos Santos RG, et al. Antidepressant effects of a single dose of ayahuasca in patients with recurrent depression: A spect study. *Journal of Clinical Psychopharmacology* 2016;36(1):77-81. doi: 10.1097/JCP.0000000000000436
Sloshower J, Skosnik PD, Safi-Aghdam H, et al. Psilocybin-assisted therapy for major depressive disorder: An exploratory placebo-controlled, fixed-order trial. J Psychopharmacol. 2023;37(7):698-706. doi:10.1177/02698811231154852

Appendix 4. Prior settings and results of convergence

**Prior settings**

Prior distribution for the intercept: A Normal prior distribution: location = 0, scale = 5.

Prior distribution for the treatment: A Normal prior distribution: location = 0, scale = 5.

Prior distribution for the heterogeneity: A half-Cauchy prior distribution: location = 0, scale = 1.

Prior distribution for the heterogeneity type: sd.

Prior distribution for the regression coefficients: A Normal prior distribution: location = 0, scale = 10.

Prior distribution for the auxiliary parameter: Null

**Results of convergence** Trace plot for the main results

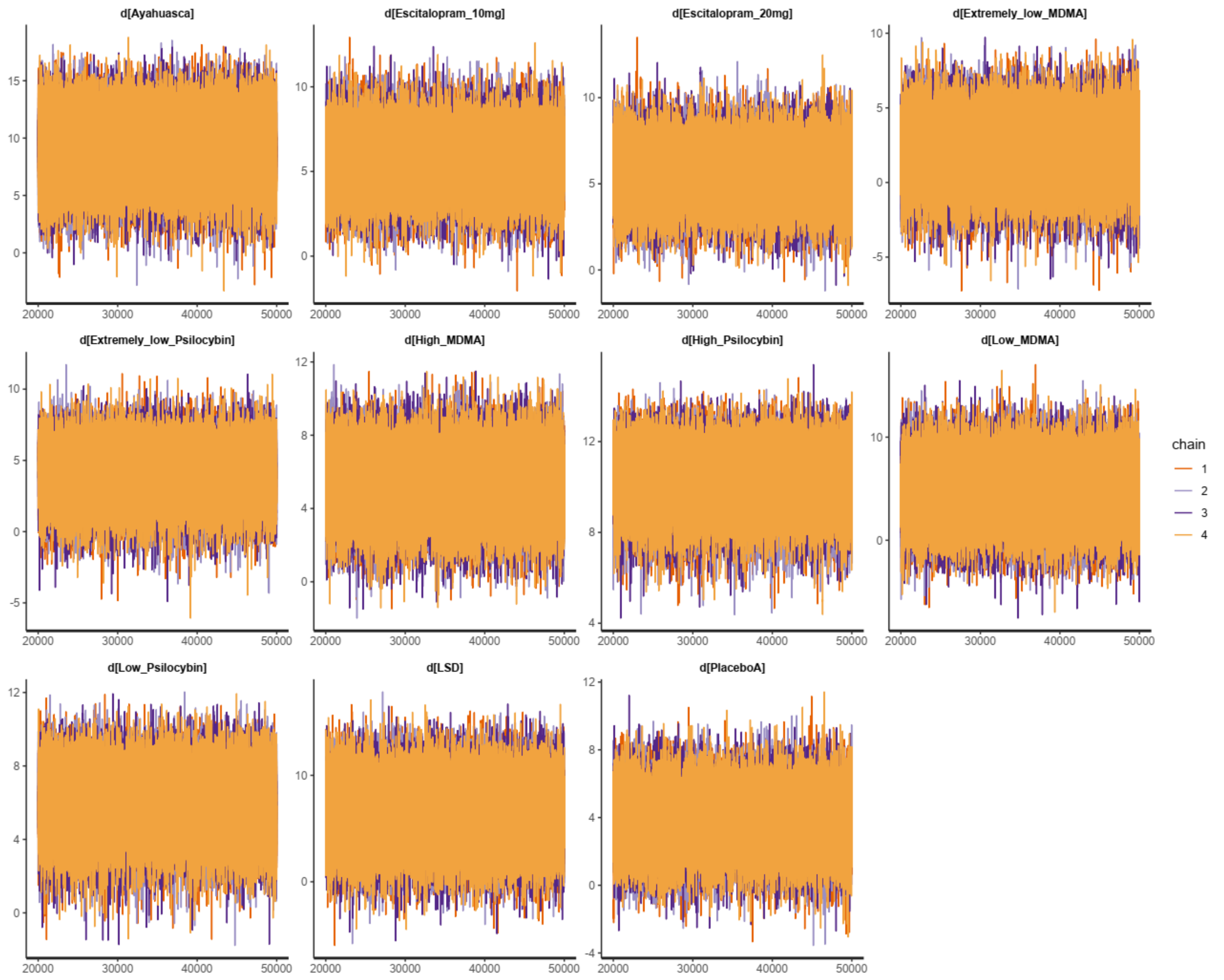

Trace plot for the examination of transitivity assumption of escitalopram part 1

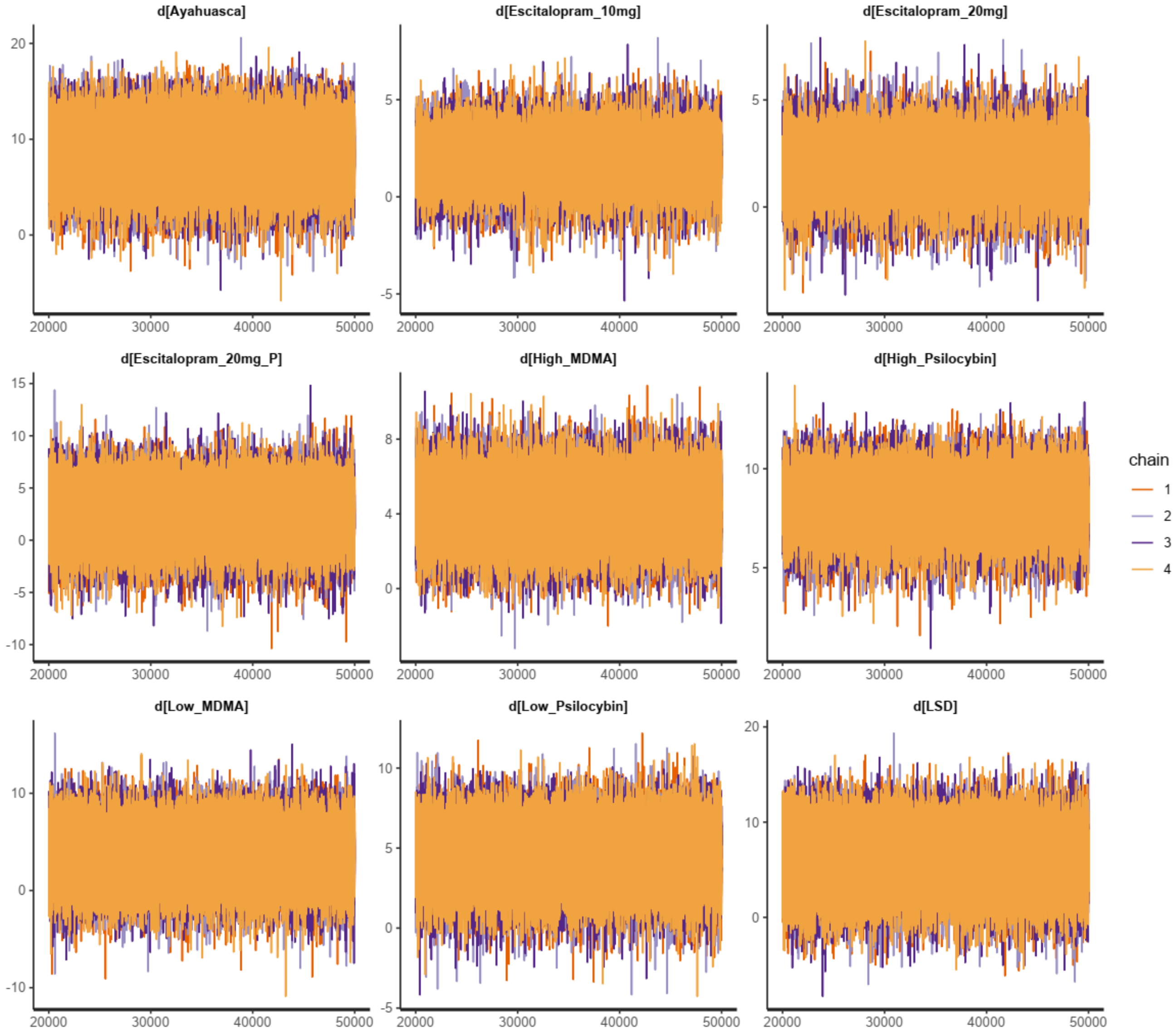

Trace plot for the examination of transitivity assumption of escitalopram part 2

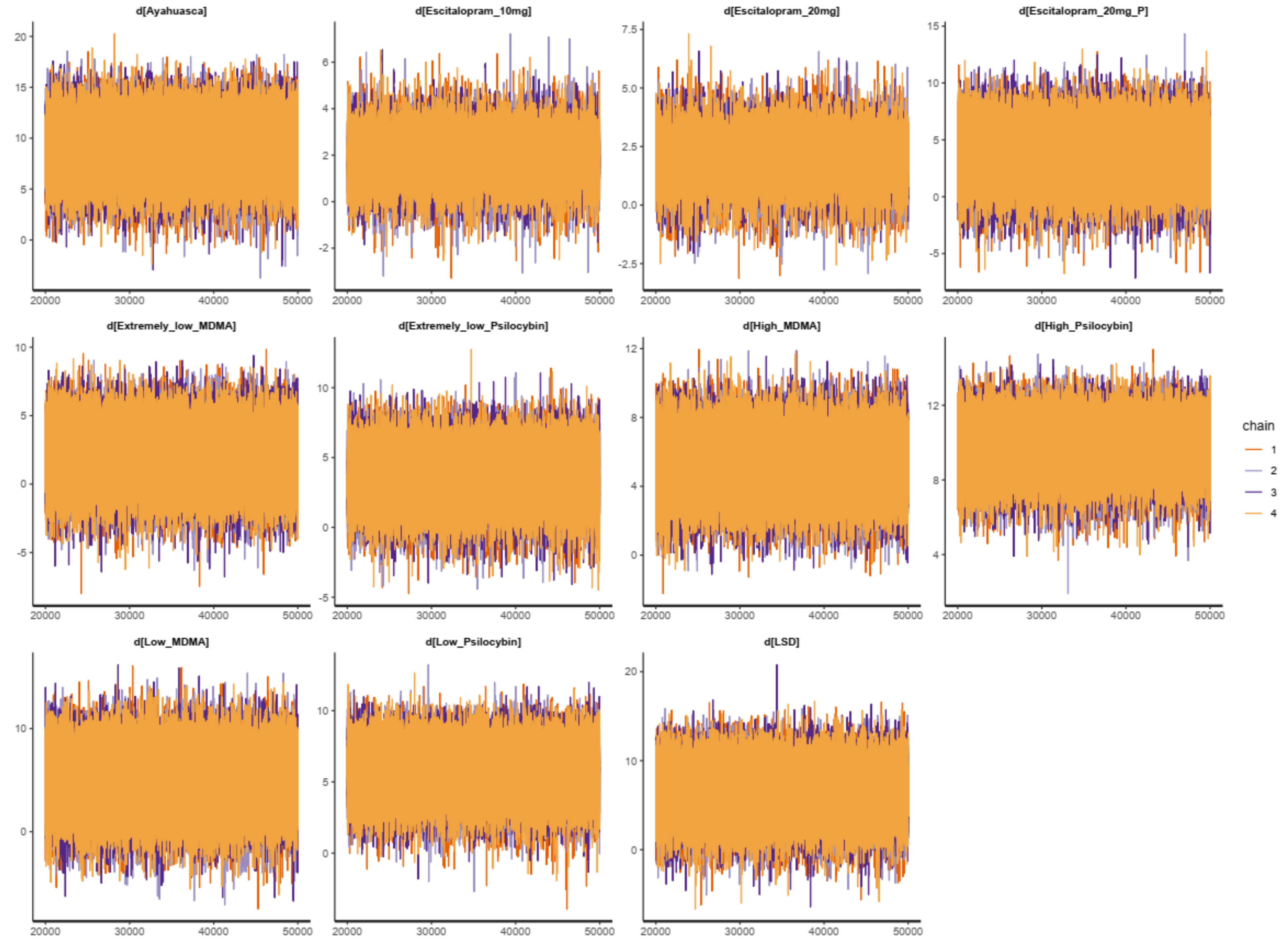

Trace plot for the sensitivity analysis 1: adding a trial with enhancing blinding and minimizing expectancy

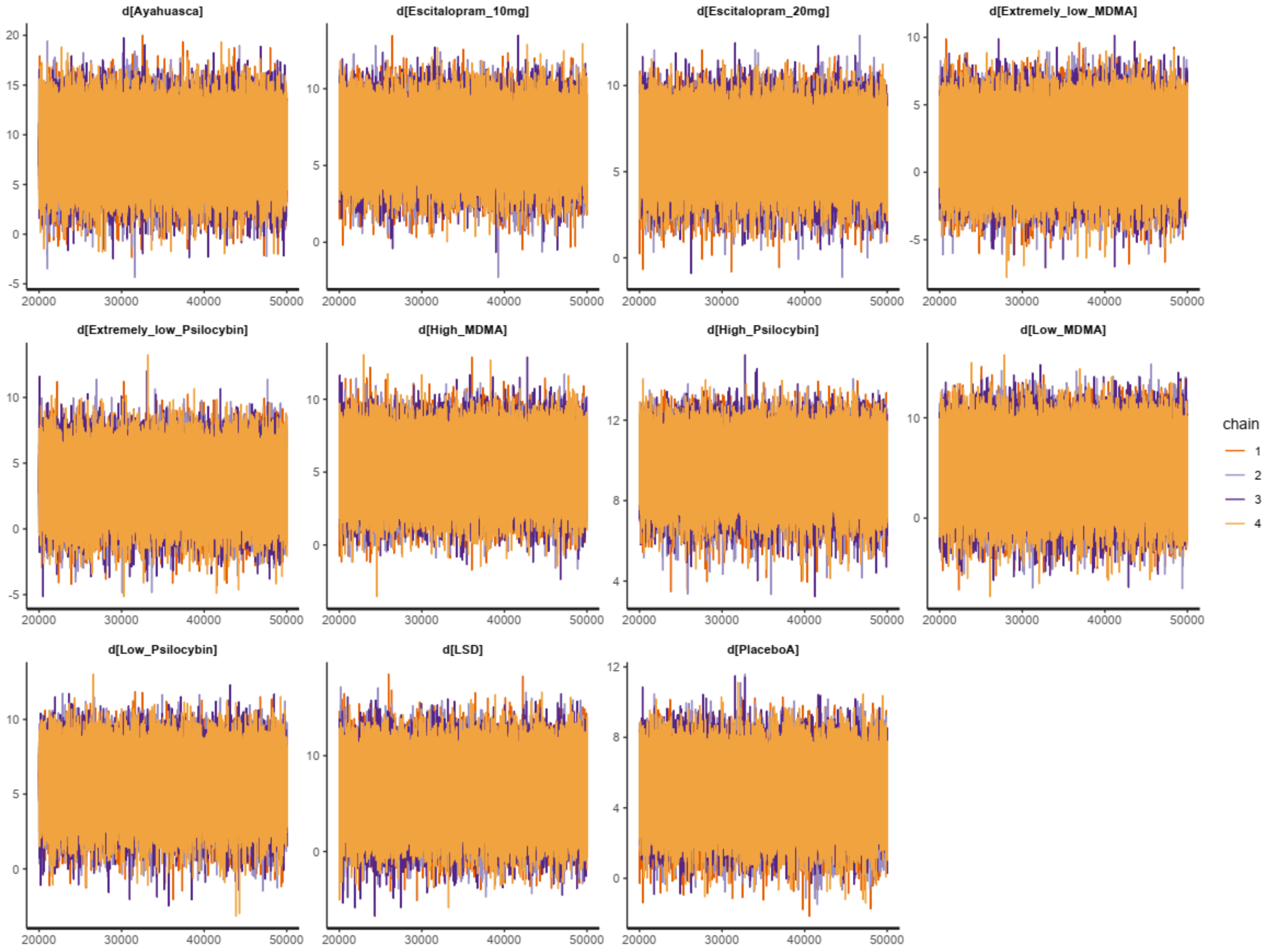

Trace plot for the sensitivity analysis 2: including only studies of patients with major depressive disorder

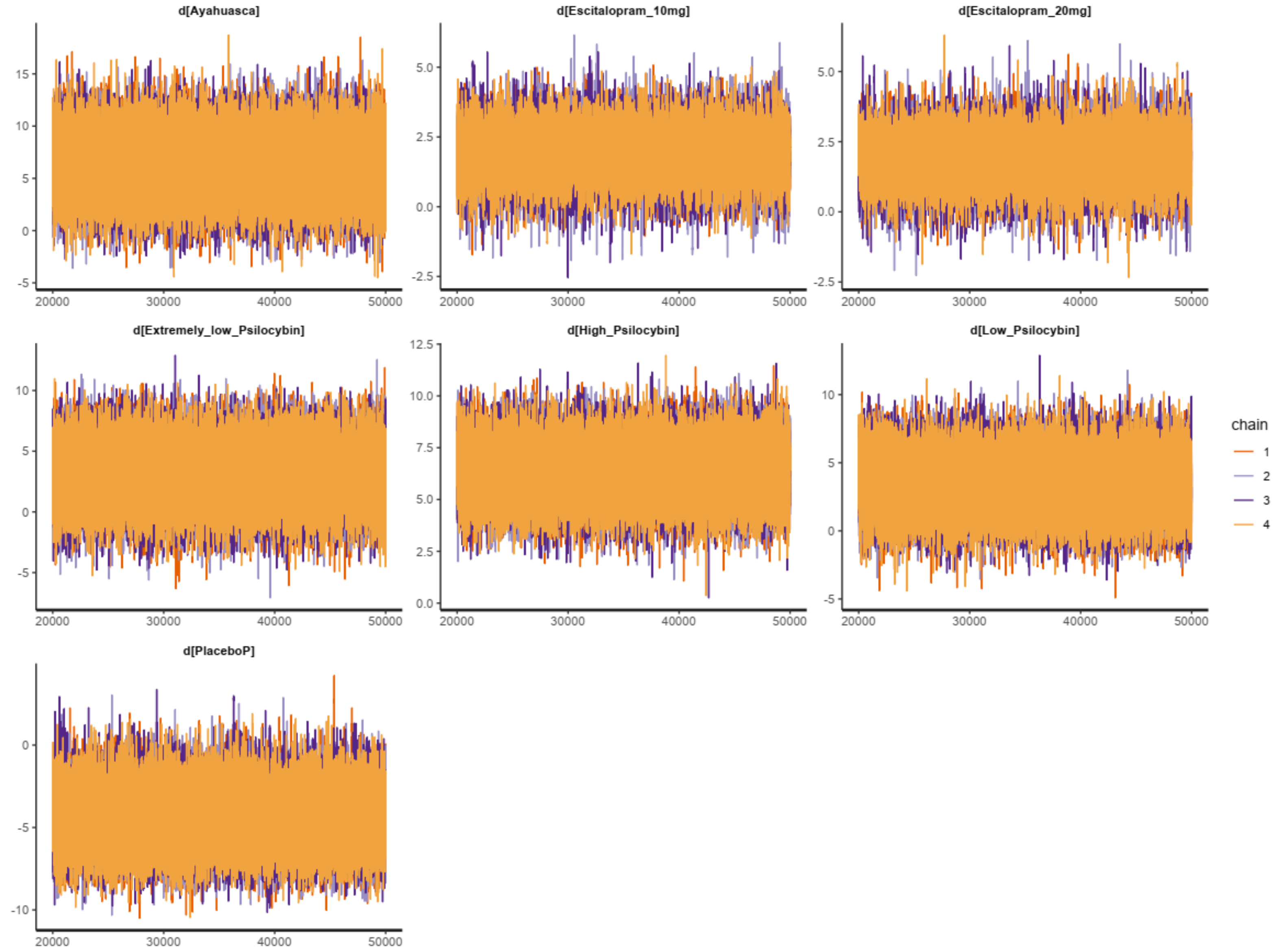

Trace plot for the sensitivity analysis 3: excluding studies with high risk of bias

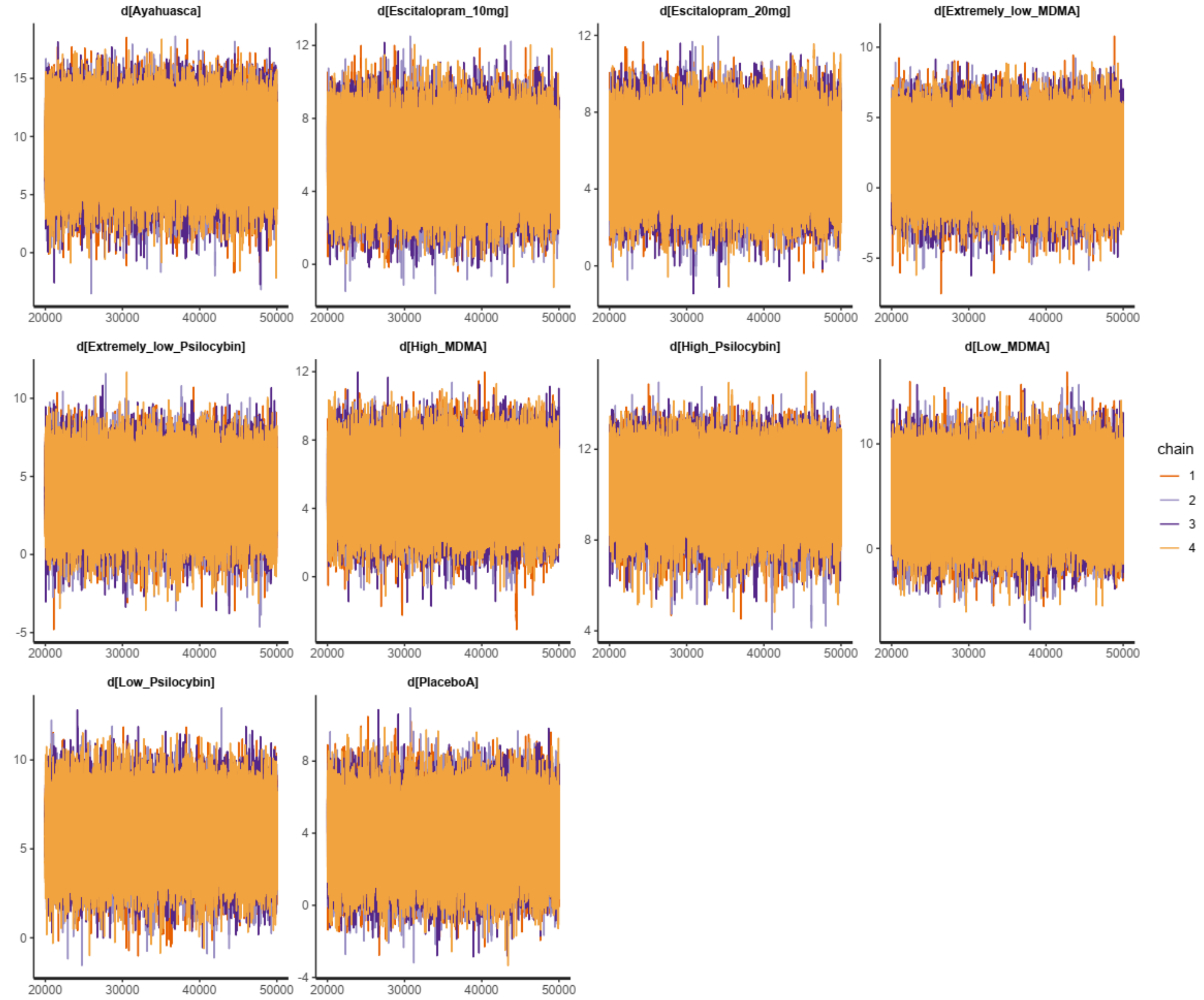

Trace plot for the sensitivity analysis 4: adjusting for baseline depression severity

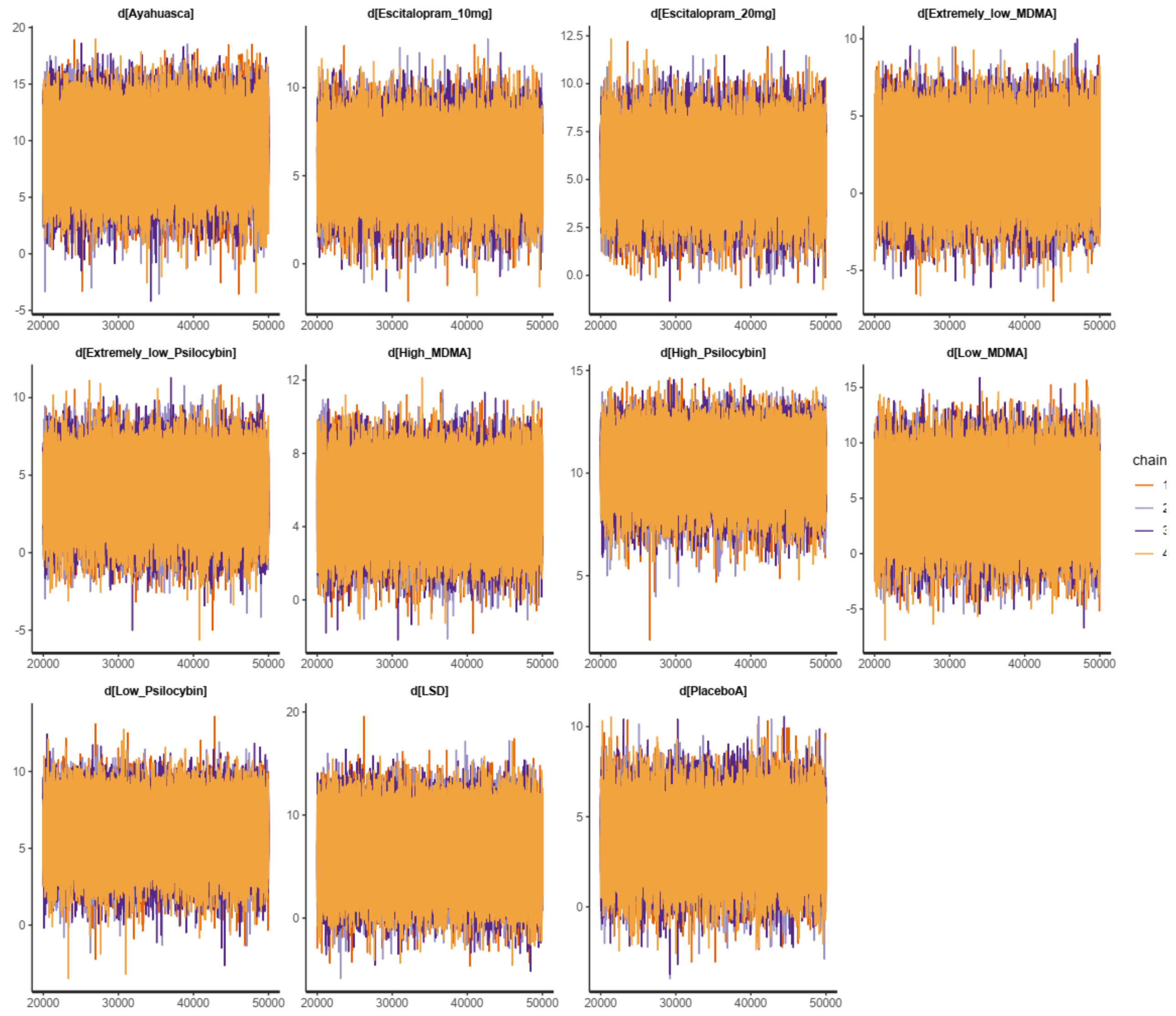

Trace plot for the sensitivity analysis 5: using most conservative correlation coefficient of 0

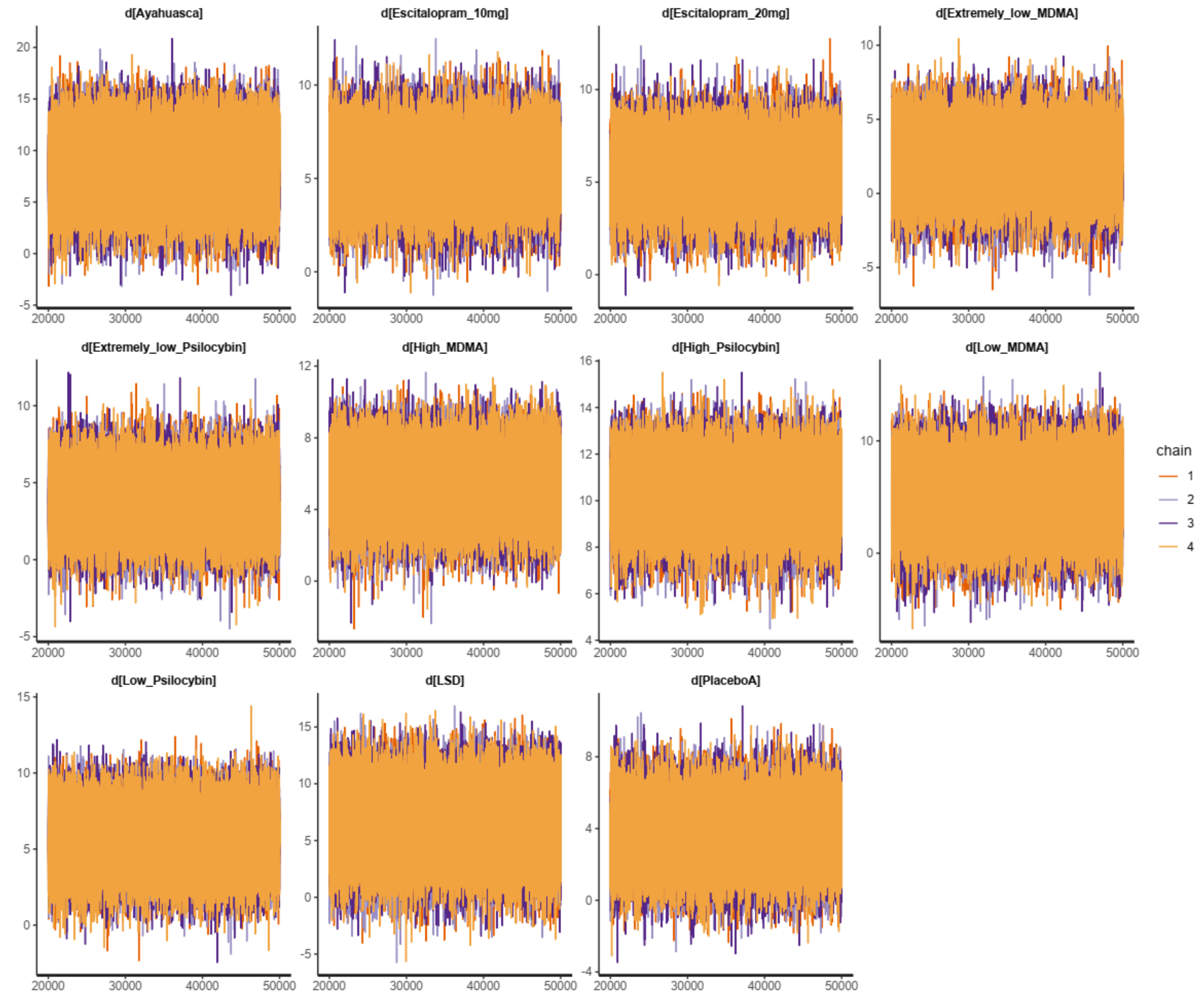

Trace plot for the pairwise meta-analytic estimate of the efficacy of escitalopram 20mg in two different study designs

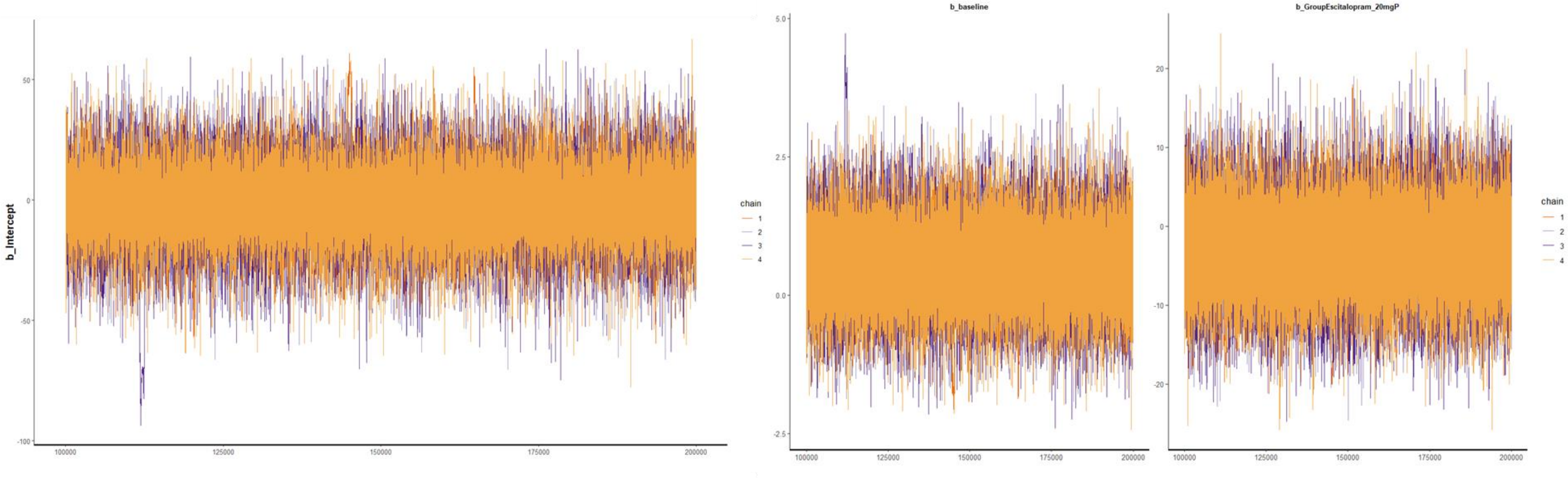

Appendix 5. Reasons for protocol changes

1. In the registered PROSPERO protocol, the risk of bias was assessed by Cochrane risk-of-bias tool Version 1. Now we used the Cochrane risk-of-bias tool Version 2. This is because Version 2 of the Cochrane risk-of-bias tool for randomized trials (RoB 2) is the recommended tool to assess the risk of bias in randomized trials included in Cochrane Reviews.
2. In the registered PROSPERO protocol, only subgroup analyses were mentioned. In the current paper, we also conducted network meta-regression for the primary outcome (change in depressive symptoms) to determine potential effect modifiers, including female proportion, mean age, and baseline depression severity, disorder type, and follow-up assessment period.

Appendix 6. The back-calculation methods for all the models

Checking consistency assumptions using back-calculation method[a] for the main findings

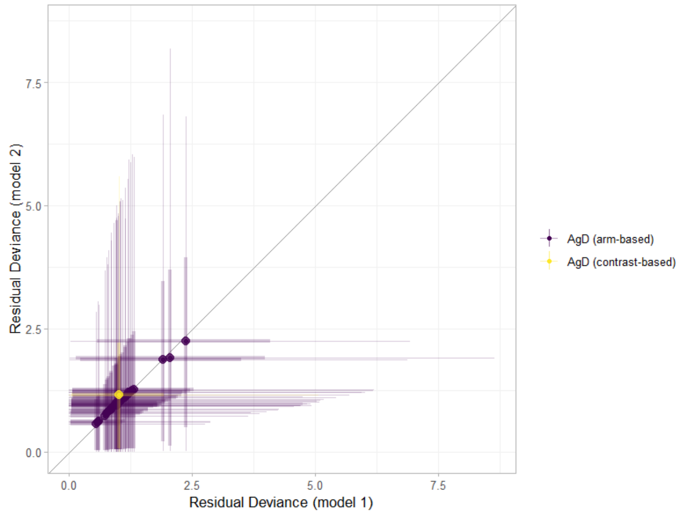


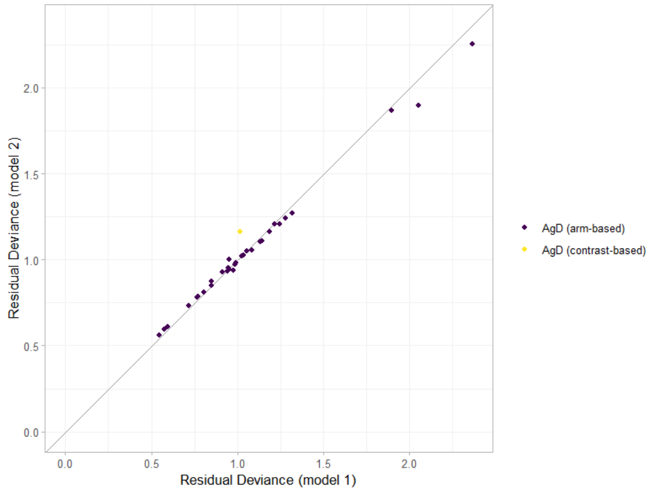


```
> dic_consistency
Residual deviance: 34.2 (on 32 data points)
               pD: 25.4
              DIC: 59.6
> dic_ume
Residual deviance: 33.9 (on 32 data points)
               pD: 25.6
              DIC: 59.5
```

[a] The *model 1* and *model 2* in this figure referred to a consistency model and a unrelated mean effect model, respectively for our defined model 1-6.

Reference: S Dias, N J Welton, D M Caldwell, A E Ades. Checking consistency in mixed treatment comparison meta-analysisStat Med. 2010 Mar 30;29(7-8):932-44.

Checking consistency assumptions using back-calculation method[a] for transitivity assumption of escitalopram Part I

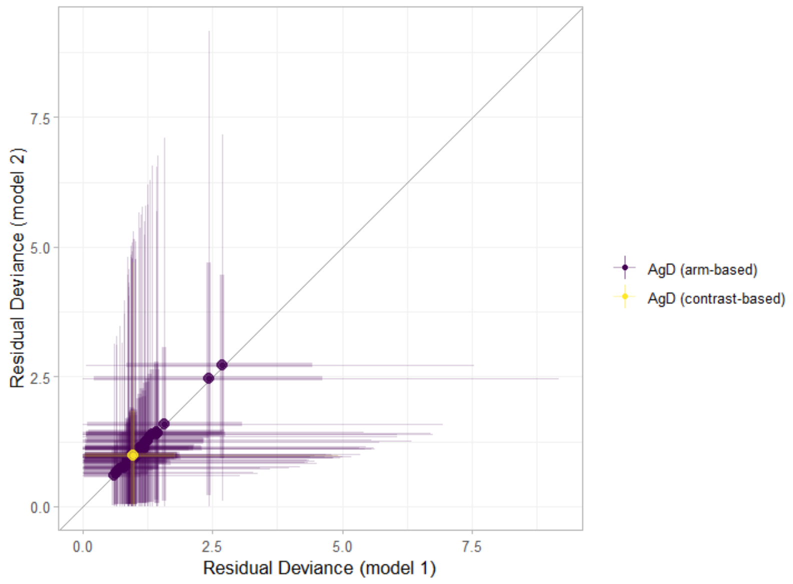


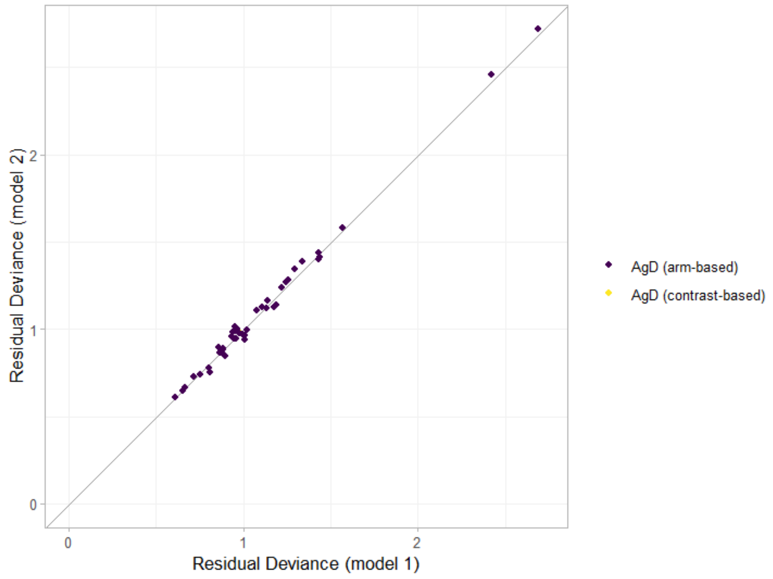


```
> dic_consistency
Residual deviance: 48 (on 44 data points)
            pD: 36.6
           DIC: 84.6
> dic_ume
Residual deviance: 48.1 (on 44 data points)
            pD: 36.5
           DIC: 84.6
```

[a] The *model 1* and *model 2* in this figure referred to a consistency model and a unrelated mean effect model, respectively for our defined model 1-6.

Reference: S Dias, N J Welton, D M Caldwell, A E Ades. Checking consistency in mixed treatment comparison meta-analysisStat Med. 2010 Mar 30;29(7-8):932-44.

Checking consistency assumptions using back-calculation method[a] for transitivity assumption of escitalopram Part II

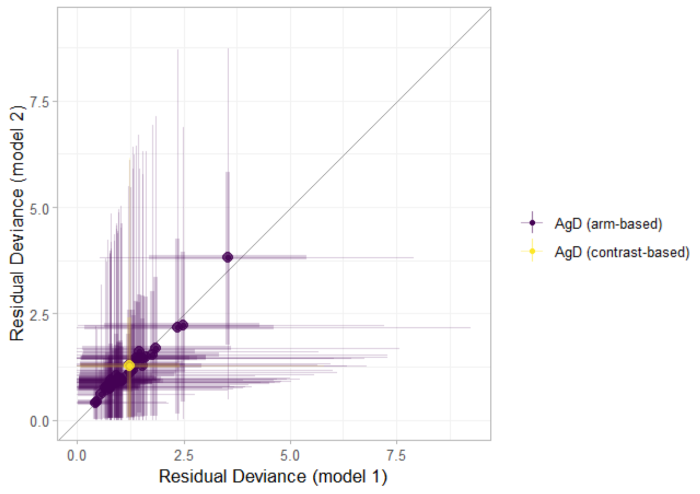


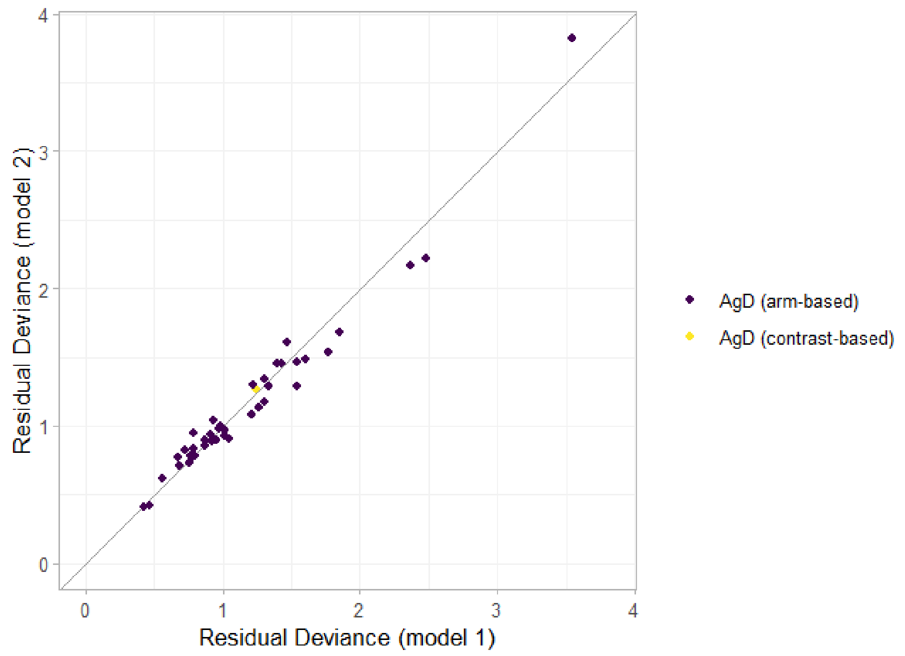


> dic_consistency
Residual deviance: 51.3 (on 44 data points)
pD: 32.9
DIC: 84.2
> dic_ume
Residual deviance: 50.4 (on 44 data points)
pD: 34.1
DIC: 84.5

[a] The *model 1* and *model 2* in this figure referred to a consistency model and a unrelated mean effect model, respectively for our defined model 1-6.

Reference: S Dias, N J Welton, D M Caldwell, A E Ades. Checking consistency in mixed treatment comparison meta-analysisStat Med. 2010 Mar 30;29(7-8):932-44.

Checking consistency assumptions using back-calculation method[a] for sensitivity analysis 1: including a study with enhancing blinding and minimizing expectancy

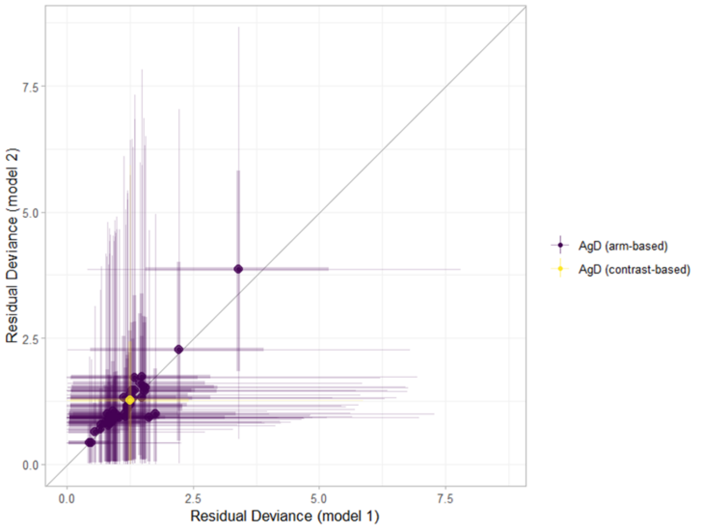


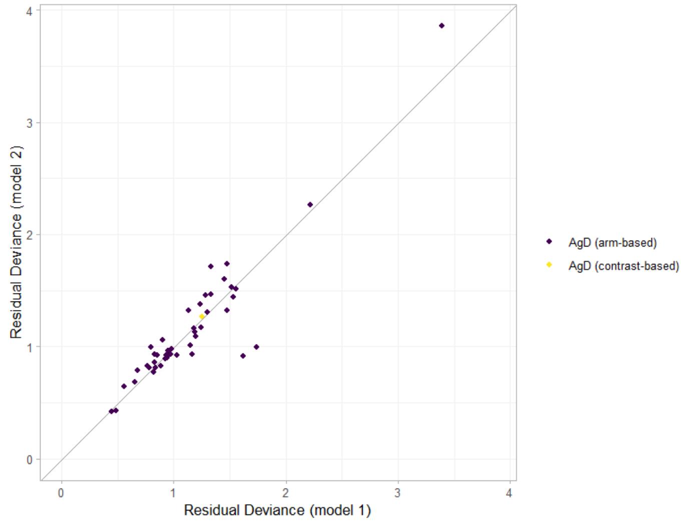


```
> dic_consistency
Residual deviance: 52.8 (on 46 data points)
               pD: 35.2
              DIC: 88
> dic_ume
Residual deviance: 52.8 (on 46 data points)
               pD: 35.9
              DIC: 88.7
```

[a] The *model 1* and *model 2* in this figure referred to a consistency model and a unrelated mean effect model, respectively for our defined model 1-6.

Reference: S Dias, N J Welton, D M Caldwell, A E Ades. Checking consistency in mixed treatment comparison meta-analysisStat Med. 2010 Mar 30;29(7-8):932-44.

Checking consistency assumptions using back-calculation method[a] for sensitivity analysis 2: including only patients with major depressive disorder

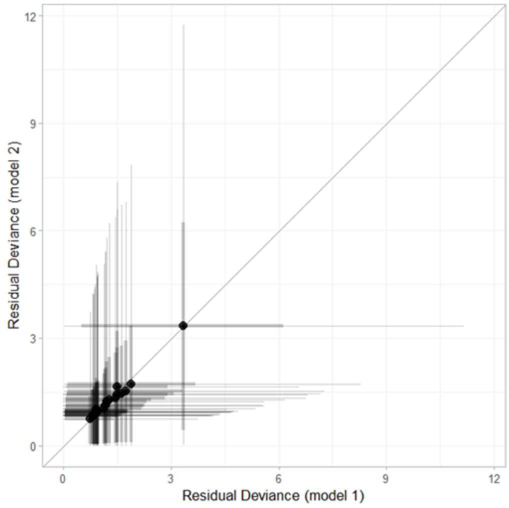


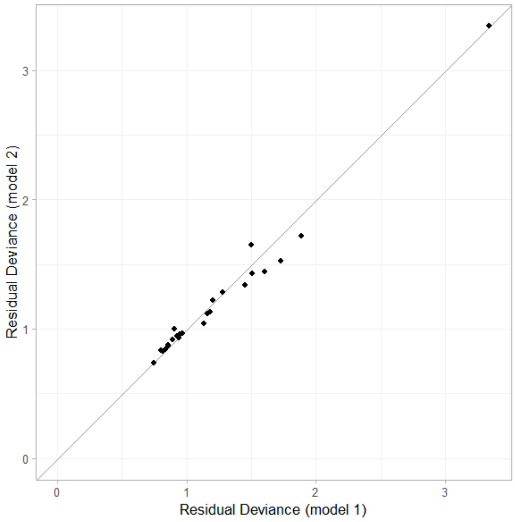


```
> dic_consistency
Residual deviance: 30.4 (on 25 data points)
               pD: 21.7
              DIC: 52.1
> dic_ume
Residual deviance: 29.9 (on 25 data points)
               pD: 22.3
              DIC: 52.2
```

[a] The *model 1* and *model 2* in this figure referred to a consistency model and a unrelated mean effect model, respectively for our defined model 1-6.

Reference: S Dias, N J Welton, D M Caldwell, A E Ades. Checking consistency in mixed treatment comparison meta-analysisStat Med. 2010 Mar 30;29(7-8):932-44.

Checking consistency assumptions using back-calculation method[a] for sensitivity analysis 3: excluding studies with high risk of bias

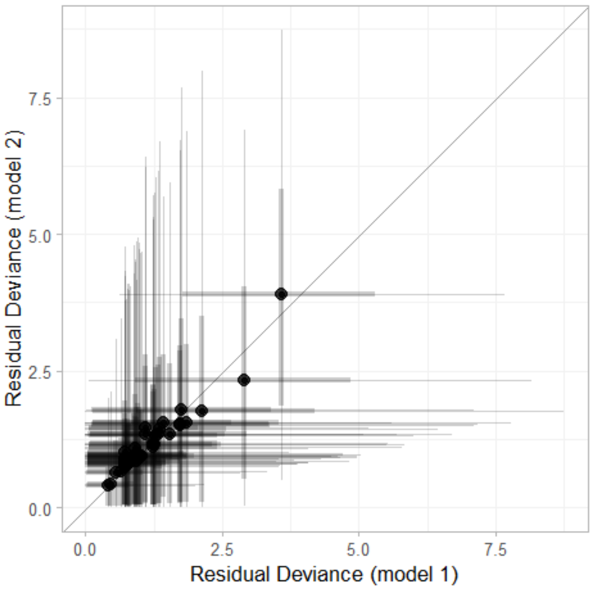


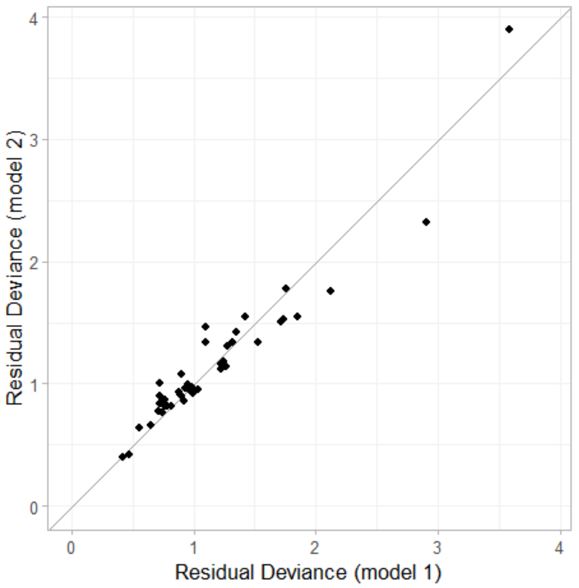


```
> dic_consistency
Residual deviance: 49.7 (on 43 data points)
               pD: 31.5
              DIC: 81.2
> dic_ume
Residual deviance: 49.6 (on 43 data points)
               pD: 33
              DIC: 82.6
```

[a] The *model 1* and *model 2* in this figure referred to a consistency model and a unrelated mean effect model, respectively for our defined model 1-6.

Reference: S Dias, N J Welton, D M Caldwell, A E Ades. Checking consistency in mixed treatment comparison meta-analysisStat Med. 2010 Mar 30;29(7-8):932-44.

Checking consistency assumptions using back-calculation method[a] for sensitivity analysis 4: adjusting for baseline depression severity

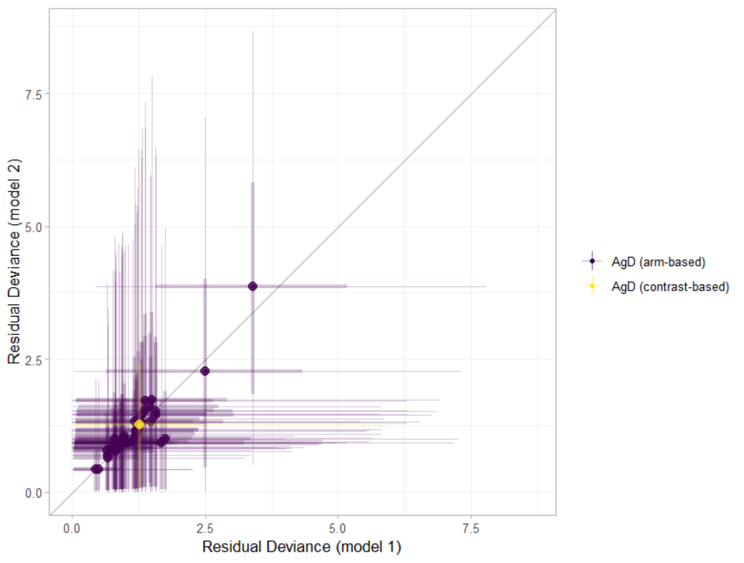


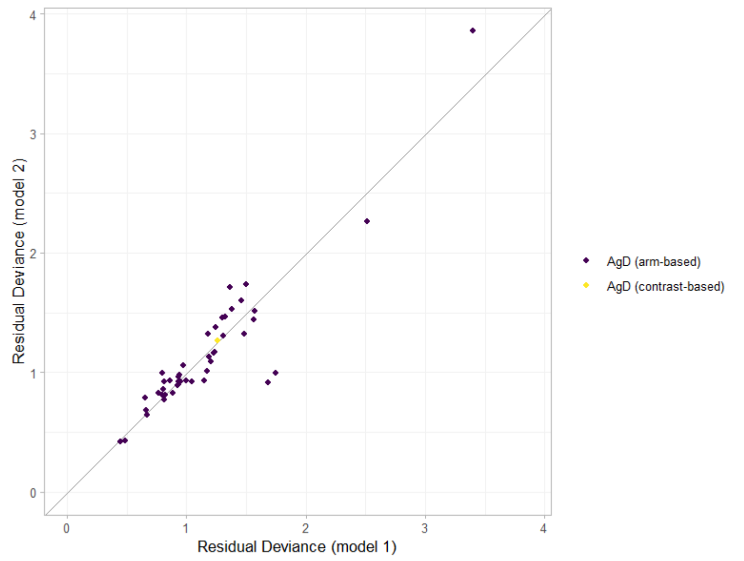


```
> dic_consistency
Residual deviance: 53.4 (on 46 data points)
               pD: 35.1
              DIC: 88.5
> dic_ume
Residual deviance: 52.8 (on 46 data points)
               pD: 35.9
              DIC: 88.7
```

[a] The *model 1* and *model 2* in this figure referred to a consistency model and a unrelated mean effect model, respectively for our defined model 1-6.

Reference: S Dias, N J Welton, D M Caldwell, A E Ades. Checking consistency in mixed treatment comparison meta-analysisStat Med. 2010 Mar 30;29(7-8):932-44.

Checking consistency assumptions using back-calculation method[a] for sensitivity analysis 5: using most conservative correlation coefficient of zero

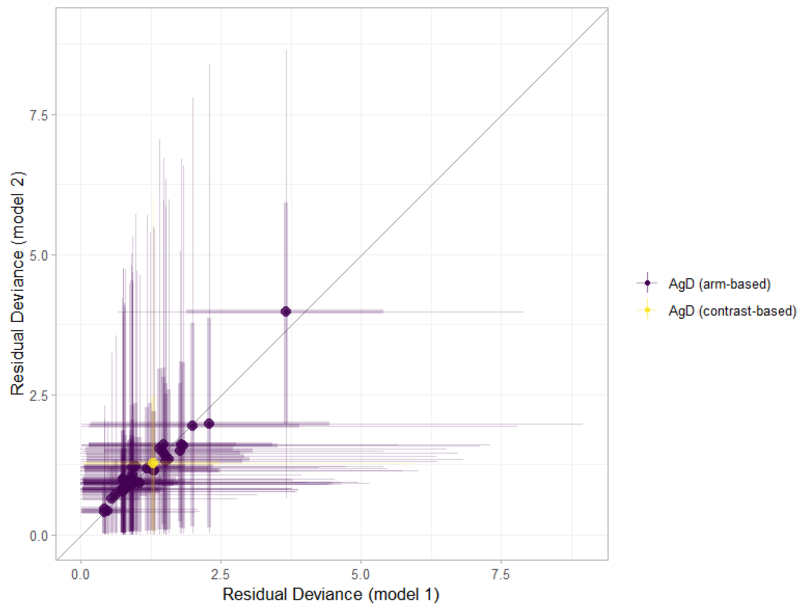


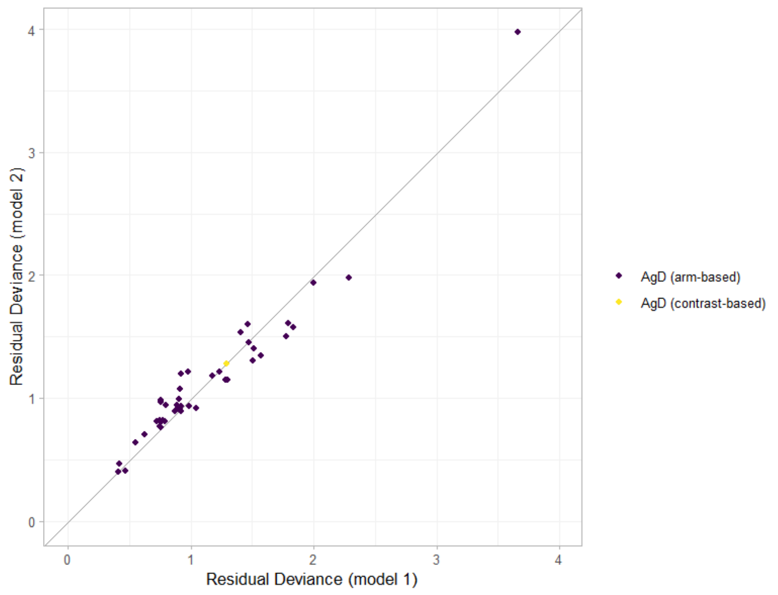


```
> dic_consistency
Residual deviance: 49.9 (on 44 data points)
               pD: 30.9
              DIC: 80.8
> dic_ume
Residual deviance: 50.1 (on 44 data points)
               pD: 32.8
              DIC: 82.9
```

[a] The *model 1* and *model 2* in this figure referred to a consistency model and a unrelated mean effect model, respectively for our defined model 1-6.

Reference: S Dias, N J Welton, D M Caldwell, A E Ades. Checking consistency in mixed treatment comparison meta-analysisStat Med. 2010 Mar 30;29(7-8):932-44.

## Appendix 7. Not-splitting methods

Checking consistency assumptions using node-splitting method[a] for the main findings

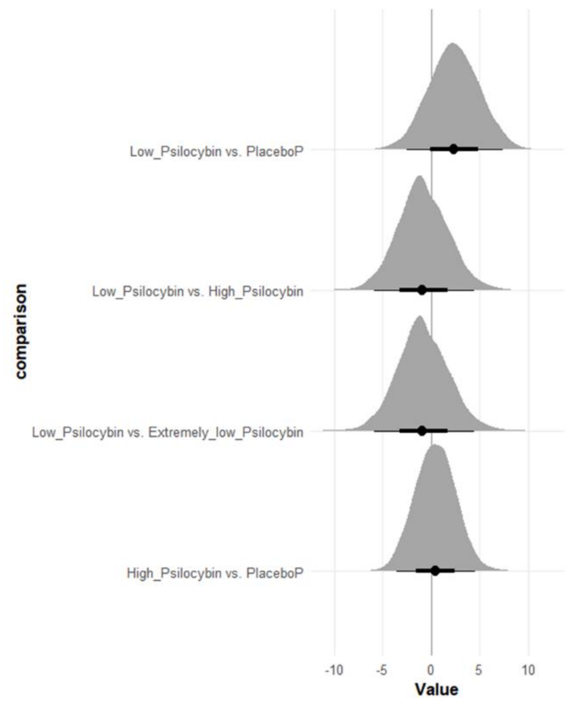


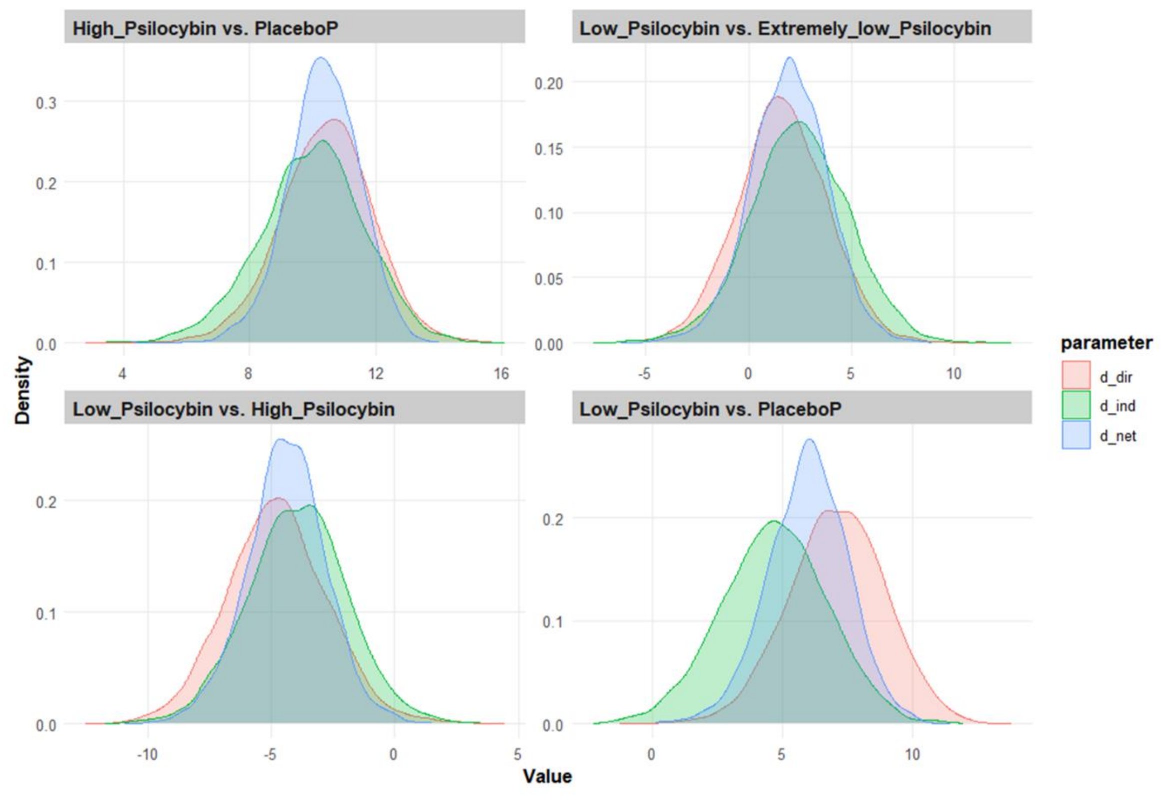


Bayesian p-value=0.83 for High_Psilocybin vs PlaceboP

Bayesian p-value=0.36 for Low_Psilocybin vs PlaceboP

Bayesian p-value=0.73 for Low_Psilocybin vs Extremely_low_Psilocybin

Bayesian p-value=0.73 for Low_Psilocybin vs High_Psilocybin

Checking consistency assumptions using node-splitting method[a] for assessing transitivity assumption of escitalopram Part I

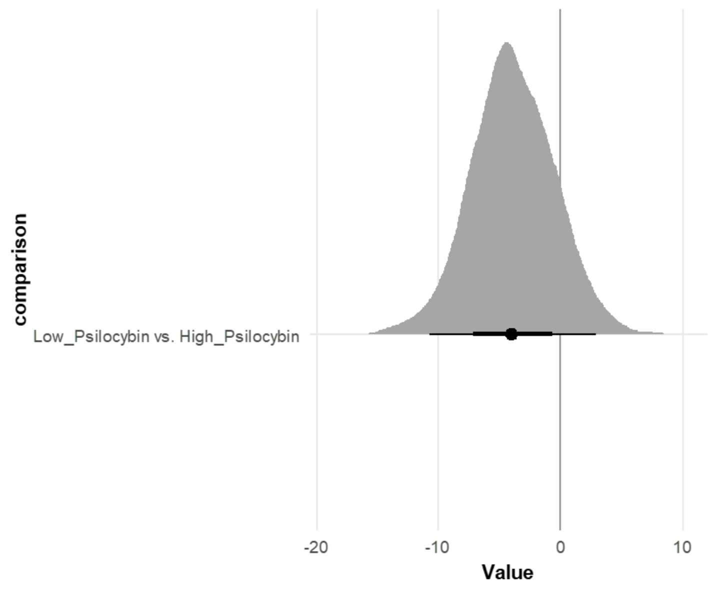


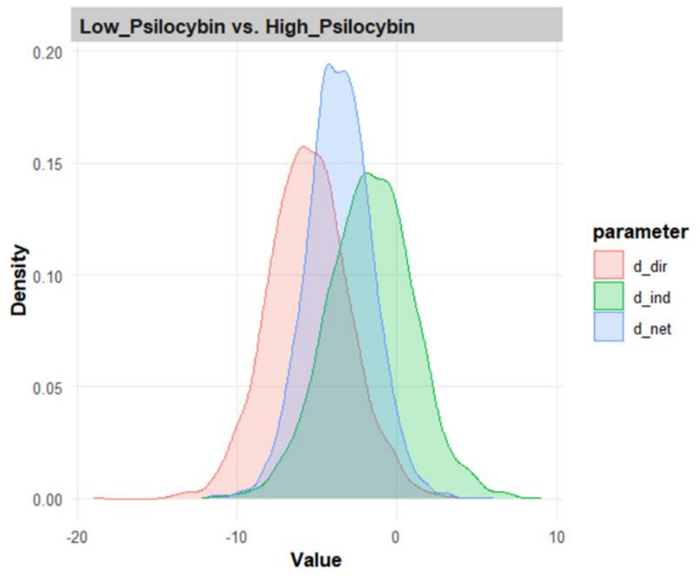


Bayesian p-value=0.26 for Low_Psilocybin vs High_Psilocybin

Checking consistency assumptions using via node-splitting method[a] for assessing transitivity assumption of escitalopram Part II

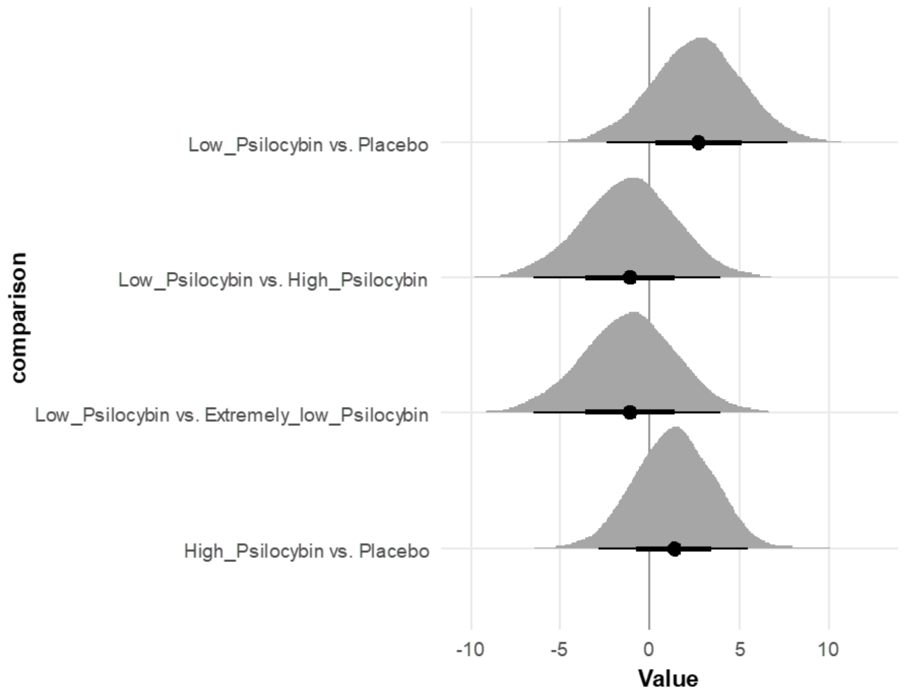


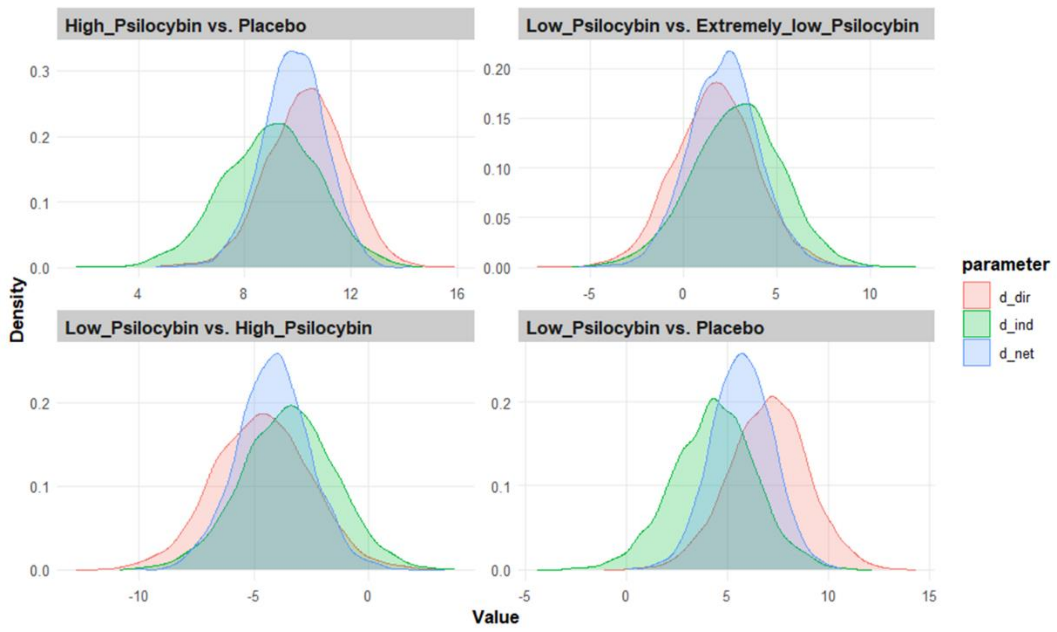


Bayesian p-value=0.65 for Low_Psilocybin vs Extremely_low_Psilocybin

Bayesian p-value=0.65 for Low_Psilocybin vs High_Psilocybin

Bayesian p-value=0.54 for High_Psilocybin vs Placebo

Bayesian p-value=0.29 for Low_Psilocybin vs Placebo

Checking consistency assumptions using via node-splitting method[a] for sensitivity analysis 1: including a study with enhancing blinding and minimizing expectancy

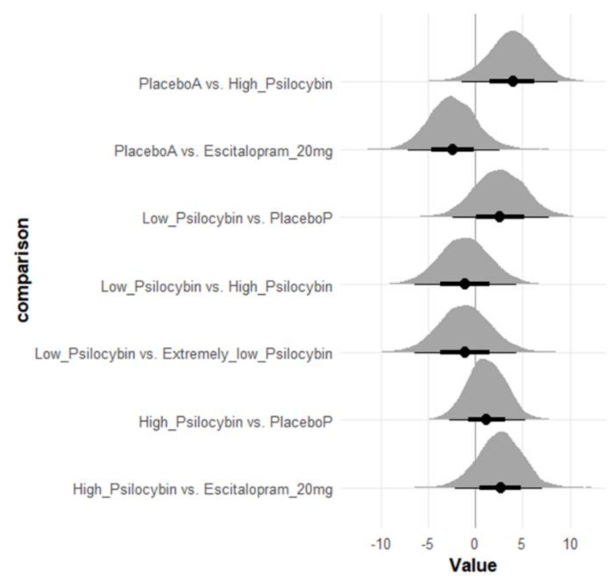


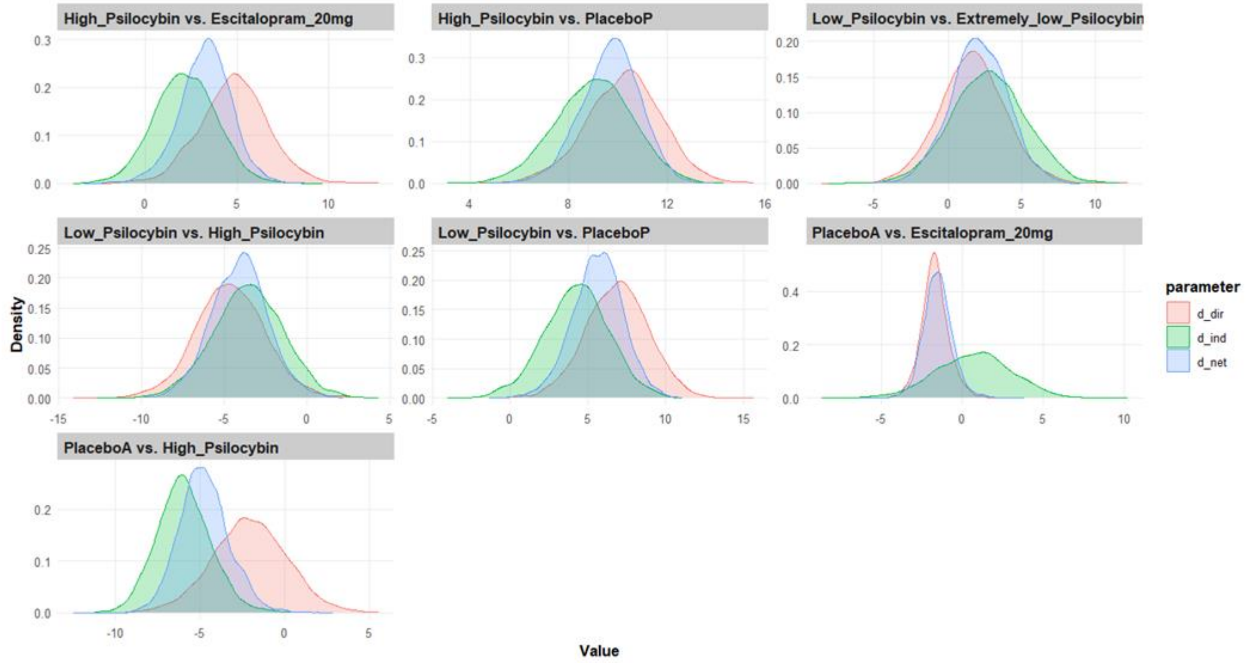


Bayesian p-value=0.55 for High_Psilocybin vs PlaceboP

Bayesian p-value=0.33 for Low_Psilocybin vs PlaceboP

Bayesian p-value=0.26 for High_Psilocybin vs Escitalopram_20mg

Bayesian p-value=0.31 for PlaceboA vs Escitalopram_20mg

Bayesian p-value=0.68 for Low_Psilocybin vs Extremely_low_Psilocybin

Bayesian p-value=0.68 for Low_Psilocybin vs High_Psilocybin

Bayesian p-value=0.14 for PlaceboA vs High_Psilocybin

Checking consistency assumptions using via node-splitting method[a] for sensitivity analysis 2: including only patients with major depressive disorder

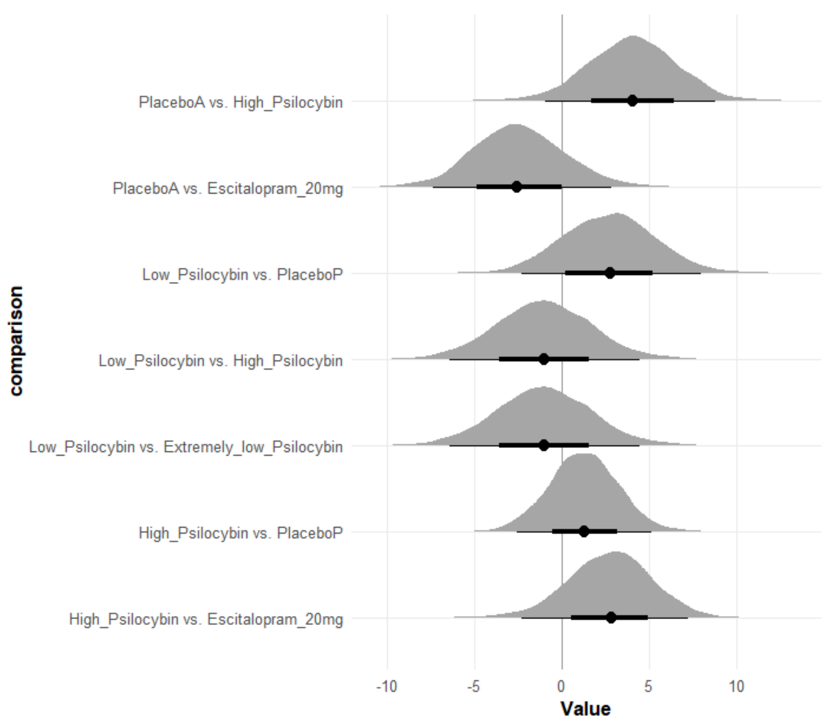


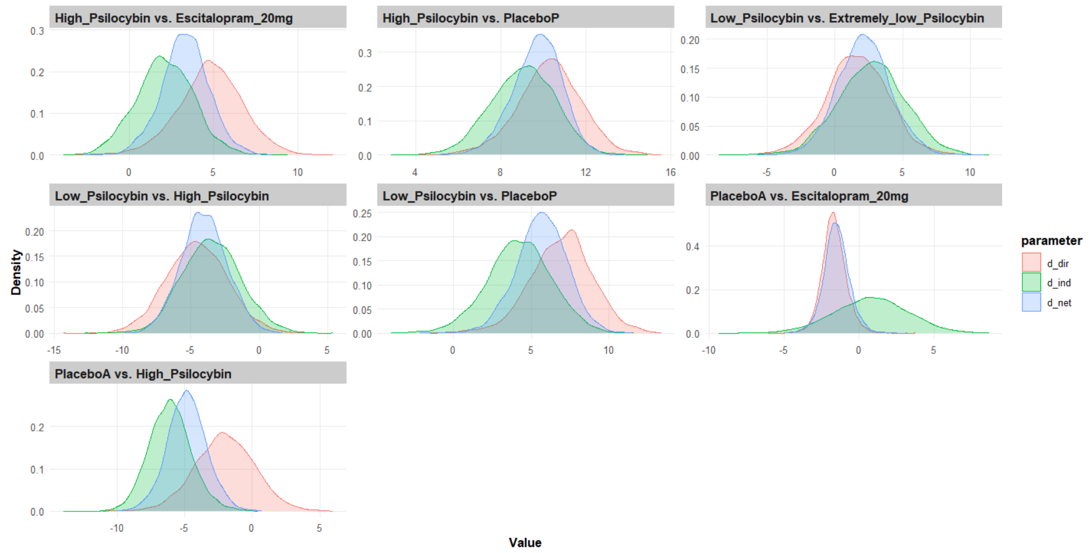


Bayesian p-value=0.52 for High_Psilocybin vs PlaceboP

Bayesian p-value=0.31 for Low_Psilocybin vs PlaceboP

Bayesian p-value=0.25 for High_Psilocybin vs Escitalopram_20mg

Bayesian p-value=0.33 for PlaceboA vs Escitalopram_20mg

Bayesian p-value=0.69 for Low_Psilocybin vs Extremely_low_Psilocybin

Bayesian p-value=0.69 for Low_Psilocybin vs High_Psilocybin

Bayesian p-value=0.11 for PlaceboA vs High_Psilocybin

Checking consistency assumptions using via node-splitting method[a] for sensitivity analysis 3: excluding studies with high risk of bias

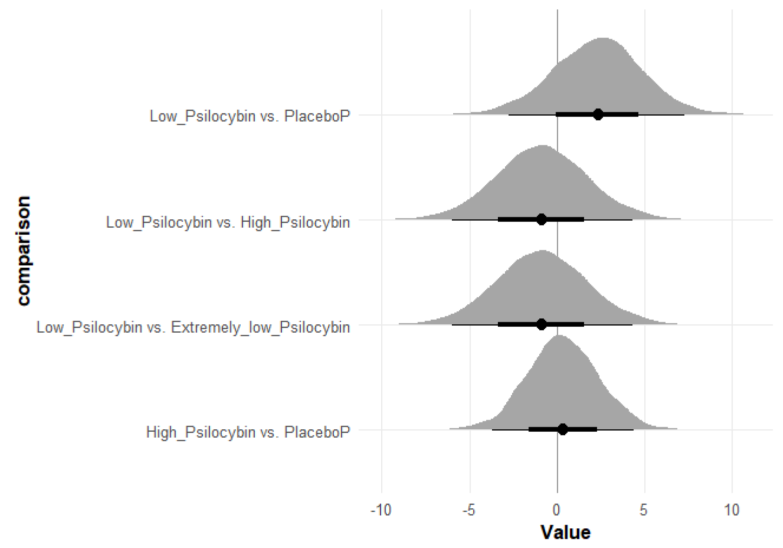


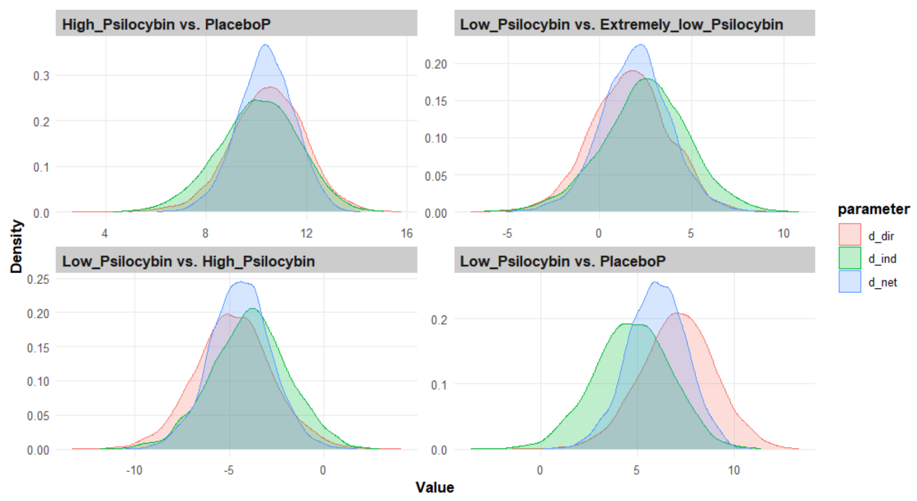


Bayesian p-value=0.86 for High_Psilocybin vs PlaceboP

Bayesian p-value=0.35 for Low_Psilocybin vs PlaceboP

Bayesian p-value=0.73 for Low_Psilocybin vs Extremely_low_Psilocybin

Bayesian p-value=0.73 for Low_Psilocybin vs High_Psilocybin

Checking consistency assumptions using via node-splitting method[a] for sensitivity analysis 4: adjusting for baseline depression severity

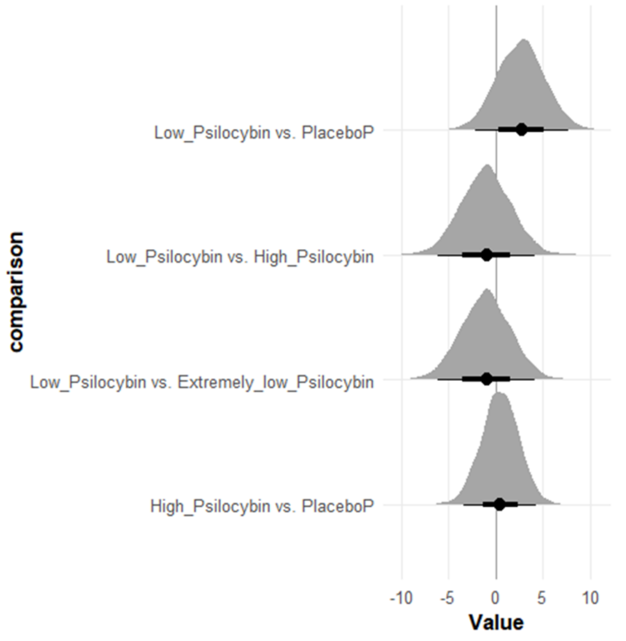


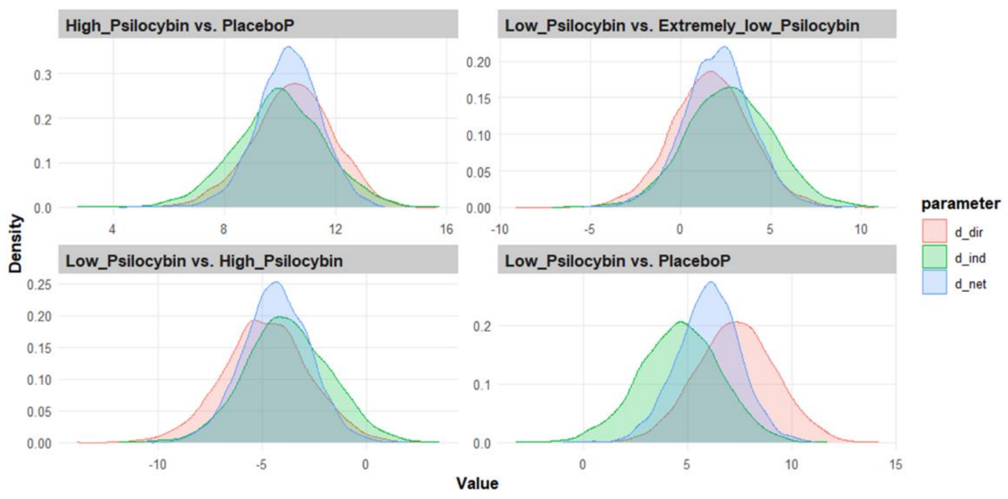


Bayesian p-value=0.81 for High_Psilocybin vs PlaceboP

Bayesian p-value=0.29 for Low_Psilocybin vs PlaceboP

Bayesian p-value=0.68 for Low_Psilocybin vs Extremely_low_Psilocybin

Bayesian p-value=0.68 for Low_Psilocybin vs High_Psilocybin

Checking consistency assumptions using via node-splitting method[a] for sensitivity analysis 5: using most conservative correlation coefficient of zero

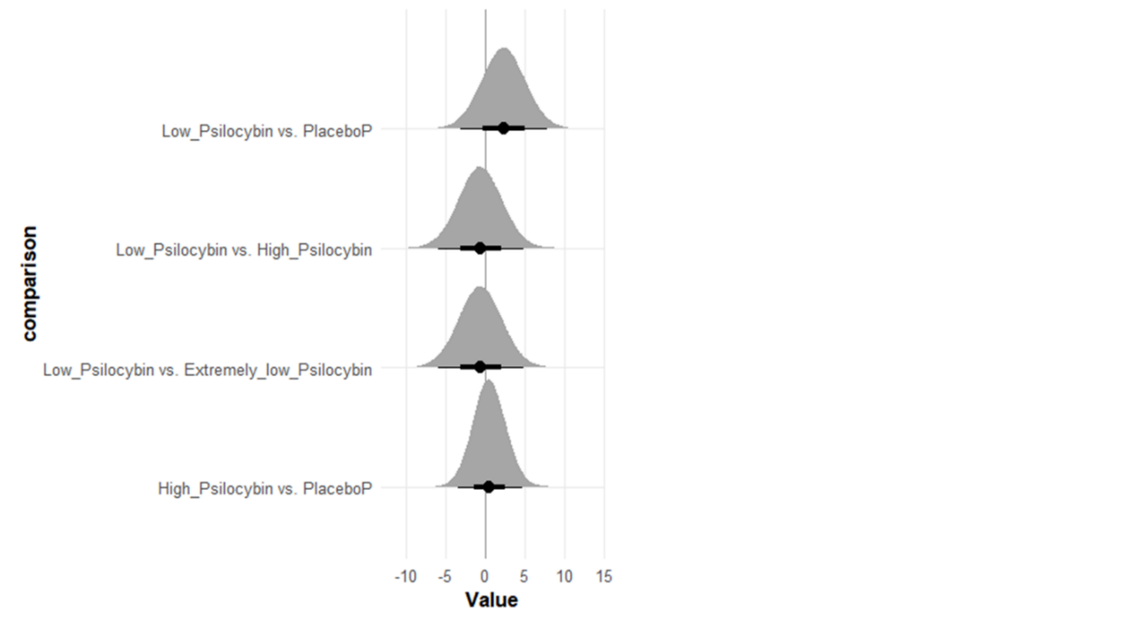


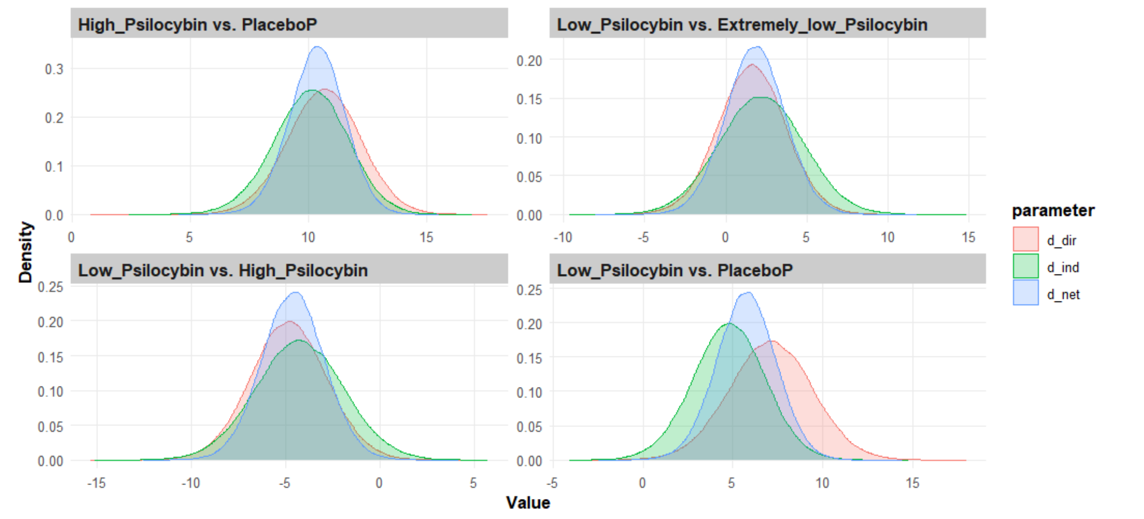


Bayesian p-value=0.79 for High_Psilocybin vs PlaceboP

Bayesian p-value=0.4 for Low_Psilocybin vs PlaceboP

Bayesian p-value=0.83 for Low_Psilocybin vs Extremely_low_Psilocybin

Bayesian p-value=0.83 for Low_Psilocybin vs High_Psilocybin
